# Supplementary material for: Simulated sample splitting approach to address biases due to instrument selection and participant overlap in two-sample Mendelian Randomization studies
Source: PLoS Genet. 2026 May 8;22(5):e1011949. doi: 10.1371/journal.pgen.1011949 (PMC13178975; doi:10.1371/journal.pgen.1011949)
Supplement: S1 Text — This file contains supplementary figures, tables as well as important derivations. (PDF) [file pgen.1011949.s001.pdf]

Supplement for: Simulated sample splitting approach to address biases due to instrument selection and participant overlap in two-sample Mendelian Randomization studies

Amanda Forde[1*], Gibran Hemani[2,3], John Ferguson[1]

[1]School of Mathematical and Statistical Sciences, University of Galway, Galway, Ireland

[2]NIHR Bristol Biomedical Research Centre, University Hospitals Bristol and Weston NHS Foundation Trust and University of Bristol, Bristol, UK

[3]MRC Integrative Epidemiology Unit (IEU), Bristol Medical School, University of Bristol, Bristol, UK

* Corresponding author

E-mail: amanda.forde@universityofgalway.ie

Contents

Supplementary Figures 1

Supplementary Tables 16

Derivation of conditional distribution 26

Marginal distribution of $(\hat{\beta}_X, \hat{\beta}_Y)$ 26

Conditional distribution of $(\hat{\beta}_X^1, \hat{\beta}_Y^1)$ 28

Inserting randomness 31

Binary outcome 32

Derivation of standard error for MR-SimSS estimator 34

Estimating correlation 35

Variant pre-filtering 36

Supplementary Figures

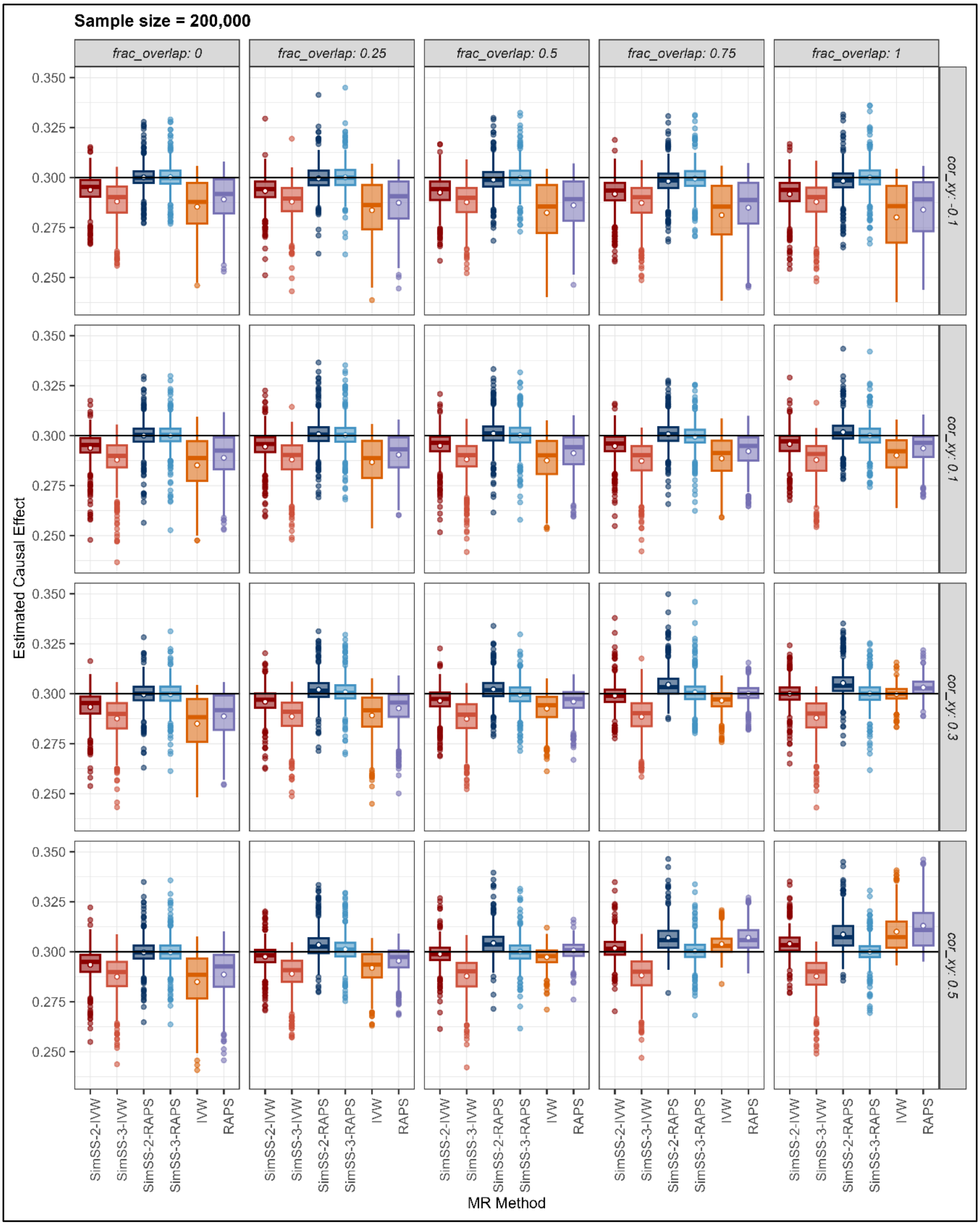

Fig A: Estimated causal effect for each method and simulation setting with $n_X = n_Y = 200{,}000$, collapsed over heritability and proportion of true effect variants, across 100 simulated pairs of exposure and outcome GWAS summary statistics for each setting.

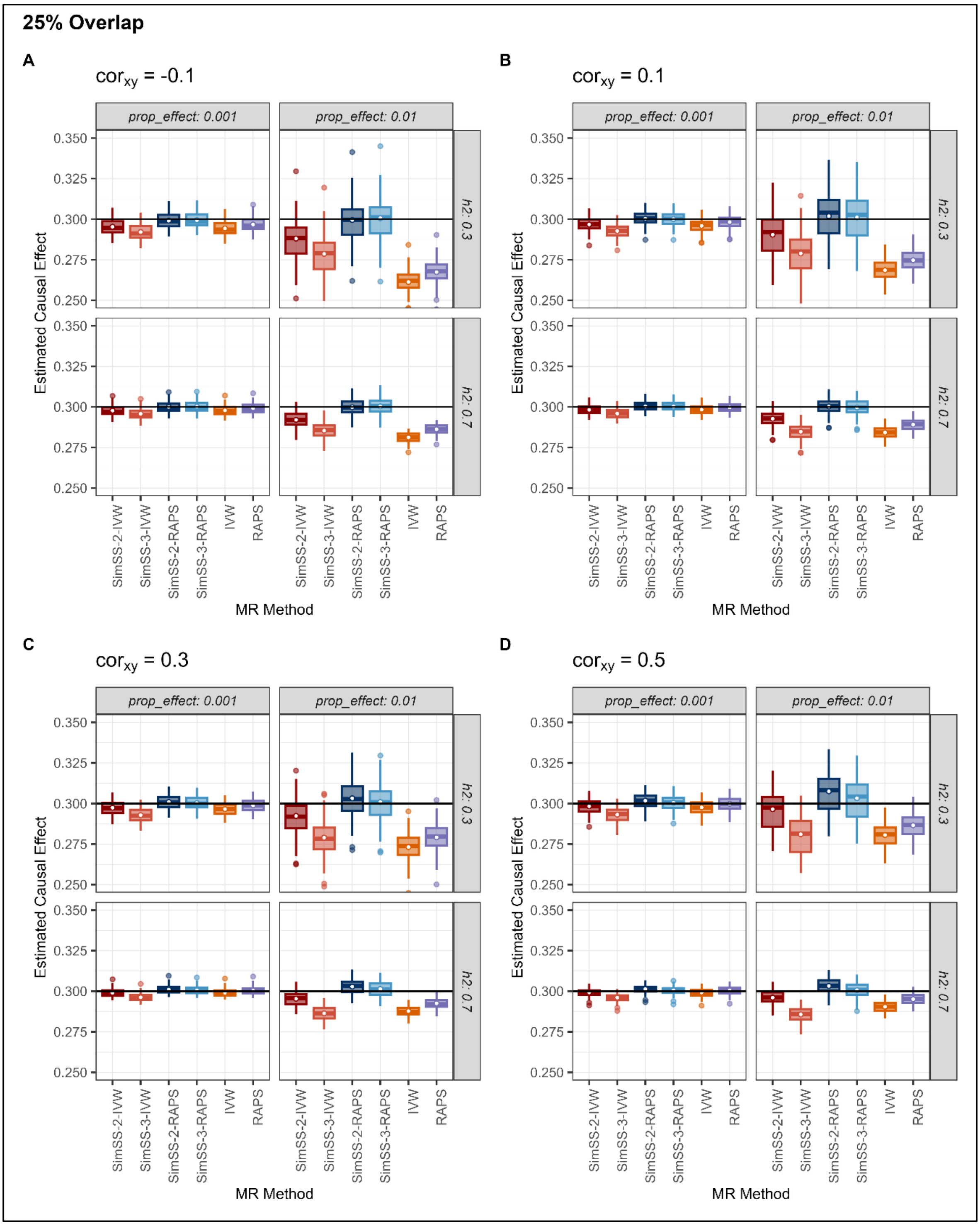

Fig B: Estimated causal effect for each method and simulation setting with $n_X = n_Y = 200{,}000$ and 25% overlap, averaged over 100 simulated pairs of exposure and outcome GWAS summary statistics for each setting.

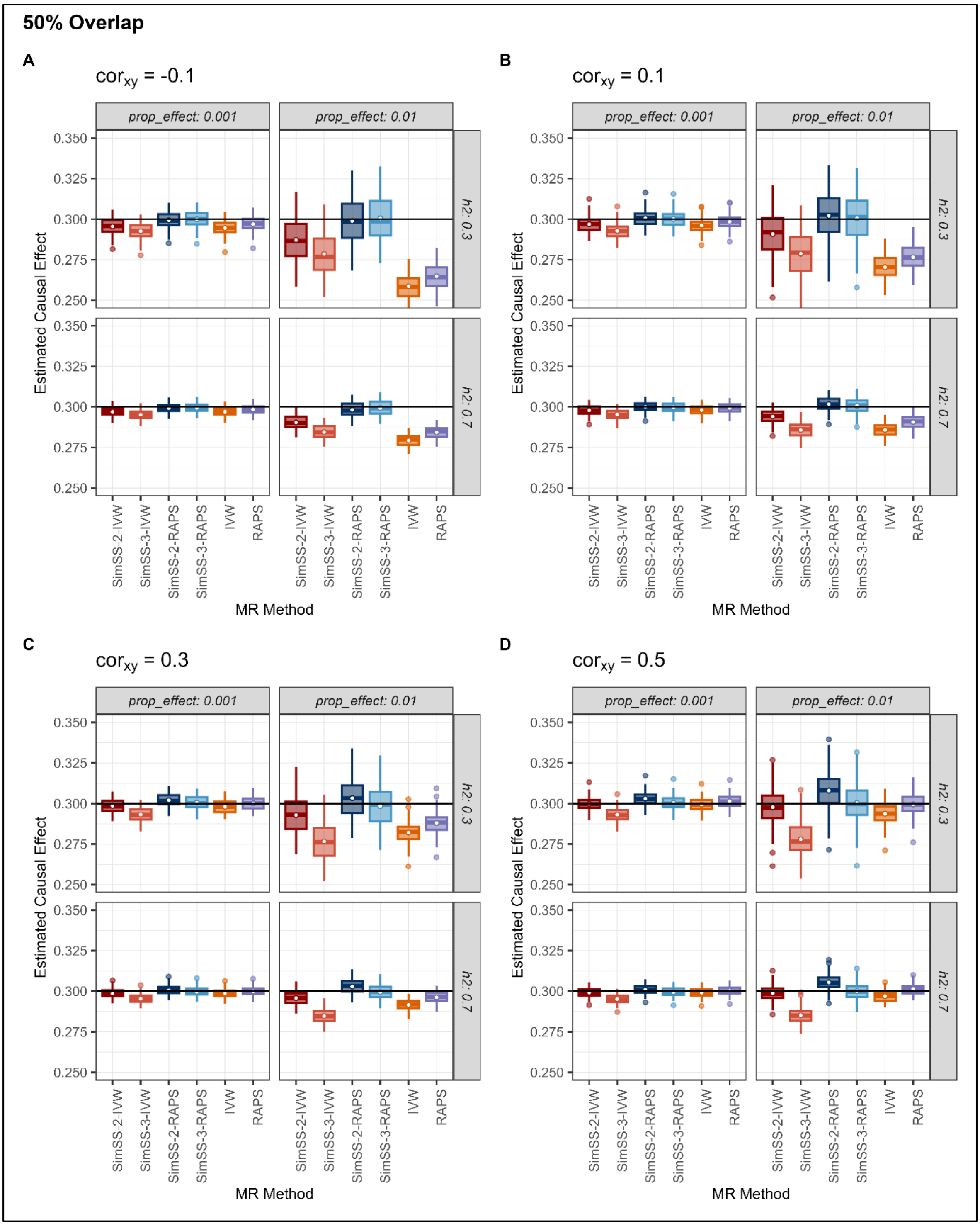

Fig C: Estimated causal effect for each method and simulation setting with $n_X = n_Y = 200{,}000$ and 50% overlap, averaged over 100 simulated pairs of exposure and outcome GWAS summary statistics for each setting.

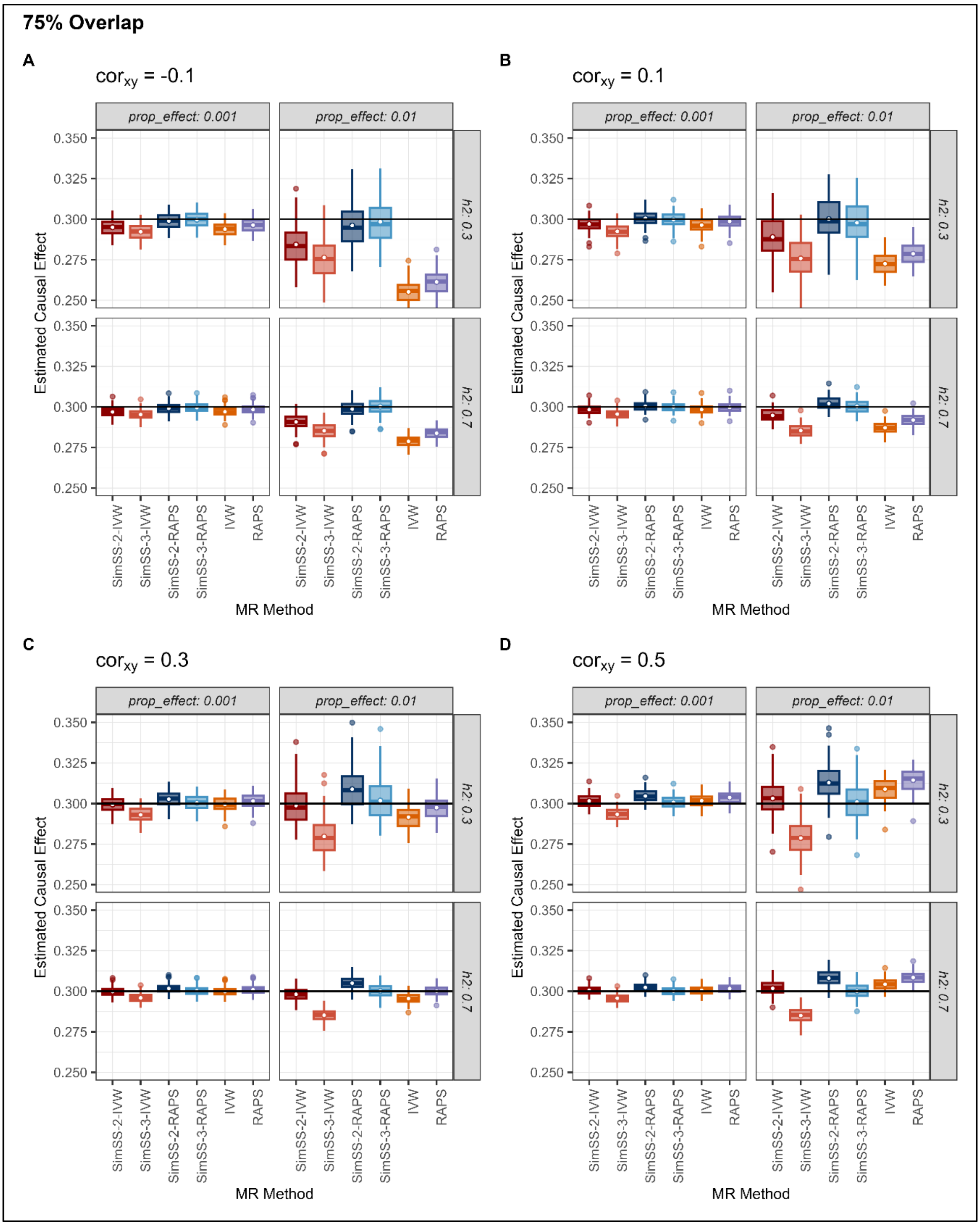

Fig D: Estimated causal effect for each method and simulation setting with $n_X = n_Y = 200{,}000$ and 75% overlap, averaged over 100 simulated pairs of exposure and outcome GWAS summary statistics for each setting.

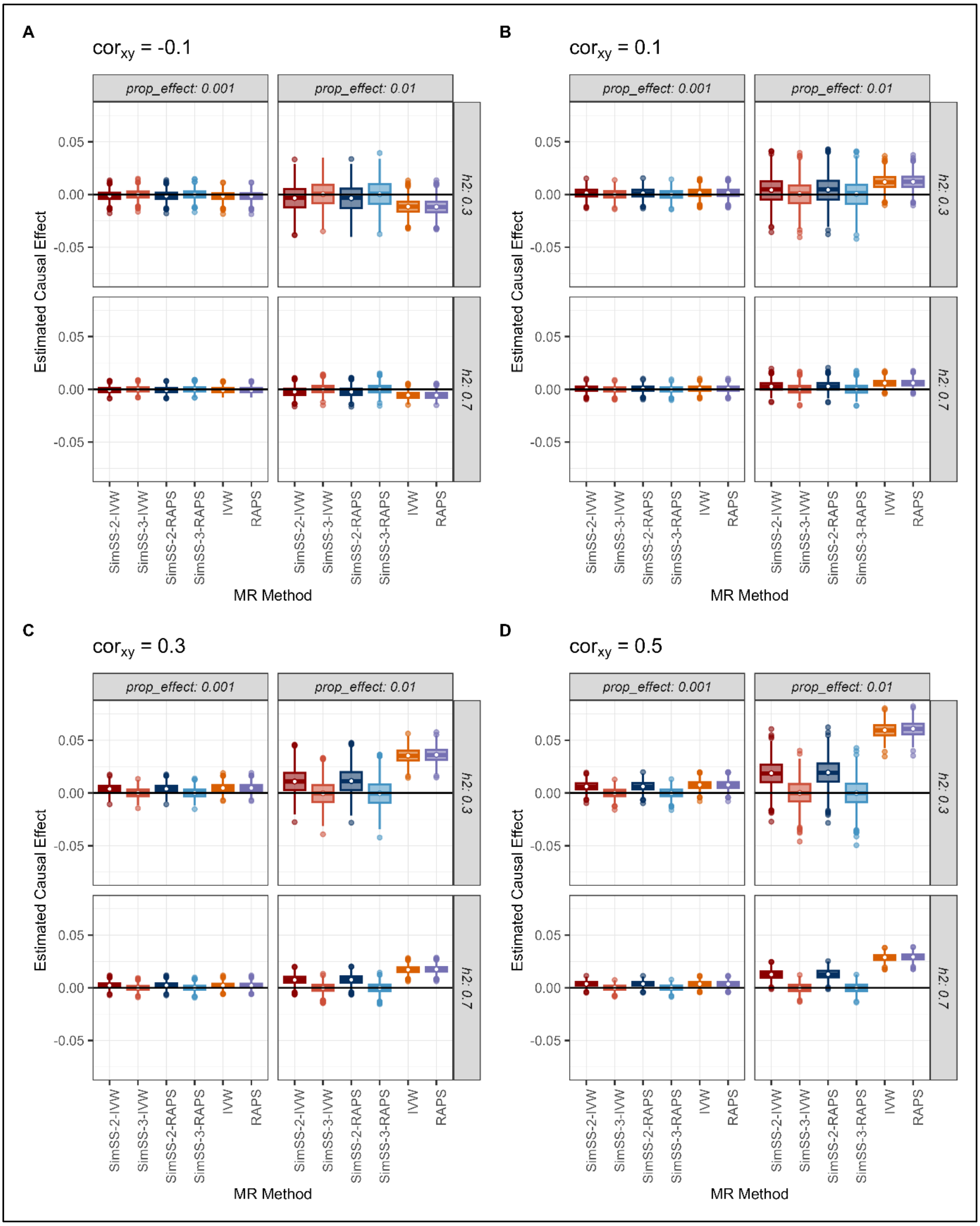

Fig E: Estimated causal effect for each method and simulation setting with true causal effect = 0 and fully overlapping samples of size $n_X = n_Y = 200{,}000$, across 1000 simulated pairs of exposure and outcome GWAS summary statistics for each setting.

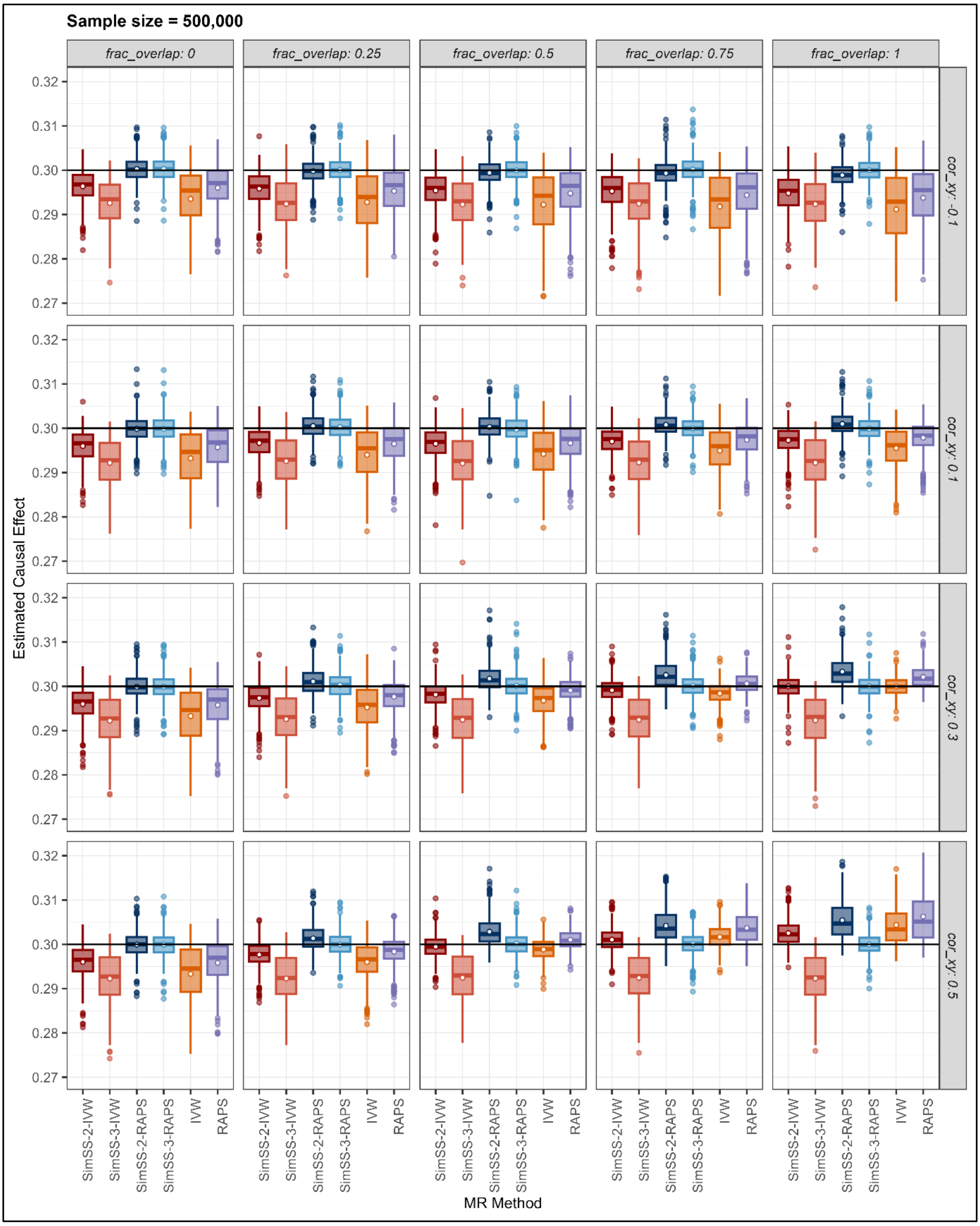

Fig F: Estimated causal effect for each method and simulation setting with $n_X = n_Y = 500{,}000$, collapsed over heritability and proportion of true effect variants, across 100 simulated pairs of exposure and outcome GWAS summary statistics for each setting.

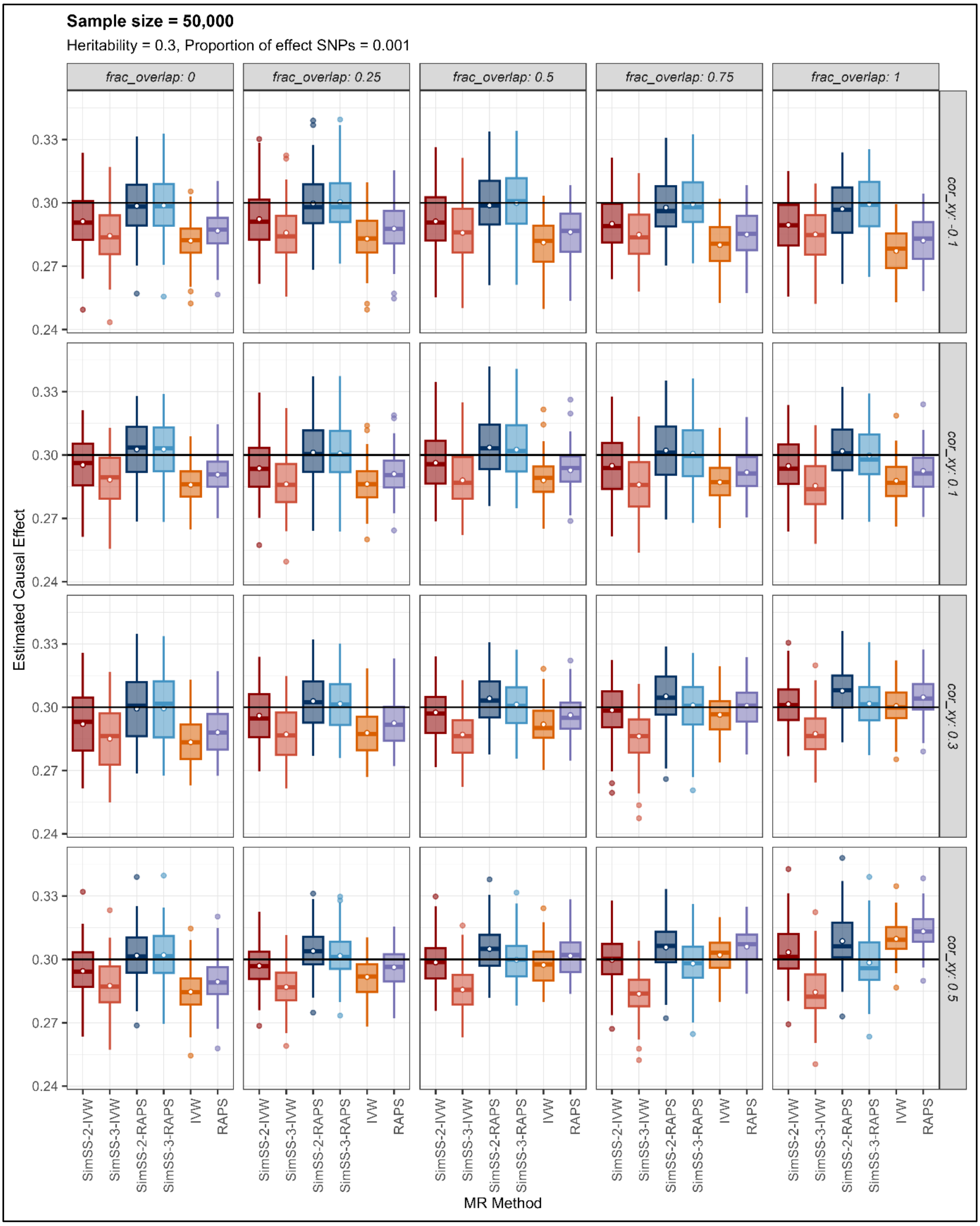

Fig G: Estimated causal effect for each method and simulation setting with $n_X = n_Y = 50{,}000$, heritability = 0.3 and proportion of true effect variants = 0.001, across 100 simulated pairs of exposure and outcome GWAS summary statistics for each setting.

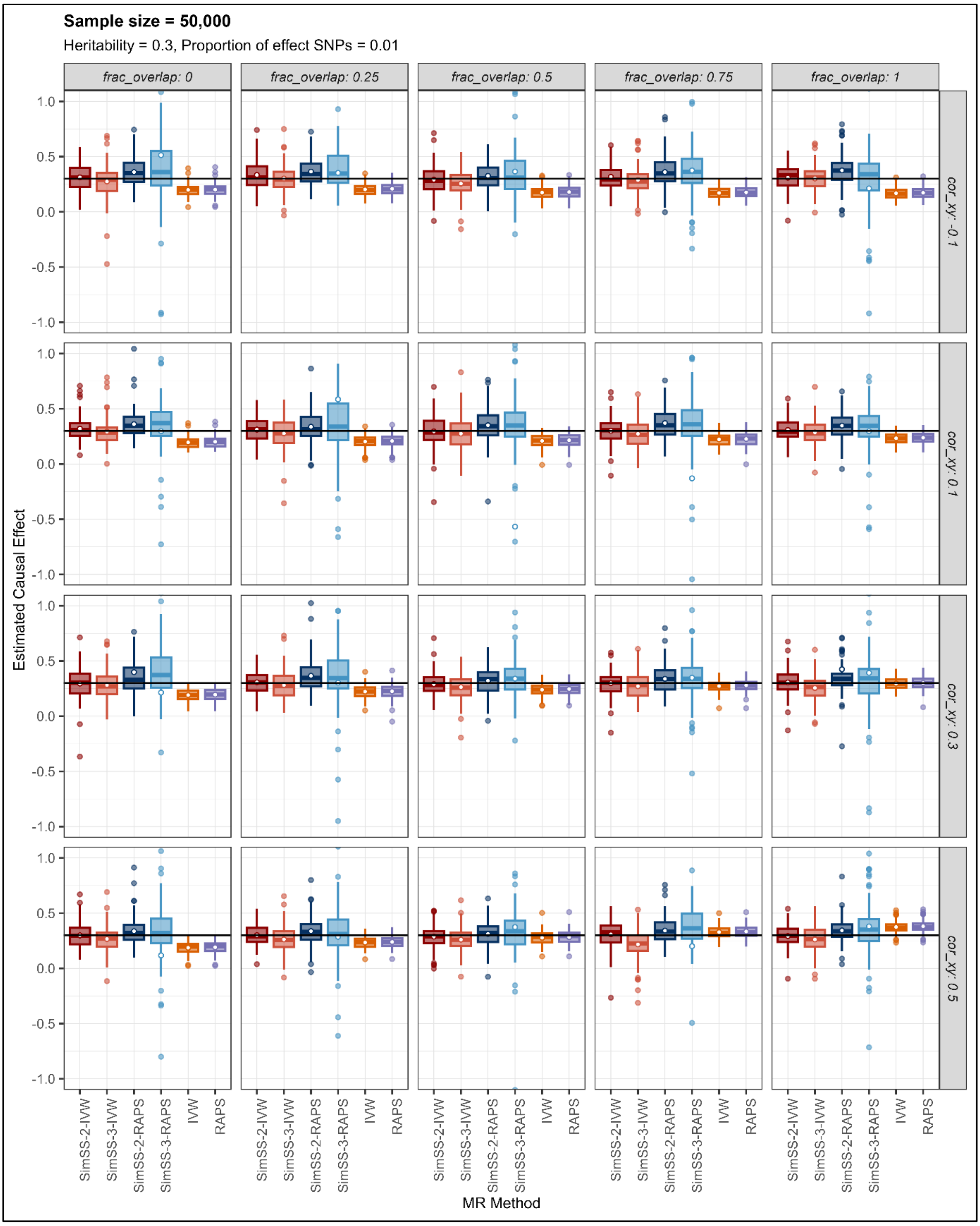

Fig H: Estimated causal effect for each method and simulation setting with $n_X = n_Y = 50{,}000$, heritability = 0.3 and proportion of true effect variants = 0.01, across 100 simulated pairs of exposure and outcome GWAS summary statistics for each setting.

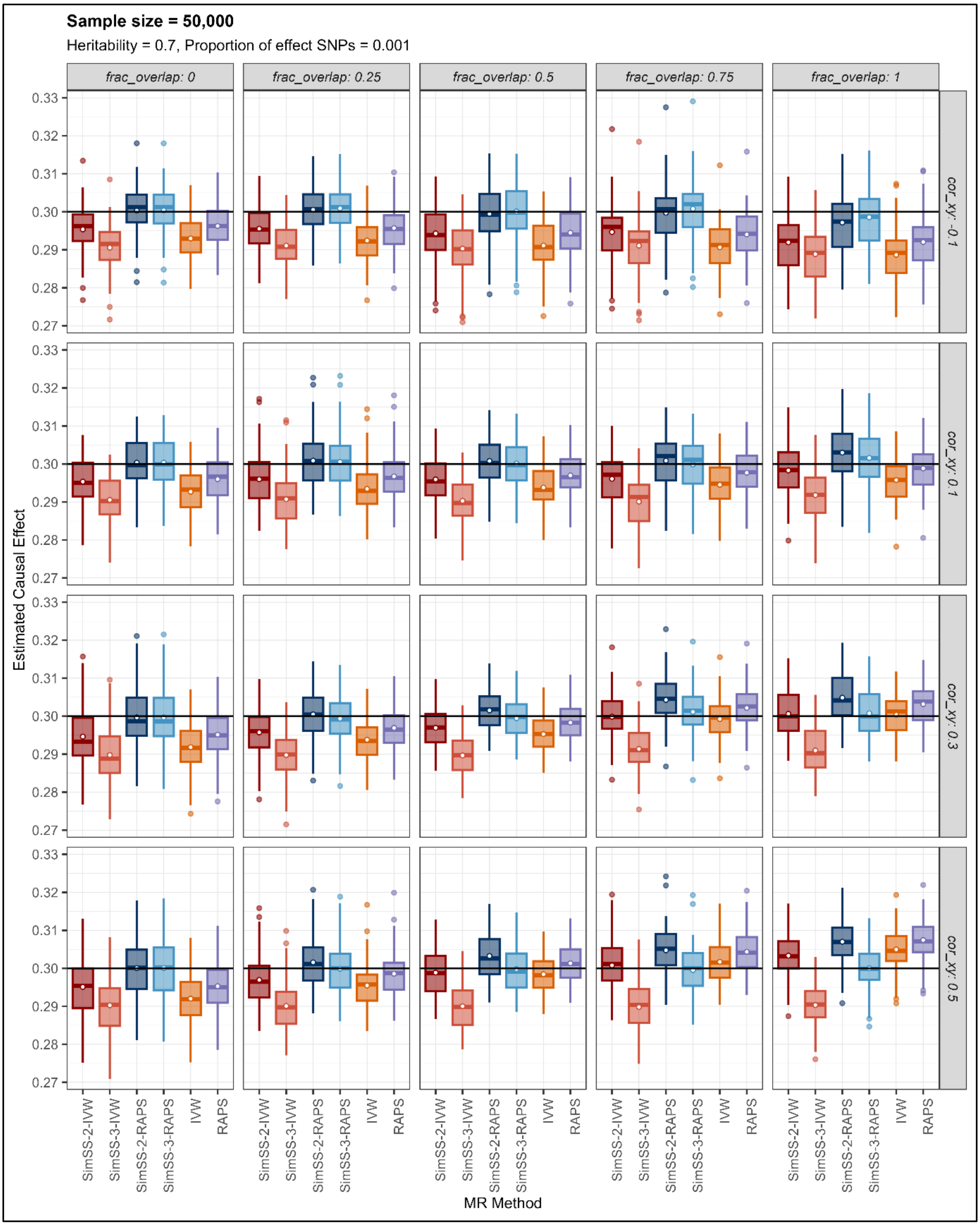

Fig I: Estimated causal effect for each method and simulation setting with $n_X = n_Y = 50{,}000$, heritability = 0.7 and proportion of true effect variants = 0.001, across 100 simulated pairs of exposure and outcome GWAS summary statistics for each setting.

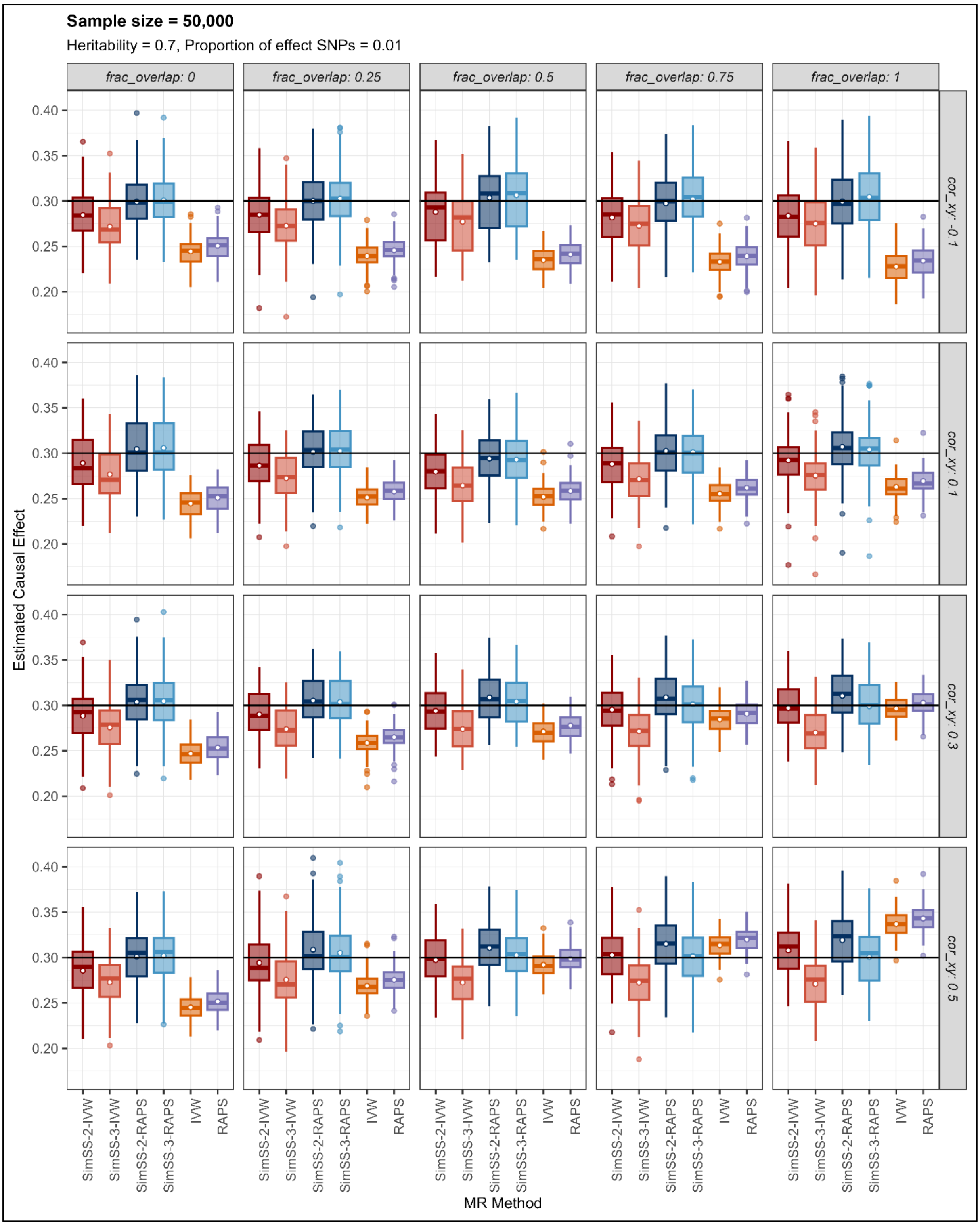

Fig J: Estimated causal effect for each method and simulation setting with $n_X = n_Y = 50{,}000$, heritability = 0.7 and proportion of true effect variants = 0.01, across 100 simulated pairs of exposure and outcome GWAS summary statistics for each setting.

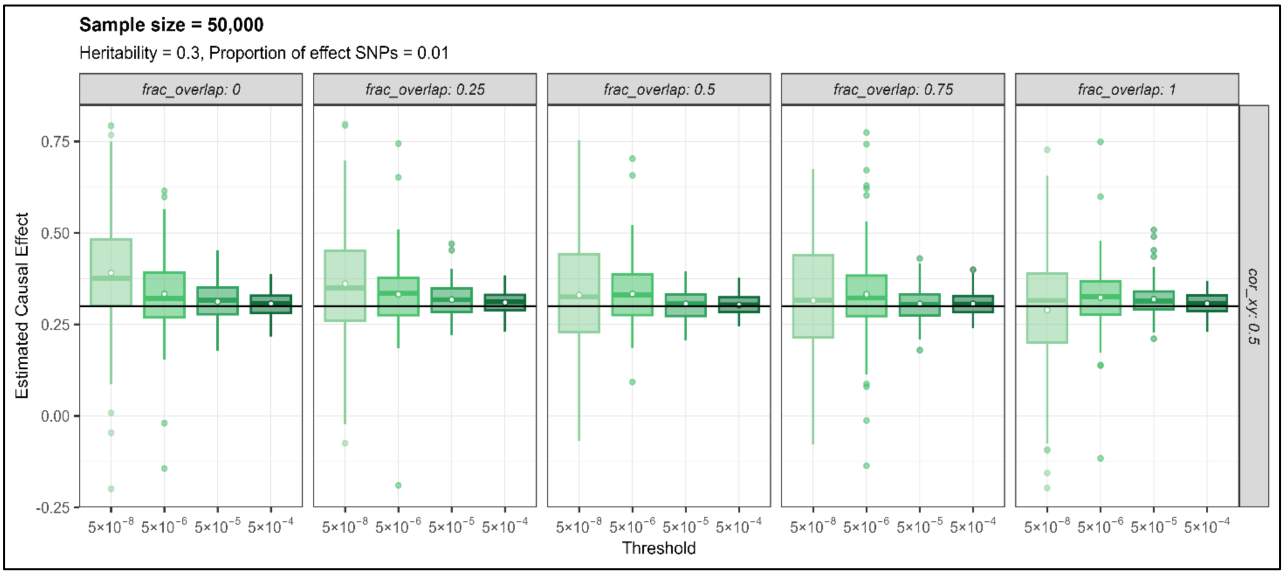

Fig K: Estimated causal effect for SimSS-3-RAPS with four different thresholds implemented for varying overlap fractions with $n_X = n_Y = 50{,}000$, heritability = 0.3, proportion of true effect variants = 0.01 and exposure-outcome correlation = 0.5 across 100 simulated pairs of exposure and outcome GWAS summary statistics for each setting.

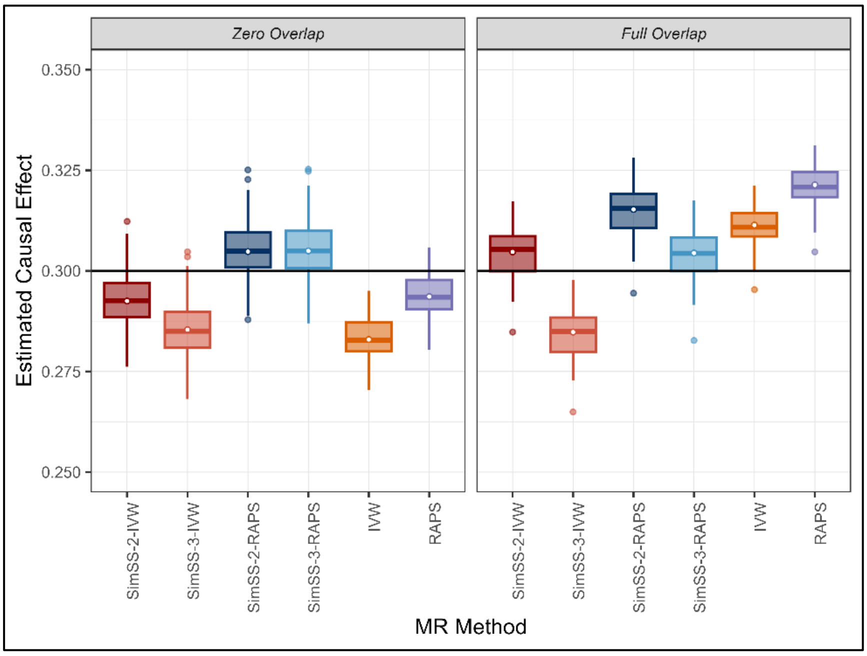

Fig L: Estimated causal effect for each method under a balanced pleiotropy setting ($\mu_\alpha = 0$) with $n_X = n_Y = 200{,}000$, heritability = 0.7, proportion of true effect variants = 0.01 and proportion of effect variants exhibiting pleiotropy = 0.2, across 100 simulated pairs of exposure and outcome GWAS summary statistics for each setting.

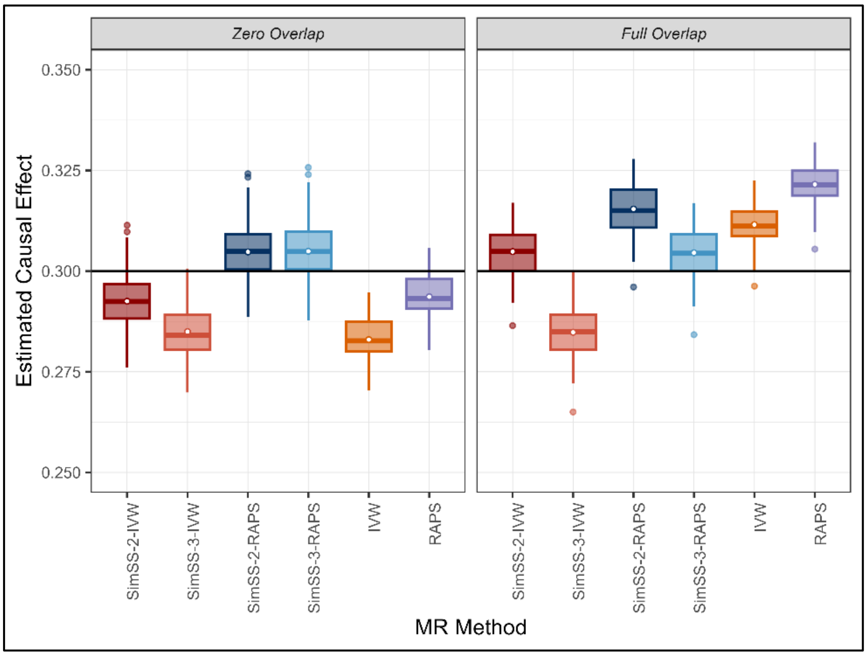

Fig M: Estimated causal effect for each method under a directional pleiotropy setting ($\mu_\alpha = 0.2$) with $n_X = n_Y = 200{,}000$, heritability = 0.7, proportion of true effect variants = 0.01 and proportion of effect variants exhibiting pleiotropy = 0.2, across 100 simulated pairs of exposure and outcome GWAS summary statistics for each setting.

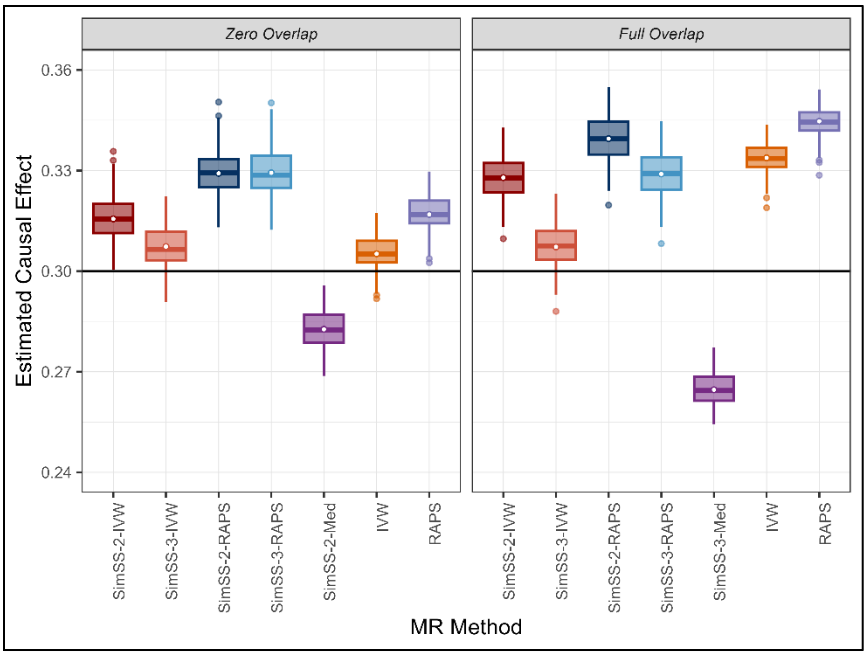

Fig N: Estimated causal effect for each method under a directional positively-correlated pleiotropy setting ($\mu_\alpha = 0.2, \text{corr}(\alpha, \beta_X) = 0.2$) with $n_X = n_Y = 200{,}000$, heritability = 0.7, proportion of true effect variants = 0.01 and proportion of effect variants exhibiting pleiotropy = 0.2, across 100 simulated pairs of exposure and outcome GWAS summary statistics for each setting. SimSS-2-Med and SimSS-3-Med = 2-split and 3-split versions, respectively, of MR-SimSS using MR weighted median.

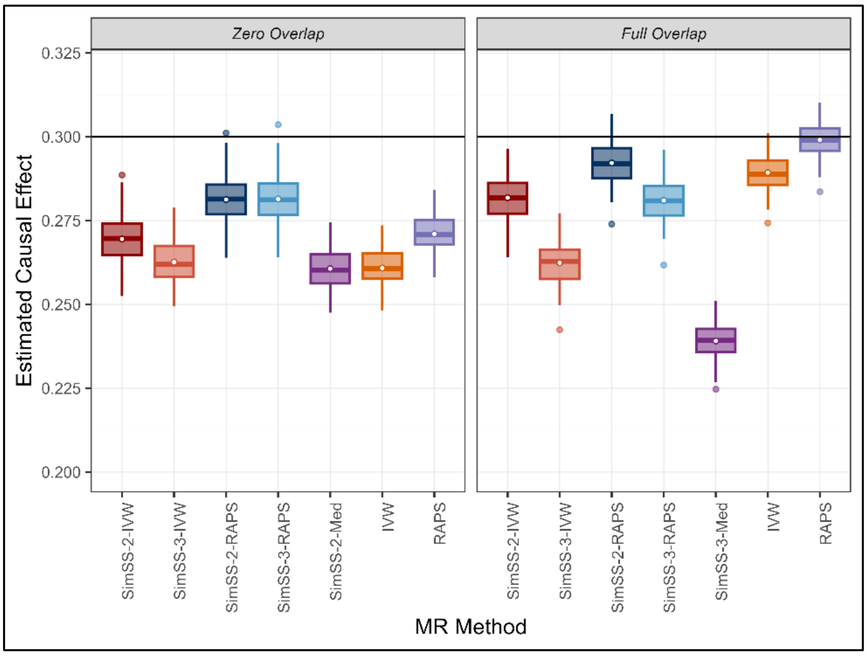

Fig O: Estimated causal effect for each method under a directional negatively-correlated pleiotropy setting ($\mu_\alpha = 0.2, \text{corr}(\alpha, \beta_X) = -0.2$) with $n_X = n_Y = 200{,}000$, heritability = 0.7, proportion of true effect variants = 0.01 and proportion of effect variants exhibiting pleiotropy = 0.2, across 100 simulated pairs of exposure and outcome GWAS summary statistics for each setting. SimSS-2-Med and SimSS-3-Med = 2-split and 3-split versions, respectively, of MR-SimSS using MR weighted median.

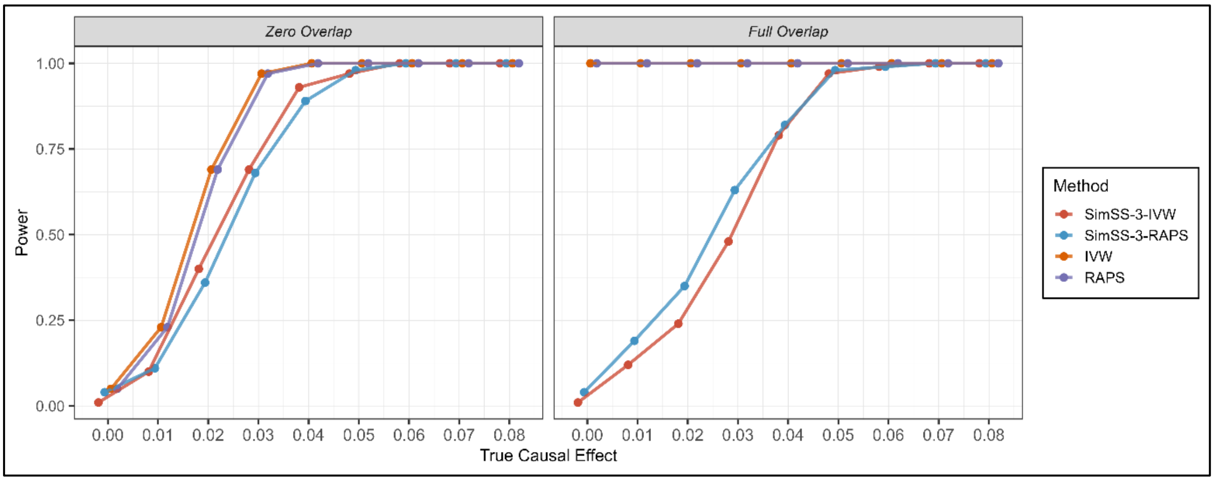

Fig P: Estimated power plotted against true causal effect, computed over 100 simulated pairs of exposure and outcome GWAS summary statistics, in zero and full sample overlap settings with $n_X = n_Y = 200{,}000$, heritability = 0.3, proportion of true effect variants = 0.01 and exposure-outcome correlation = 0.5.

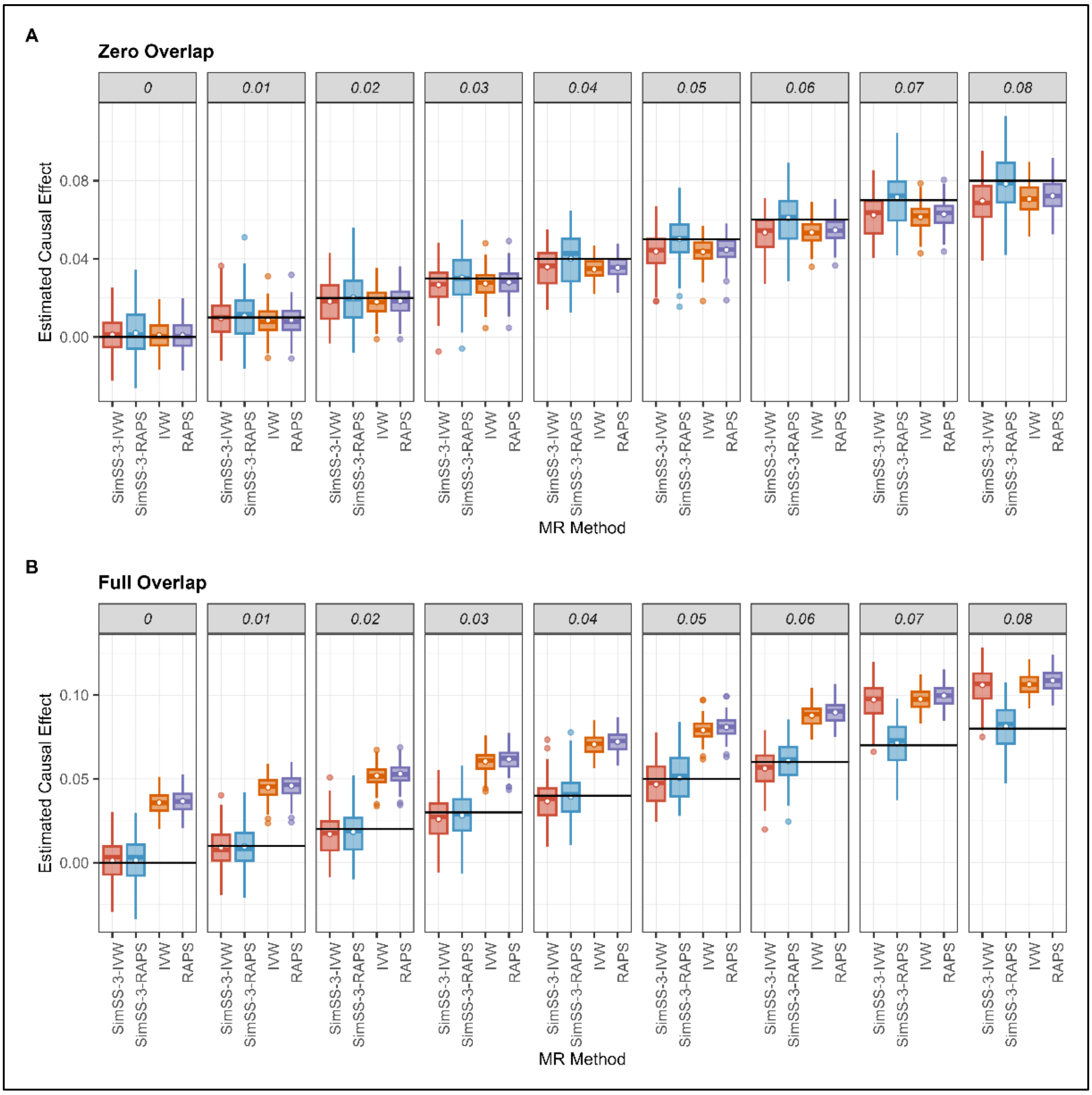

Fig Q: Estimated causal effect for each method for zero and full sample overlap with $n_X = n_Y = 200{,}000$, heritability = 0.3, proportion of true effect variants = 0.01 and exposure-outcome correlation = 0.5, across 100 simulated pairs of exposure and outcome GWAS summary statistics for each setting.

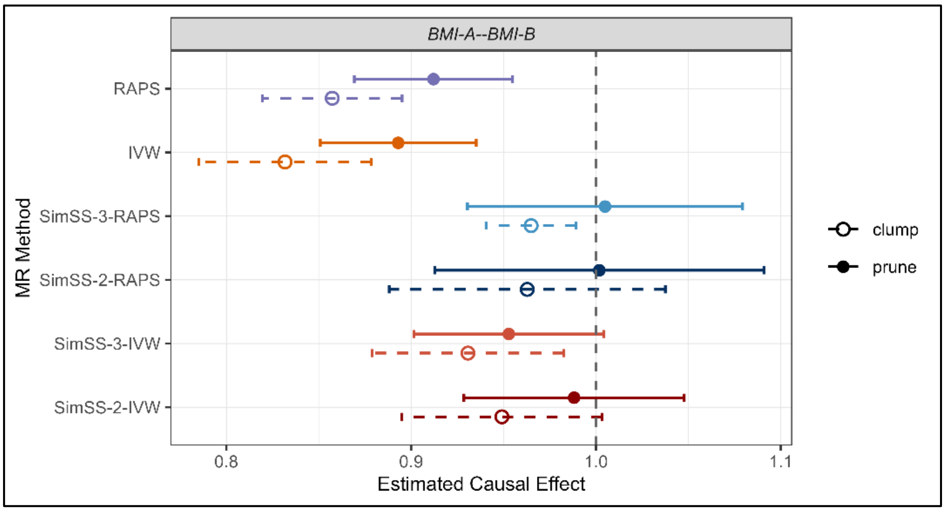

Fig R: Estimated causal effects for each method resulting from a same-trait BMI-BMI analysis using a set of pruned variants (LD pruning via PLINK 2.0 using command 'indep-pairwise 100 10 0.01') and a set of clumped variants (LD clumping via 'clump data()' function from 'twoSampleMR' R package using default parameters: 'clump kb' = 10,000, 'clump r2' = 0.001), with both sets providing a similar number of instruments.

Supplementary Tables

Table A Summarized simulation results for each method, averaged over all parameter combinations, including fraction of overlap, for sample sizes of 200,000.

Method	$\hat{\beta}$	bias	|bias|	RMSE	SE	CP	# IVs
**SimSS-2-IVW**	0.2959	-0.0041	0.0065	0.0094	0.0063	0.8589	367.26
**SimSS-3-IVW**	0.2879	-0.0121	0.0122	0.0156	0.0061	0.4929	367.26
**SimSS-2-RAPS**	0.3018	0.0018	0.0057	0.0083	0.0066	0.9296	367.25
**SimSS-3-RAPS**	0.3001	0.0001	0.0051	0.0075	0.0061	0.9390	367.29
**IVW**	0.2901	-0.0099	0.0120	0.0174	0.0045	0.5450	765.17
**RAPS**	0.2937	-0.0063	0.0099	0.0147	0.0046	0.6210	765.17

Table B Summarized simulation results for each method for sample sizes of 200,000, with zero overlap for different values of exposure-outcome correlation, averaged over heritability and proportion of true effect variants.

Method	$\hat{\beta}$	bias	|bias|	RMSE	SE	CP	# IVs
Cor(X,Y) = -0.1							
**SimSS-2-IVW**	0.2939	-0.0061	0.0074	0.0079	0.0063	0.8200	367.32
**SimSS-3-IVW**	0.2880	-0.0120	0.0122	0.0099	0.0063	0.5175	367.35
**SimSS-2-RAPS**	0.3001	0.0001	0.0047	0.007	0.0063	0.9650	367.34
**SimSS-3-RAPS**	0.3002	0.0002	0.0049	0.0072	0.0062	0.9525	367.35
**IVW**	0.2854	-0.0146	0.0151	0.0142	0.0045	0.4450	764.42
**RAPS**	0.2891	-0.0109	0.0199	0.0125	0.0046	0.4900	764.42
Cor(X,Y) = 0.1							
**SimSS-2-IVW**	0.2938	-0.0062	0.0072	0.0086	0.0063	0.8225	366.13
**SimSS-3-IVW**	0.2879	-0.0121	0.0122	0.0103	0.0064	0.5225	366.12
**SimSS-2-RAPS**	0.3001	0.0001	0.0051	0.0079	0.0063	0.9425	366.12
**SimSS-3-RAPS**	0.3001	0.0001	0.0052	0.0080	0.0063	0.9475	366.11
**IVW**	0.2852	-0.0148	0.0151	0.0142	0.0045	0.4225	764.92
**RAPS**	0.2889	-0.0111	0.0120	0.0125	0.0046	0.5100	764.92
Cor(X,Y) = 0.3							
**SimSS-2-IVW**	0.2933	-0.0067	0.0075	0.0082	0.0063	0.8275	368.14
**SimSS-3-IVW**	0.2876	-0.0124	0.0125	0.0102	0.0063	0.5150	368.14
**SimSS-2-RAPS**	0.2996	-0.0004	0.0049	0.0072	0.0063	0.9500	368.15
**SimSS-3-RAPS**	0.2997	-0.0003	0.0051	0.0073	0.0063	0.9500	368.16
**IVW**	0.285	-0.015	0.0153	0.0145	0.0045	0.4475	766.04
**RAPS**	0.2887	-0.0113	0.0122	0.0128	0.0046	0.4850	766.04

Table C Summarized simulation results for each method for sample sizes of 200,000, with complete overlap for different values of exposure-outcome correlation, averaged over heritability and proportion of true effect variants.

Method	$\hat{\beta}$	bias	|bias|	RMSE	SE	CP	# IVs
Cor(X,Y) = -0.1							
**SimSS-2-IVW**	0.2917	-0.0083	0.0091	0.0091	0.0065	0.7175	366.59
**SimSS-3-IVW**	0.2879	-0.0121	0.0124	0.0103	0.0065	0.525	366.59
**SimSS-2-RAPS**	0.2983	-0.0017	0.0056	0.0081	0.0060	0.9075	366.56
**SimSS-3-RAPS**	0.3000	0.0000	0.0055	0.0082	0.0065	0.93	366.56
**IVW**	0.2801	-0.0199	0.0201	0.0184	0.0046	0.3975	765.46
**RAPS**	0.2839	-0.0161	0.0167	0.0167	0.0046	0.4625	765.46
Cor(X,Y) = 0.1							
**SimSS-2-IVW**	0.2958	-0.0042	0.0060	0.0075	0.0062	0.895	368.46
**SimSS-3-IVW**	0.2879	-0.0121	0.0123	0.0098	0.0062	0.505	368.48
**SimSS-2-RAPS**	0.3018	0.0018	0.0052	0.0074	0.0066	0.9425	368.44
**SimSS-3-RAPS**	0.3000	0.0000	0.0051	0.0075	0.0061	0.9275	368.48
**IVW**	0.2901	-0.0099	0.0103	0.0097	0.0044	0.49	765.94
**RAPS**	0.2937	-0.0063	0.0073	0.0082	0.0046	0.7125	765.94
Cor(X,Y) = 0.3							
**SimSS-2-IVW**	0.3000	0.0000	0.0046	0.0068	0.0063	0.9675	366.62
**SimSS-3-IVW**	0.2879	-0.0121	0.0121	0.0096	0.0058	0.4875	366.61
**SimSS-2-RAPS**	0.3054	0.0054	0.0067	0.0077	0.0071	0.9175	366.63
**SimSS-3-RAPS**	0.3001	0.0001	0.0048	0.0072	0.0058	0.9425	366.61
**IVW**	0.2998	-0.0002	0.0033	0.0044	0.0043	0.965	763.77
**RAPS**	0.3031	0.0031	0.0043	0.0047	0.0046	0.9125	763.77

Table D Summarized simulation results for each method for sample sizes of 200,000, with 25% overlap for different values of exposure-outcome correlation, averaged over heritability and proportion of true effect variants.

Method	$\hat{\beta}$	bias	|bias|	RMSE	SE	CP	# IVs
Cor(X,Y) = -0.1							
**SimSS-2-IVW**	0.2933	-0.0067	0.0077	0.0104	0.0063	0.7875	367.60
**SimSS-3-IVW**	0.2880	-0.0120	0.0123	0.0154	0.0063	0.4650	367.62
**SimSS-2-RAPS**	0.2996	-0.0004	0.0051	0.0073	0.0063	0.9400	367.61
**SimSS-3-RAPS**	0.3001	0.0001	0.0051	0.0075	0.0063	0.9400	367.59
**IVW**	0.2837	-0.0163	0.0167	0.0222	0.0045	0.4150	766.38
**RAPS**	0.2874	-0.0126	0.0134	0.0184	0.0046	0.4600	766.38
Cor(X,Y) = 0.1							
**SimSS-2-IVW**	0.2945	-0.0055	0.0071	0.0010	0.0063	0.8275	367.84
**SimSS-3-IVW**	0.2881	-0.0119	0.0122	0.0157	0.0062	0.5400	367.87
**SimSS-2-RAPS**	0.3007	0.0007	0.0054	0.0081	0.0063	0.9500	367.84
**SimSS-3-RAPS**	0.3003	0.0003	0.0054	0.0082	0.0063	0.9475	367.87
**IVW**	0.2868	-0.0132	0.0137	0.0188	0.0045	0.4450	766.63
**RAPS**	0.2904	-0.0096	0.0105	0.0146	0.0046	0.5100	766.63
Cor(X,Y) = 0.3							
**SimSS-2-IVW**	0.2960	-0.0040	0.0057	0.0082	0.0062	0.9100	367.40
**SimSS-3-IVW**	0.2886	-0.0114	0.0116	0.0148	0.0062	0.5275	367.40
**SimSS-2-RAPS**	0.3020	0.0020	0.0050	0.0073	0.0065	0.9575	367.40
**SimSS-3-RAPS**	0.3008	0.0008	0.0049	0.0072	0.0062	0.9500	367.40
**IVW**	0.2891	-0.0109	0.0114	0.0157	0.0044	0.4925	766.18
**RAPS**	0.2927	-0.0073	0.0084	0.0122	0.0046	0.6600	766.18
Cor(X,Y) = 0.5							
**SimSS-2-IVW**	0.2975	-0.0025	0.0051	0.0074	0.0062	0.9400	366.93
**SimSS-3-IVW**	0.2890	-0.0110	0.0111	0.0143	0.0061	0.5375	366.93
**SimSS-2-RAPS**	0.3035	0.0035	0.0058	0.0083	0.0067	0.9375	366.93
**SimSS-3-RAPS**	0.3012	0.0012	0.0052	0.0075	0.0060	0.9300	366.92
**IVW**	0.2920	-0.0080	0.0087	0.0119	0.0044	0.5725	764.48
**RAPS**	0.2955	-0.0045	0.0060	0.0086	0.0046	0.7975	764.548

Table E Summarized simulation results for each method for sample sizes of 200,000, with 50% overlap for different values of exposure-outcome correlation, averaged over heritability and proportion of true effect variants.

Method	$\hat{\beta}$	bias	|bias|	RMSE	SE	CP	# IVs
Cor(X,Y) = -0.1							
**SimSS-2-IVW**	0.2925	-0.0075	0.0082	0.0111	0.0063	0.7175	366.43
**SimSS-3-IVW**	0.2877	-0.0123	0.0124	0.0156	0.0064	0.5100	366.43
**SimSS-2-RAPS**	0.2989	-0.0011	0.0053	0.0076	0.0062	0.9375	366.45
**SimSS-3-RAPS**	0.2998	-0.0002	0.0052	0.0075	0.0064	0.9525	366.43
**IVW**	0.2824	-0.0176	0.0178	0.0238	0.0046	0.4100	763.99
**RAPS**	0.2861	-0.0139	0.0144	0.0200	0.0046	0.4500	763.99
Cor(X,Y) = 0.1							
**SimSS-2-IVW**	0.2950	-0.0054	0.0066	0.0096	0.0062	0.8550	367.57
**SimSS-3-IVW**	0.2882	-0.0118	0.0120	0.0155	0.0062	0.4975	367.56
**SimSS-2-RAPS**	0.3011	0.0011	0.0056	0.0082	0.0064	0.9450	367.55
**SimSS-3-RAPS**	0.3003	0.0003	0.0056	0.0082	0.0062	0.9200	367.54
**IVW**	0.2876	-0.0124	0.0128	0.0173	0.0045	0.4525	766.35
**RAPS**	0.2912	-0.0088	0.0097	0.0136	0.0046	0.5675	766.35
Cor(X,Y) = 0.3							
**SimSS-2-IVW**	0.2965	-0.0035	0.0054	0.0078	0.0062	0.8975	367.48
**SimSS-3-IVW**	0.2874	-0.0126	0.0127	0.0160	0.0060	0.4775	367.47
**SimSS-2-RAPS**	0.3022	0.0022	0.0051	0.0071	0.0070	0.9650	367.44
**SimSS-3-RAPS**	0.2995	-0.0005	0.0048	0.0069	0.0061	0.9400	367.49
**IVW**	0.2926	-0.0074	0.0081	0.0011	0.0044	0.5950	764.61
**RAPS**	0.2961	-0.0039	0.0055	0.0079	0.0046	0.8525	764.61
Cor(X,Y) = 0.5							
**SimSS-2-IVW**	0.2988	-0.0012	0.0046	0.0068	0.0062	0.9574	367.87
**SimSS-3-IVW**	0.2878	-0.0122	0.0123	0.0154	0.0060	0.4625	367.85
**SimSS-2-RAPS**	0.3044	0.0044	0.0059	0.0085	0.0070	0.9250	367.88
**SimSS-3-RAPS**	0.3001	0.0001	0.0047	0.0070	0.0057	0.9250	367.92
**IVW**	0.2973	-0.0027	0.0043	0.0057	0.0043	0.9000	763.97
**RAPS**	0.3006	0.0006	0.0036	0.0047	0.0046	0.9550	763.97

Table F Summarized simulation results for each method for sample sizes of 200,000, with 75% overlap for different values of exposure-outcome correlation, averaged over heritability and proportion of true effect variants.

Method	$\widehat{\beta}$	bias	|bias|	RMSE	SE	CP	# IVs
Cor(X,Y) = -0.1							
**SimSS-2-IVW**	0.2918	-0.0082	0.0090	0.0121	0.0064	0.7050	366.21
**SimSS-3-IVW**	0.2873	-0.0127	0.0129	0.0164	0.0064	0.4800	366.16
**SimSS-2-RAPS**	0.2982	-0.0018	0.0055	0.0080	0.0061	0.9225	366.17
**SimSS-3-RAPS**	0.2995	-0.0005	0.0055	0.0079	0.0065	0.9275	366.18
**IVW**	0.2812	-0.0188	0.0190	0.0255	0.0046	0.3850	765.08
**RAPS**	0.2850	-0.0150	0.0156	0.0216	0.0046	0.4375	765.08
Cor(X,Y) = 0.1							
**SimSS-2-IVW**	0.2948	-0.0052	0.0066	0.0094	0.0062	0.8725	367.16
**SimSS-3-IVW**	0.2873	-0.0127	0.0128	0.0163	0.0062	0.4925	367.18
**SimSS-2-RAPS**	0.3008	0.0008	0.0051	0.0074	0.0065	0.9575	367.19
**SimSS-3-RAPS**	0.2995	-0.0005	0.0051	0.0075	0.0062	0.9550	367.18
**IVW**	0.2886	-0.0114	0.0119	0.0160	0.0044	0.4550	765.04
**RAPS**	0.2922	-0.0078	0.0089	0.0124	0.0046	0.6225	765.04
Cor(X,Y) = 0.3							
**SimSS-2-IVW**	0.2990	-0.0010	0.0048	0.0070	0.0062	0.9650	367.56
**SimSS-3-IVW**	0.2885	-0.0115	0.0118	0.0149	0.0059	0.4600	367.57
**SimSS-2-RAPS**	0.3045	0.0045	0.0060	0.0089	0.0069	0.9275	367.56
**SimSS-3-RAPS**	0.3007	0.0007	0.0049	0.0074	0.0059	0.9275	367.55
**IVW**	0.2966	-0.0034	0.0050	0.0068	0.0043	0.8525	764.35
**RAPS**	0.3000	-0.0000	0.0037	0.0051	0.0046	0.9375	764.35
Cor(X,Y) = 0.5							
**SimSS-2-IVW**	0.3018	0.0018	0.0047	0.0067	0.0065	0.9700	367.13
**SimSS-3-IVW**	0.2882	-0.0118	0.0120	0.0150	0.0058	0.4775	367.13
**SimSS-2-RAPS**	0.3070	0.0070	0.0076	0.0104	0.0074	0.8500	367.15
**SimSS-3-RAPS**	0.3004	0.0004	0.0046	0.0067	0.0056	0.9375	367.14
**IVW**	0.3038	0.0038	0.0050	0.0067	0.0043	0.8400	766.47
**RAPS**	0.3070	0.0070	0.0075	0.0097	0.0046	0.6600	766.47

Table G Summarized simulation results for each method with true causal effect = 0 and fully overlapping samples of size $n_X = n_Y = 200{,}000$, for different values of exposure-outcome correlation, averaged over heritability and proportion of true effect variants.

Method	bias ($\hat{\beta}$)	|bias|	RMSE	SE	CP	FPR	# IVs
Cor(X,Y) = -0.1							
**SimSS-2-IVW**	-0.0019	0.0050	0.0074	0.0062	0.9395	0.0088	367.51
**SimSS-3-IVW**	0.0001	0.0048	0.0070	0.0063	0.9568	0.0220	367.51
**SimSS-2-RAPS**	-0.0020	0.0052	0.0076	0.0060	0.9298	0.0122	367.52
**SimSS-3-RAPS**	0.0001	0.0051	0.0075	0.0060	0.9380	0.0305	367.51
**IVW**	-0.0049	0.0058	0.0080	0.0044	0.7810	0.2190	765.46
**RAPS**	-0.0049	0.0059	0.0081	0.0044	0.7778	0.2225	765.46
Cor(X,Y) = 0.1							
**SimSS-2-IVW**	0.0022	0.0052	0.0077	0.0062	0.9360	0.0558	367.52
**SimSS-3-IVW**	0.0001	0.0049	0.0072	0.0063	0.9560	0.0215	367.51
**SimSS-2-RAPS**	0.0023	0.0054	0.0080	0.0060	0.9228	0.0675	367.51
**SimSS-3-RAPS**	0.0002	0.0052	0.0078	0.0060	0.9338	0.0357	367.52
**IVW**	0.0050	0.0060	0.0081	0.0044	0.7768	0.2232	765.32
**RAPS**	0.0050	0.0061	0.0083	0.0044	0.7710	0.2290	765.32
Cor(X,Y) = 0.3							
**SimSS-2-IVW**	0.0061	0.0071	0.0098	0.0063	0.8130	0.1858	367.21
**SimSS-3-IVW**	-0.0001	0.0047	0.0069	0.0064	0.9595	0.0215	367.22
**SimSS-2-RAPS**	0.0063	0.0073	0.0102	0.0061	0.7958	0.2030	367.21
**SimSS-3-RAPS**	-0.0001	0.0050	0.0073	0.0061	0.9400	0.0302	367.21
**IVW**	0.0148	0.0151	0.0203	0.0044	0.4202	0.5798	764.55
**RAPS**	0.0151	0.0154	0.0208	0.0044	0.4180	0.5820	764.55
Cor(X,Y) = 0.5							
**SimSS-2-IVW**	0.0103	0.0106	0.0138	0.0064	0.6008	0.3990	367.09
**SimSS-3-IVW**	0.0000	0.0047	0.0070	0.0065	0.9645	0.0192	367.08
**SimSS-2-RAPS**	0.0106	0.0109	0.0142	0.0063	0.5758	0.4240	367.08
**SimSS-3-RAPS**	0.0000	0.0050	0.0075	0.0062	0.9478	0.0300	368.09
**IVW**	0.0249	0.0250	0.0337	0.0044	0.3318	0.6682	764.02
**RAPS**	0.0254	0.0255	0.0344	0.0044	0.3282	0.6718	764.02

Table H Summarized simulation results for each method, averaged over all parameter combinations, including fraction of overlap, for sample sizes of 500,000.

Method	$\hat{\beta}$	bias	|bias|	RMSE	SE	CP	# IVs
**SimSS-2-IVW**	0.2974	-0.0026	0.0036	0.0048	0.0027	0.7666	940.47
**SimSS-3-IVW**	0.2924	-0.0076	0.0077	0.0094	0.0026	0.3660	940.47
**SimSS-2-RAPS**	0.3011	0.0011	0.0027	0.0037	0.0028	0.9011	940.48
**SimSS-3-RAPS**	0.3000	0.0000	0.0021	0.0029	0.0027	0.9406	940.48
**IVW**	0.2956	-0.0044	0.0056	0.0080	0.0024	0.5762	1602.74
**RAPS**	0.2979	-0.0021	0.0042	0.0060	0.0024	0.6840	1602.74

Table I Summarized simulation results for each method, averaged over all parameter combinations, including fraction of overlap, for sample sizes of 50,000.

Method	$\widehat{\beta}$	bias	|bias|	RMSE	SE	CP	# IVs
**SimSS-2-IVW**	0.2964	-0.0036	0.0317	0.0586	0.0569	0.9496	71.90
**SimSS-3-IVW**	0.2797	-0.0203	0.0379	0.0668	0.0733	0.8618	72.40
**SimSS-2-RAPS**	0.3160	0.0160	0.0385	0.1376	0.1876	0.9600	71.43
**SimSS-3-RAPS**	0.3012	0.0012	0.0848	0.6539	58.2921	0.9582	72.40
**IVW**	0.2693	-0.0307	0.0379	0.0582	0.0227	0.6284	163.17
**RAPS**	0.2741	-0.0259	0.0353	0.0562	0.0224	0.6932	163.07

Table J Summarized simulation results for SimSS-3-RAPS using four different significance thresholds, averaged over varying fractions of overlap, with sample sizes of 50,000, heritability = 0.3, proportion of true effect variants = 0.01 and exposure-outcome correlation = 0.5.

Threshold	$\widehat{\beta}$	bias	|bias|	RMSE	SE	CP	# IVs
**5×10^{-8}**	0.3105	0.0105	0.2518	0.9231	0.9613	0.924	5.67
**5×10^{-6}**	0.3516	0.0516	0.1502	0.5512	0.6687	0.698	13.13
**5×10^{-5}**	0.3155	0.0155	0.0408	0.0713	0.0700	0.992	76.53
**5×10^{-4}**	0.3067	0.0067	0.0258	0.0318	0.0313	0.986	590.43

Table K Summarized simulation results for each method under a balanced pleiotropy setting ($\mu_\alpha = 0$) with $n_X = n_Y = 200{,}000$, heritability = 0.7, proportion of true effect variants = 0.01 and proportion of effect variants exhibiting pleiotropy = 0.2, across 100 simulated pairs of exposure and outcome GWAS summary statistics for each setting.

Method	$\widehat{\beta}$	bias	|bias|	RMSE	SE	CP	# IVs
Zero Overlap							
**SimSS-2-IVW**	0.2925	-0.0075	0.0085	0.0102	0.0066	0.80	591.25
**SimSS-3-IVW**	0.2854	-0.0146	0.0148	0.0162	0.0065	0.39	591.31
**SimSS-2-RAPS**	0.3048	0.0048	0.0070	0.0086	0.0038	0.60	591.26
**SimSS-3-RAPS**	0.3050	0.0050	0.0072	0.0089	0.0034	0.50	591.24
**IVW**	0.2830	-0.0170	0.0170	0.0179	0.0050	0.07	1549.90
**RAPS**	0.2936	-0.0064	0.0069	0.0085	0.0035	0.54	1549.90
Full Overlap							
**SimSS-2-IVW**	0.3047	0.0047	0.0062	0.0077	0.0062	0.87	589.49
**SimSS-3-IVW**	0.2848	-0.0152	0.0152	0.0163	0.0060	0.28	589.46
**SimSS-2-RAPS**	0.3153	0.0153	0.0154	0.0165	0.0050	0.21	589.35
**SimSS-3-RAPS**	0.3043	0.0043	0.0060	0.0075	0.0025	0.43	589.22
**IVW**	0.3114	0.0114	0.0115	0.0125	0.0047	0.32	1553.99
**RAPS**	0.3214	0.0214	0.0214	0.0220	0.0035	0.01	1553.99

Table L Summarized simulation results for each method under a directional pleiotropy setting ($\mu_\alpha = 0.2$) with $n_X = n_Y = 200{,}000$, heritability = 0.7, proportion of true effect variants = 0.01 and proportion of effect variants exhibiting pleiotropy = 0.2, across 100 simulated pairs of exposure and outcome GWAS summary statistics for each setting.

Method	$\hat{\beta}$	bias	|bias|	RMSE	SE	CP	# IVs
Zero Overlap							
**SimSS-2-IVW**	0.2925	-0.0075	0.0086	0.0102	0.0066	0.81	591.25
**SimSS-3-IVW**	0.2850	-0.0150	0.0150	0.0162	0.0065	0.38	591.31
**SimSS-2-RAPS**	0.3048	0.0048	0.0070	0.0087	0.0038	0.59	591.26
**SimSS-3-RAPS**	0.3049	0.0049	0.0071	0.0089	0.0034	0.50	591.24
**IVW**	0.2830	-0.0170	0.0170	0.0179	0.0050	0.08	1549.90
**RAPS**	0.2936	-0.0064	0.0070	0.0085	0.0035	0.50	1549.90
Full Overlap							
**SimSS-2-IVW**	0.3049	0.0048	0.0062	0.0078	0.0062	0.85	589.49
**SimSS-3-IVW**	0.2848	-0.0152	0.0152	0.0165	0.0060	0.31	589.46
**SimSS-2-RAPS**	0.3154	0.0154	0.0155	0.0166	0.0049	0.18	589.35
**SimSS-3-RAPS**	0.3047	0.0047	0.0063	0.0077	0.0025	0.43	589.53
**IVW**	0.3115	0.0115	0.0116	0.0126	0.0047	0.32	1553.99
**RAPS**	0.3215	0.0215	0.0215	0.0222	0.0035	0.01	1553.99

Table M Summarized simulation results for each method under a directional positively-correlated pleiotropy setting ($\mu_\alpha = 0.2, \mathrm{corr}(\alpha, \beta_X) = 0.2$) with $n_X = n_Y = 200{,}000$, heritability = 0.7, proportion of true effect variants = 0.01 and proportion of effect variants exhibiting pleiotropy = 0.2, across 100 simulated pairs of exposure and outcome GWAS summary statistics for each setting.

Method	$\hat{\beta}$	bias	|bias|	RMSE	SE	CP	# IVs
Zero Overlap							
**SimSS-2-IVW**	0.3156	0.0156	0.0156	0.0171	0.0068	0.36	591.25
**SimSS-3-IVW**	0.3073	0.0073	0.0079	0.0096	0.0067	0.82	591.31
**SimSS-2-RAPS**	0.3292	0.0292	0.0292	0.0302	0.0037	0.00	591.26
**SimSS-3-RAPS**	0.3293	0.0293	0.0293	0.0303	0.0032	0.00	591.24
**SimSS-2-Med**	0.2827	-0.0173	0.0173	0.0182	0.0073	0.33	591.19
**IVW**	0.3052	0.0052	0.0065	0.0076	0.0051	0.82	1549.90
**RAPS**	0.3169	0.0169	0.0169	0.0179	0.0035	0.06	1549.90
Full Overlap							
**SimSS-2-IVW**	0.3279	0.0279	0.0279	0.0286	0.0063	0.01	589.49
**SimSS-3-IVW**	0.3072	0.0072	0.0081	0.0096	0.0062	0.79	589.46
**SimSS-2-RAPS**	0.3395	0.0395	0.0395	0.0400	0.0049	0.00	589.35
**SimSS-3-RAPS**	0.3290	0.0290	0.0290	0.0286	0.0022	0.00	589.36
**SimSS-3-Med**	0.2646	-0.0354	0.0354	0.0357	0.0086	0.00	589.80
**IVW**	0.3338	0.0338	0.0338	0.0342	0.0048	0.00	1553.99
**RAPS**	0.3447	0.0447	0.0447	0.0450	0.0035	0.00	1553.99

Table N Outline of method-specific biases under a directional, positively-correlated pleiotropy setting in which 20% of true effect variants exhibit pleiotropic effects. Arrows indicate the direction of bias, while labels 1, 2, and 3 denote weak instrument bias arising in the full-sample, two-split, and three-split settings, respectively, with greater splitting leading to increased weak instrument bias.

Method	Winner's Curse bias	Weak instrument bias	Bias due to correlated pleiotropy
Zero Overlap			
**SimSS-2-IVW**	✗	✓ ($\downarrow^2$)	✓ (↑)
**SimSS-3-IVW**	✗	✓ ($\downarrow^3$)	✓ (↑)
**SimSS-2-RAPS**	✗	✗	✓ (↑)
**SimSS-3-RAPS**	✗	✗	✓ (↑)
**SimSS-2-Med**	✗	✓ ($\downarrow^2$)	✗
**IVW**	✓ (↓)	✓ ($\downarrow^1$)	✓ (↑)
**RAPS**	✓ (↓)	✗	✓ (↑)
Full Overlap			
**SimSS-2-IVW**	✗	✓ ($\uparrow^2$)	✓ (↑)
**SimSS-3-IVW**	✗	✓ ($\downarrow^3$)	✓ (↑)
**SimSS-2-RAPS**	✗	✓ ($\uparrow^2$)	✓ (↑)
**SimSS-3-RAPS**	✗	✗	✓ (↑)
**SimSS-3-Med**	✗	✓ ($\downarrow^3$)	✗
**IVW**	✓ (↓)	✓ ($\uparrow^1$)	✓ (↑)
**RAPS**	✓ (↓)	✓ ($\uparrow^1$)	✓ (↑)

Table O Summarized simulation results for each method under a directional negatively-correlated pleiotropy setting ($\mu_\alpha = 0.2, \mathrm{corr}(\alpha, \beta_X) = -0.2$) with $n_X = n_Y = 200{,}000$, heritability = 0.7, proportion of true effect variants = 0.01 and proportion of effect variants exhibiting pleiotropy = 0.2, across 100 simulated pairs of exposure and outcome GWAS summary statistics for each setting.

Method	$\hat{\beta}$	bias	|bias|	RMSE	SE	CP	# IVs
Zero Overlap							
**SimSS-2-IVW**	0.2695	-0.0305	0.0305	0.0313	0.0068	0.01	591.25
**SimSS-3-IVW**	0.2626	-0.0374	0.0374	0.0379	0.0066	0.00	591.31
**SimSS-2-RAPS**	0.2813	-0.0187	0.0188	0.0200	0.0038	0.08	591.26
**SimSS-3-RAPS**	0.2814	-0.0186	0.0186	0.0200	0.0034	0.07	591.24
**SimSS-2-Med**	0.2607	-0.0393	0.0393	0.0398	0.0072	0.00	591.19
**IVW**	0.2608	-0.0392	0.0392	0.0395	0.0051	0.00	1549.90
**RAPS**	0.2710	-0.0290	0.0290	0.0295	0.0035	0.00	1549.90
Full Overlap							
**SimSS-2-IVW**	0.2818	-0.0182	0.0182	0.0192	0.0064	0.15	589.49
**SimSS-3-IVW**	0.2624	-0.0376	0.0376	0.0381	0.0062	0.00	589.46
**SimSS-2-RAPS**	0.2922	-0.0078	0.0084	0.0100	0.0049	0.60	589.35
**SimSS-3-RAPS**	0.2811	-0.0190	0.0190	0.0197	0.0025	0.02	589.36
**SimSS-3-Med**	0.2391	-0.0609	0.0609	0.0611	0.0085	0.00	589.80
**IVW**	0.2893	-0.0107	0.0107	0.0119	0.0048	0.43	1553.99
**RAPS**	0.2991	-0.0009	0.0043	0.0053	0.0035	0.81	1553.99

Table P Outline of method-specific biases under a directional, negatively-correlated pleiotropy setting in which 20% of true effect variants exhibit pleiotropic effects. Arrows indicate the direction of bias, while labels 1, 2, and 3 denote weak instrument bias arising in the full-sample, two-split, and three-split settings, respectively, with greater splitting leading to increased weak instrument bias.

Method	Winner's Curse bias	Weak instrument bias	Bias due to correlated pleiotropy
Zero Overlap			
**SimSS-2-IVW**	✗	✓ ($\downarrow^2$)	✓ (↓)
**SimSS-3-IVW**	✗	✓ ($\downarrow^3$)	✓ (↓)
**SimSS-2-RAPS**	✗	✗	✓ (↓)
**SimSS-3-RAPS**	✗	✗	✓ (↓)
**SimSS-2-Med**	✗	✓ ($\downarrow^2$)	✗
**IVW**	✓ (↓)	✓ ($\downarrow^1$)	✓ (↓)
**RAPS**	✓ (↓)	✗	✓ (↓)
Full Overlap			
**SimSS-2-IVW**	✗	✓ ($\uparrow^2$)	✓ (↓)
**SimSS-3-IVW**	✗	✓ ($\downarrow^3$)	✓ (↓)
**SimSS-2-RAPS**	✗	✓ ($\uparrow^2$)	✓ (↓)
**SimSS-3-RAPS**	✗	✗	✓ (↓)
**SimSS-3-Med**	✗	✓ ($\downarrow^3$)	✗
**IVW**	✓ (↓)	✓ ($\uparrow^1$)	✓ (↓)
**RAPS**	✓ (↓)	✓ ($\uparrow^1$)	✓ (↑)

Table Q Summarized results for each method resulting from a same-trait BMI-BMI analysis using a set of pruned variants (LD pruning via PLINK 2.0 using command 'indep-pairwise 100 10 0.01') and a set of clumped variants (LD clumping via 'clump data()' function from 'twoSampleMR' R package using default parameters: 'clump kb' = 10,000, 'clump r2' = 0.001), with both sets providing a similar number of instruments.

Method	$\hat{\beta}$	SE	95% CI	# IVs
Pruned variants				
**SimSS-2-IVW**	0.9881	0.0304	[0.929, 1.048]	15.09
**SimSS-3-IVW**	0.9528	0.0262	[0.902, 1.004]	15.29
**SimSS-2-RAPS**	1.0018	0.0454	[0.913, 1.091]	15.06
**SimSS-3-RAPS**	1.0049	0.0380	[0.931, 1.079]	15.17
**IVW**	0.8930	0.0215	[0.851, 0.935]	83
**RAPS**	0.9121	0.0219	[0.869, 0.955]	83
Clumped variants				
**SimSS-2-IVW**	0.9491	0.0276	[0.895, 1.003]	21.37
**SimSS-3-IVW**	0.9307	0.0264	[0.879, 0.982]	21.40
**SimSS-2-RAPS**	0.9628	0.0381	[0.888, 1.038]	21.48
**SimSS-3-RAPS**	0.9649	0.0125	[0.940, 0.989]	21.48
**IVW**	0.8317	0.0239	[0.785, 0.878]	91
**RAPS**	0.8573	0.0193	[0.819, 0.895]	91

Derivation of conditional distribution

Let $\hat{\beta}_X$ and $\hat{\beta}_Y$ denote the estimated effects of a given genetic variant on the exposure and outcome, respectively, derived from the full sample. These are estimators of the true effects β_X and β_Y. Our simulated sample splitting method assumes that the full dataset of genotype, exposure and outcome information, $\{\boldsymbol{G}, \boldsymbol{X}, \boldsymbol{Y}\}$, is randomly partitioned into two disjoint subsets in proportions π_1 and $1 - \pi_1$. For a given variant, let $\hat{\beta}_X^1$ and $\hat{\beta}_Y^1$ denote the variant-exposure and variant-outcome association estimates in the first (π_1) subset. The goal is to derive the conditional distribution of the vector $\begin{bmatrix}\hat{\beta}_X^1\\ \hat{\beta}_Y^1\end{bmatrix}$ given the full-sample estimates $\begin{bmatrix}\hat{\beta}_X\\ \hat{\beta}_Y\end{bmatrix}$, presented in Equation (1) in the main manuscript. This derivation facilitates the generation of new, independent estimates as if obtained from separate subsamples, enabling us to mimic sample splitting using only summary-level data.

$$\Sigma_\mathrm{A} = \begin{bmatrix} \mathrm{var}(\hat{\beta}_X) & \mathrm{cov}(\hat{\beta}_X, \hat{\beta}_Y) \\ \mathrm{cov}(\hat{\beta}_X, \hat{\beta}_Y) & \mathrm{var}(\hat{\beta}_Y) \end{bmatrix}$$

Marginal distribution of $(\hat{\boldsymbol{\beta}}_X, \hat{\boldsymbol{\beta}}_Y)$

We begin by noting that the vector $\mathbf{A} = \begin{bmatrix}\hat{\beta}_X\\ \hat{\beta}_Y\end{bmatrix}$ has an asymptotic bivariate normal distribution with mean vector $\boldsymbol{\mu}_\mathrm{A}$ and covariance matrix Σ_A, i.e. $\mathbf{A} \sim N(\boldsymbol{\mu}_\mathrm{A}, \Sigma_\mathrm{A})$, where:

$$\Sigma_\mathrm{A} = \begin{bmatrix} \mathrm{var}(\hat{\beta}_X) & \mathrm{cov}(\hat{\beta}_X, \hat{\beta}_Y) \\ \mathrm{cov}(\hat{\beta}_X, \hat{\beta}_Y) & \mathrm{var}(\hat{\beta}_Y) \end{bmatrix} \quad \text{(S1)}$$

Expressions for this mean vector $\boldsymbol{\mu}_\mathrm{A}$ and covariance matrix Σ_A of the full-sample estimates $\hat{\beta}_X$ and $\hat{\beta}_Y$ are derived in what follows.

For a given variant, assume genotype data $\{G_i\}_{i=1}^n$, with $G_i \in \{0,1,2\}$ are available for each individual $i = 1, \dots, n$. Exposure data are observed for n_X individuals, outcome data for n_Y, and n_overlap individuals have both, with $n_\mathrm{overlap} \leq \min(n_X, n_Y)$.

Let $\boldsymbol{G_X} \in \mathbb{R}^{n_X \times 2}$ denote the design matrix for the exposure regression, where $\boldsymbol{G}_\mathbf{overlap}$ are the rows of the exposure matrix that are in common with the design matrix for the outcome regression:

$$\boldsymbol{G_X} = \begin{bmatrix} 1 & G_1 \\ \vdots & \vdots \\ 1 & G_{n_X} \end{bmatrix} = \begin{bmatrix} \boldsymbol{G}_\mathbf{overlap} \\ \boldsymbol{G}_{\boldsymbol{X}}^* \end{bmatrix} \quad \text{(S2)}$$

We assume the linear model $\boldsymbol{X} = \boldsymbol{G_X}\boldsymbol{\beta_X} + \boldsymbol{\varepsilon}$, where $\boldsymbol{\beta_X} = \begin{bmatrix}\beta_{X_0}\\ \beta_X\end{bmatrix}$, and $\hat{\boldsymbol{\beta}}_{\boldsymbol{X}} = (\boldsymbol{G}_{\boldsymbol{X}}^\mathrm{T}\boldsymbol{G_X})^{-1}\boldsymbol{G}_{\boldsymbol{X}}^\mathrm{T}\boldsymbol{X}$ is the least squares estimator. The vector $\hat{\boldsymbol{\beta}}_{\boldsymbol{X}} = \begin{bmatrix}\hat{\beta}_{X_0}\\ \hat{\beta}_X\end{bmatrix}$ therefore represents the intercept and slope

coefficients from this linear regression of the exposure, $\boldsymbol{X}$ on the genotype, $\boldsymbol{G}$, in which the slope coefficient, $\hat{\beta}_X$ provides an estimate for the variant-exposure association. From standard asymptotic theory (assuming the linear model is correct, observations are independent and individual variants explain only a small fraction of the variation in $\boldsymbol{X}$), $\hat{\boldsymbol{\beta}}_{\boldsymbol{X}} \sim N(\boldsymbol{\beta}_{\boldsymbol{X}}, \mathrm{var}(X)(\boldsymbol{G}_{\boldsymbol{X}}^{\mathrm{T}}\boldsymbol{G}_{\boldsymbol{X}})^{-\mathbf{1}})$. Therefore, $\mathbb{E}[\hat{\beta}_X] = \beta_X$, and $\mathrm{var}(\hat{\beta}_X)$ is given by the lower-right entry of the covariance matrix.

The matrix $\boldsymbol{G}_{\boldsymbol{X}}^{\mathrm{T}}\boldsymbol{G}_{\boldsymbol{X}}$ has the following structure:

$$\boldsymbol{G}_{\boldsymbol{X}}^{\mathrm{T}}\boldsymbol{G}_{\boldsymbol{X}} = \begin{bmatrix} n_X & \sum_{i=1}^{n_X} G_i \\ \sum_{i=1}^{n_X} G_i & \sum_{i=1}^{n_X} G_i^2 \end{bmatrix} \tag{S3}$$

Assuming Hardy-Weinberg equilibrium and defining the minor allele frequency maf < 0.5, we use $\mathbb{E}[G_i] = 2(\mathrm{maf})$ and $\mathbb{E}[G_i^2] = 4(\mathrm{maf})^2 + 2(\mathrm{maf})(1-\mathrm{maf})$. Applying the law of large numbers, we note:

$$\mathbb{E}[\boldsymbol{G}_{\boldsymbol{X}}^{\mathrm{T}}\boldsymbol{G}_{\boldsymbol{X}}] \approx \boldsymbol{G}_{\boldsymbol{X}}^{\mathrm{T}}\boldsymbol{G}_{\boldsymbol{X}} \approx \begin{bmatrix} n_X & 2n_X(\mathrm{maf}) \\ 2n_X(\mathrm{maf}) & n_X[4(\mathrm{maf})^2 + 2(\mathrm{maf})(1-\mathrm{maf})] \end{bmatrix} \tag{S4}$$

Letting $a = n_X$, $b = c = 2n_X(\mathrm{maf})$ and $d = n_X[4(\mathrm{maf})^2 + 2(\mathrm{maf})(1-\mathrm{maf})]$, the inverse matrix yields:

$$\mathrm{var}(\hat{\beta}_X) \approx \mathrm{var}(X) \cdot \frac{a}{ad - bc} = \frac{\mathrm{var}(X)}{n_X 2(\mathrm{maf})(1-\mathrm{maf})} \tag{S5}$$

Thus, under standard assumptions, $\boldsymbol{\mu}_{\mathbf{A}} = \begin{bmatrix} \mathbb{E}[\hat{\beta}_X] \\ \mathbb{E}[\hat{\beta}_Y] \end{bmatrix} = \begin{bmatrix} \beta_X \\ \beta_Y \end{bmatrix}$, and the (1,1) entry of $\sum_{\mathbf{A}}$ is given by the expression above. A symmetric derivation with respect to the outcome yields:

$$\mathrm{var}(\hat{\beta}_Y) \approx \frac{\mathrm{var}(Y)}{n_Y 2(\mathrm{maf})(1-\mathrm{maf})} \tag{S6}$$

comprising the (2,2) entry of $\sum_{\mathbf{A}}$.

Next, we derive an expression for $\mathrm{cov}(\hat{\beta}_X,\hat{\beta}_Y)$, the covariance between the exposure and outcome regression coefficients for a given variant. As illustrated above, these estimated coefficients have arisen from $\hat{\boldsymbol{\beta}}_{\boldsymbol{X}} = (\boldsymbol{G}_{\boldsymbol{X}}^{\mathrm{T}}\boldsymbol{G}_{\boldsymbol{X}})^{-\mathbf{1}}\boldsymbol{G}_{\boldsymbol{X}}^{\mathrm{T}}\boldsymbol{X}$ and $\hat{\boldsymbol{\beta}}_{\boldsymbol{Y}} = (\boldsymbol{G}_{\boldsymbol{Y}}^{\mathrm{T}}\boldsymbol{G}_{\boldsymbol{Y}})^{-\mathbf{1}}\boldsymbol{G}_{\boldsymbol{Y}}^{\mathrm{T}}\boldsymbol{Y}$. Under Hardy-Weinberg equilibrium and assuming large sample sizes, we can approximate:

$$(\boldsymbol{G}_{\boldsymbol{X}}^{\mathrm{T}}\boldsymbol{G}_{\boldsymbol{X}})^{-\mathbf{1}} \approx \frac{1}{n_X}\begin{bmatrix} 1 & 2(\mathrm{maf}) \\ 2(\mathrm{maf}) & [4(\mathrm{maf})^2 + 2(\mathrm{maf})(1-\mathrm{maf})] \end{bmatrix}^{-1}$$

$$(\boldsymbol{G}_{\boldsymbol{Y}}^{\mathrm{T}}\boldsymbol{G}_{\boldsymbol{Y}})^{-\mathbf{1}} \approx \frac{1}{n_Y}\begin{bmatrix} 1 & 2(\mathrm{maf}) \\ 2(\mathrm{maf}) & [4(\mathrm{maf})^2 + 2(\mathrm{maf})(1-\mathrm{maf})] \end{bmatrix}^{-1} \tag{S7}$$

Letting $\boldsymbol{C} = n_X\left(\mathbb{E}[\boldsymbol{G}_{\boldsymbol{X}}^{\mathrm{T}}\boldsymbol{G}_{\boldsymbol{X}}]\right)^{-\mathbf{1}} = n_Y\left(\mathbb{E}[\boldsymbol{G}_{\boldsymbol{Y}}^{\mathrm{T}}\boldsymbol{G}_{\boldsymbol{Y}}]\right)^{-\mathbf{1}}$ allows us to write:

$$\mathbf{cov}(\hat{\boldsymbol{\beta}}_{\boldsymbol{X}}, \hat{\boldsymbol{\beta}}_{\boldsymbol{Y}}) \approx \frac{1}{n_X n_Y}\mathbf{cov}(\boldsymbol{C}\boldsymbol{G}_{\mathbf{overlap}}^{\mathrm{T}}\boldsymbol{X}_{\mathbf{overlap}}, \boldsymbol{C}\boldsymbol{G}_{\mathbf{overlap}}^{\mathrm{T}}\boldsymbol{Y}_{\mathbf{overlap}}) \tag{S8}$$

in which $\boldsymbol{X}_{\mathbf{overlap}}$ and $\boldsymbol{Y}_{\mathbf{overlap}}$ are the elements of the $\boldsymbol{X}$ and $\boldsymbol{Y}$ vectors that correspond to the same individuals. Using the independence structure and $\mathbf{cov}(\boldsymbol{X}_{\mathbf{overlap}}, \boldsymbol{Y}_{\mathbf{overlap}}) = \mathrm{cov}(X, Y) \cdot \mathbb{I}_{n_{\mathrm{overlap}}}$, we obtain:

$$\mathbf{cov}(\widehat{\boldsymbol{\beta}}_X, \widehat{\boldsymbol{\beta}}_Y) \approx \frac{n_{\mathrm{overlap}} \mathrm{cov}(X, Y)}{n_X n_Y} \boldsymbol{C} \quad \text{(S9)}$$

We are specifically interested in $\mathrm{cov}(\hat{\beta}_X, \hat{\beta}_Y)$, the lower right element of the matrix. Since the corresponding entry of $\boldsymbol{C}$ is $\frac{1}{2(\mathrm{maf})(1-\mathrm{maf})}$, and using the identity $\mathrm{cov}(X, Y) = \rho\sqrt{\mathrm{var}(X)\mathrm{var}(Y)}$, where ρ is the exposure-outcome correlation, we obtain:

$$\mathrm{cov}(\hat{\beta}_X, \hat{\beta}_Y) \approx \frac{1}{2(\mathrm{maf})(1-\mathrm{maf})} \cdot \lambda \sqrt{\frac{\mathrm{var}(X)\mathrm{var}(Y)}{n_X n_Y}} \quad \text{(S10)}$$

in which λ is defined as $\lambda = \frac{n_{\mathrm{overlap}}\rho}{\sqrt{n_X n_Y}}$. Combining results from above, we arrive at a complete expression for $\Sigma_{\mathbf{A}}$:

$$\Sigma_{\mathrm{A}} = \frac{1}{2(\mathrm{maf})(1-\mathrm{maf})} \begin{bmatrix} \frac{\mathrm{var}(X)}{n_X} & \lambda \sqrt{\frac{\mathrm{var}(X)\mathrm{var}(Y)}{n_X n_Y}} \\ \lambda \sqrt{\frac{\mathrm{var}(X)\mathrm{var}(Y)}{n_X n_Y}} & \frac{\mathrm{var}(Y)}{n_Y} \end{bmatrix} \quad \text{(S11)}$$

Using standard error estimates:

$$\sigma_X^2 \approx \mathrm{var}(\hat{\beta}_X) \approx \frac{\mathrm{var}(X)}{n_X 2(\mathrm{maf})(1-\mathrm{maf})}, \quad \sigma_Y^2 \approx \mathrm{var}(\hat{\beta}_Y) \approx \frac{\mathrm{var}(Y)}{n_Y 2(\mathrm{maf})(1-\mathrm{maf})} \quad \text{(S12)}$$

Σ_{A} can be re-expressed simply as:

$$\Sigma_{\mathrm{A}} = \begin{bmatrix} \sigma_X^2 & \lambda\sigma_X\sigma_Y \\ \lambda\sigma_X\sigma_Y & \sigma_X^2 \end{bmatrix} \quad \text{(S13)}$$

Conditional distribution of $(\widehat{\boldsymbol{\beta}}_X^1, \widehat{\boldsymbol{\beta}}_Y^1)$

Similarly, let $\mathbf{B} = \begin{bmatrix} \hat{\beta}_X^1 \\ \hat{\beta}_Y^1 \end{bmatrix}$ represent the estimates from the π_1-split of the sample. We assume $\mathbf{B} \sim N(\boldsymbol{\mu}_{\mathrm{B}}, \Sigma_{\mathrm{B}})$ where:

$$\Sigma_{\mathrm{B}} = \begin{bmatrix} \mathrm{var}(\hat{\beta}_X^{\pi}) & \mathrm{cov}(\hat{\beta}_X^{\pi}, \hat{\beta}_Y^{\pi}) \\ \mathrm{cov}(\hat{\beta}_X^{\pi}, \hat{\beta}_Y^{\pi}) & \mathrm{var}(\hat{\beta}_Y^{\pi}) \end{bmatrix} \quad \text{(S14)}$$

Let $\Sigma_{\mathbf{AB}}$ and $\Sigma_{\mathbf{BA}}$ denote the cross-covariance matrices between $\mathbf{A}$ and $\mathbf{B}$. Then, by standard results on conditional multivariate normal distributions, the distribution of $\mathbf{B}|\mathbf{A} = \mathbf{a}$ is described by:

$$\mathbb{E}[\mathbf{B}|\mathbf{A}=\mathbf{a}] = \boldsymbol{\mu}_{\mathrm{B}} + \Sigma_{\mathrm{BA}}\Sigma_{\mathrm{A}}^{-1}(\mathbf{a}-\boldsymbol{\mu}_{\mathrm{A}})$$
$$\mathrm{var}(\mathbf{B}|\mathbf{A}=\mathbf{a}) = \Sigma_{\mathrm{B}} - \Sigma_{\mathrm{BA}}\Sigma_{\mathrm{A}}^{-1}\Sigma_{\mathrm{AB}} \quad \text{(S15)}$$

These expressions allow us to compute the conditional expectation and variance-covariance matrix of the π_1-fraction estimates given the full-sample estimates. Explicit forms for the four required quantities: $\boldsymbol{\mu}_{\mathrm{B}}$, Σ_{B}, Σ_{AB}, Σ_{BA} are derived in what follows.

We first derive the variance and covariance terms relevant to regression estimates, $\hat{\beta}_X^1$ and $\hat{\beta}_Y^1$, derived from the π_1-fraction of the full dataset, i.e. obtain Σ_{B}. In this π_1-fraction sub-sample, there are approximately $\pi_1 n_X$ individuals with exposure data, $\pi_1 n_Y$ with outcome data and $\pi_1 n_{\mathrm{overlap}}$ with both. Let the genotype matrix for the exposure sub-sample for a given genetic variant be denoted:

$$\boldsymbol{G}_X^1 = \begin{bmatrix} \boldsymbol{G}_{\mathrm{overlap}}^1 \\ \boldsymbol{G}_X^{1*} \end{bmatrix} \in \mathbb{R}^{\pi_1 n_X \times 2} \quad \text{(S16)}$$

With $\boldsymbol{X}^1 \in \mathbb{R}^{\pi_1 n_X}$ representing the exposure values in this sub-sample, the least squares estimator of the variant-exposure association is given by:

$$\hat{\boldsymbol{\beta}}_X^1 = (\boldsymbol{G}_X^{1\,\mathrm{T}}\boldsymbol{G}_X^1)^{-1}\boldsymbol{G}_X^{1\,\mathrm{T}}\boldsymbol{X}^1 \approx \frac{\boldsymbol{C}}{\pi_1 n_X}\boldsymbol{G}_X^{1\,\mathrm{T}}\boldsymbol{X}^1 \quad \text{(S17)}$$

where $\boldsymbol{C}$ is defined above. From prior derivations, we note that $\mathrm{var}(\hat{\beta}_X) \approx \frac{\mathrm{var}(X)}{n_X 2(\mathrm{maf})(1-\mathrm{maf})}$. Analogously, under random sampling,

$$\mathrm{var}(\hat{\beta}_X^1) \approx \frac{\mathrm{var}(X)}{\pi_1 n_X 2(\mathrm{maf})(1-\mathrm{maf})} \quad \text{(S18)}$$

and $\mathbb{E}[\hat{\beta}_X^1] = \beta_X$. Again, a symmetric derivation provides $\mathbb{E}[\hat{\beta}_Y^1] = \beta_Y$ and $\mathrm{var}(\hat{\beta}_Y^1) \approx \frac{\mathrm{var}(Y)}{\pi_1 n_Y 2(\mathrm{maf})(1-\mathrm{maf})}$. To derive $\mathrm{cov}(\hat{\beta}_X^1, \hat{\beta}_Y^1)$, we note that it follows from Equations (S10) and (S15) and the fact that $\mathbf{cov}(\boldsymbol{X}_{\mathrm{overlap}}^1, \boldsymbol{Y}_{\mathrm{overlap}}^1) = \mathrm{cov}(X,Y)\cdot\mathbb{I}_{\pi_1 n_{\mathrm{overlap}}}$, that:

$$\mathbf{cov}(\hat{\boldsymbol{\beta}}_X^1, \hat{\boldsymbol{\beta}}_Y^1) \approx \frac{1}{\pi_1 n_X \cdot \pi_1 n_Y}\mathbf{cov}(\boldsymbol{C}\boldsymbol{G}_{\mathrm{overlap}}^{1\;\mathrm{T}}\boldsymbol{X}_{\mathrm{overlap}}^1, \boldsymbol{C}\boldsymbol{G}_{\mathrm{overlap}}^{1\;\mathrm{T}}\boldsymbol{Y}_{\mathrm{overlap}}^1)$$
$$\approx \frac{\pi_1 n_{\mathrm{overlap}}\mathrm{cov}(X,Y)}{\pi_1 n_X \cdot \pi_1 n_Y}\boldsymbol{C} \quad \text{(S19)}$$

Again letting $\lambda = \frac{n_{\mathrm{overlap}}\rho}{\sqrt{n_X n_Y}}$, this gives:

$$\mathrm{cov}(\hat{\beta}_X^1, \hat{\beta}_Y^1) \approx \frac{1}{\pi_1}\cdot\frac{1}{2(\mathrm{maf})(1-\mathrm{maf})}\cdot\lambda\sqrt{\frac{\mathrm{var}(X)\mathrm{var}(Y)}{n_X n_Y}} \approx \frac{1}{\pi_1}\mathrm{cov}(\hat{\beta}_X, \hat{\beta}_Y) \quad \text{(S20)}$$

and therefore, $\Sigma_{\mathrm{B}} = \frac{1}{\pi_1}\Sigma_{\mathrm{A}}$, while $\boldsymbol{\mu}_{\mathrm{B}} = \begin{bmatrix}\beta_X \\ \beta_Y\end{bmatrix} = \boldsymbol{\mu}_{\mathrm{A}}$.

Lastly, $\sum_{\mathbf{AB}}$ = $\mathbf{cov}\left(\begin{bmatrix}\hat{\beta}_X\\\hat{\beta}_Y\end{bmatrix},\begin{bmatrix}\hat{\beta}_X^1\\\hat{\beta}_Y^1\end{bmatrix}\right)$. As $\begin{bmatrix}\hat{\beta}_X\\\hat{\beta}_Y\end{bmatrix} \approx \pi_1\begin{bmatrix}\hat{\beta}_X^1\\\hat{\beta}_Y^1\end{bmatrix} + (1-\pi_1)\begin{bmatrix}\hat{\beta}_X^2\\\hat{\beta}_Y^2\end{bmatrix}$ in which $\begin{bmatrix}\hat{\beta}_X^1\\\hat{\beta}_Y^1\end{bmatrix}$ and $\begin{bmatrix}\hat{\beta}_X^2\\\hat{\beta}_Y^2\end{bmatrix}$ are independent ($\hat{\beta}_X^2$ and $\hat{\beta}_Y^2$ denote the variant-exposure and variant-outcome association estimates in the second ($1-\pi_1$) fraction), we can obtain the following:

$$\textstyle\sum_{\mathbf{AB}} = \mathbf{cov}\left(\begin{bmatrix}\hat{\beta}_X\\\hat{\beta}_Y\end{bmatrix},\begin{bmatrix}\hat{\beta}_X^1\\\hat{\beta}_Y^1\end{bmatrix}\right) = \mathbf{cov}\left(\pi_1\begin{bmatrix}\hat{\beta}_X^1\\\hat{\beta}_Y^1\end{bmatrix}+(1-\pi_1)\begin{bmatrix}\hat{\beta}_X^2\\\hat{\beta}_Y^2\end{bmatrix},\begin{bmatrix}\hat{\beta}_X^1\\\hat{\beta}_Y^1\end{bmatrix}\right) = \pi\mathbf{var}\left(\begin{bmatrix}\hat{\beta}_X^1\\\hat{\beta}_Y^1\end{bmatrix}\right) = \pi\sum_{\mathbf{B}} = \sum_{\mathbf{A}} \tag{S21}$$

Similarly, $\sum_{\mathbf{BA}}$ = $\mathbf{cov}\left(\begin{bmatrix}\hat{\beta}_X^1\\\hat{\beta}_Y^1\end{bmatrix},\begin{bmatrix}\hat{\beta}_X\\\hat{\beta}_Y\end{bmatrix}\right) = \sum_{\mathbf{A}}$.

Thus, the conditional distribution is:

$$\begin{bmatrix}\hat{\beta}_X^1\\\hat{\beta}_Y^1\end{bmatrix} \mid \begin{bmatrix}\hat{\beta}_X\\\hat{\beta}_Y\end{bmatrix} \sim N\left(\begin{bmatrix}\hat{\beta}_X\\\hat{\beta}_Y\end{bmatrix},\left(\frac{1-\pi_1}{\pi_1}\right)\textstyle\sum_{\mathbf{A}}\right) \tag{S22}$$

$$\hat{\sigma}_X^2 \approx \mathrm{var}(\hat{\beta}_X) \approx \frac{\mathrm{var}(X)}{n_X 2(\mathrm{maf})(1-\mathrm{maf})},\quad \hat{\sigma}_Y^2 \approx \mathrm{var}(\hat{\beta}_Y) \approx \frac{\mathrm{var}(Y)}{n_Y 2(\mathrm{maf})(1-\mathrm{maf})}$$

Using the definition of $\sum_{\mathbf{A}}$ given in Equation (S13), this conditional distribution can be written as follows:

$$\begin{bmatrix}\hat{\beta}_X^1\\\hat{\beta}_Y^1\end{bmatrix} \mid \begin{bmatrix}\hat{\beta}_X\\\hat{\beta}_Y\end{bmatrix} \sim N\left(\begin{bmatrix}\hat{\beta}_X\\\hat{\beta}_Y\end{bmatrix},\left(\frac{1-\pi_1}{\pi_1}\right)\begin{bmatrix}\sigma_X^2 & \lambda\sigma_X\sigma_Y\\\lambda\sigma_X\sigma_Y & \sigma_X^2\end{bmatrix}\right) \tag{S23}$$

This final expression enables efficient simulation of variant-trait association estimates in split samples based on GWAS summary statistics alone.

Association estimates in the $(1-\pi_1)$-subset, i.e. $\hat{\beta}_X^2$ and $\hat{\beta}_Y^2$, are obtained using asymptotic linearity of maximum likelihood estimates, as shown in Equation (2) in the main manuscript. To avoid biases due to sample overlap, this sample splitting approach can be extended to a 3-split framework, in which the $(1-\pi_1)$-fraction is further randomly split into two independent sub-fractions of relative sizes π_2 and $1-\pi_2$. For a given variant, let $\hat{\beta}_X^{2\mathrm{a}}$ and $\hat{\beta}_Y^{2\mathrm{a}}$ denote the variant-exposure and variant-outcome association estimates in the π_2-subset. In a similar process to that of the first split, $\hat{\beta}_X^{2\mathrm{a}}$ and $\hat{\beta}_Y^{2\mathrm{a}}$ can be simulated conditional on the association estimates in the full $(1-\pi_1)$-fraction, $\hat{\beta}_X^2$ and $\hat{\beta}_Y^2$. The conditional distribution of the vector $\begin{bmatrix}\hat{\beta}_X^{2\mathrm{a}}\\\hat{\beta}_Y^{2\mathrm{a}}\end{bmatrix}$ given the full $(1-\pi_1)$-fraction estimates $\begin{bmatrix}\hat{\beta}_X^2\\\hat{\beta}_Y^2\end{bmatrix}$ is derived in the same manner as Equation (S22). It can be easily shown that this conditional distribution takes the form:

$$\begin{bmatrix}\hat{\beta}_X^{2\mathrm{a}}\\\hat{\beta}_Y^{2\mathrm{a}}\end{bmatrix} \mid \begin{bmatrix}\hat{\beta}_X^2\\\hat{\beta}_Y^2\end{bmatrix} \sim N\left(\begin{bmatrix}\hat{\beta}_X^2\\\hat{\beta}_Y^2\end{bmatrix},\left(\frac{1-\pi_2}{\pi_2}\right)\left(\frac{1}{1-\pi_1}\right)\begin{bmatrix}\sigma_X^2 & \lambda\sigma_X\sigma_Y\\\lambda\sigma_X\sigma_Y & \sigma_X^2\end{bmatrix}\right) \tag{S24}$$

$\hat{\beta}_Y^{2\mathrm{b}}$, variant-outcome association estimates in the $(1-\pi_2)$-subsample, are generated according to the asymptotic linearity of maximum likelihood estimates, i.e. $\hat{\beta}_{Y_j}^{2\mathrm{b}} \approx \frac{\hat{\beta}_{Y_j}^2-\pi_2\hat{\beta}_{Y_j}^{2\mathrm{a}}}{1-\pi_2}$. For each variant, this 3-split framework yields independent association estimates and corresponding standard errors, $\{\hat{\beta}_X^{2\mathrm{a}}, \sigma_X^{2\mathrm{a}}, \hat{\beta}_Y^{2\mathrm{b}}, \sigma_X^{2\mathrm{b}}\}$, which are subsequently inputted into the 2-sample MR method of choice.

Inserting randomness

The main concept of MR-SimSS, as described above, can also be understood in the form of "inserting randomness". For a given genetic variant, we assume the marginal distribution of $\hat{\beta}_X$ and $\hat{\beta}_Y$, the variant-exposure and variant-outcome association estimates, takes the form derived in the first section above:

$$\begin{bmatrix} \hat{\beta}_X \\ \hat{\beta}_Y \end{bmatrix} \sim N\left(\begin{bmatrix} \beta_X \\ \beta_Y \end{bmatrix}, \begin{bmatrix} \sigma_X^2 & \lambda\sigma_X\sigma_Y \\ \lambda\sigma_X\sigma_Y & \sigma_X^2 \end{bmatrix} \right) \tag{S25}$$

The idea of splitting the full dataset into two disjoint fractions, π_1 and $1-\pi_1$, can be thought of as equivalent to introducing an additional bivariate random vector with correlated components:

$$\mathbf{U} = \begin{bmatrix} U_X \\ U_Y \end{bmatrix} \sim N\left(\begin{bmatrix} 0 \\ 0 \end{bmatrix}, \left(\frac{1-\pi_1}{\pi_1}\right) \begin{bmatrix} \sigma_X^2 & \lambda\sigma_X\sigma_Y \\ \lambda\sigma_X\sigma_Y & \sigma_X^2 \end{bmatrix} \right) \tag{S26}$$

and considering:

$$\begin{bmatrix} \hat{\beta}_X^1 \\ \hat{\beta}_Y^1 \end{bmatrix} = \begin{bmatrix} \hat{\beta}_X \\ \hat{\beta}_Y \end{bmatrix} + \begin{bmatrix} U_X \\ U_Y \end{bmatrix}, \quad \begin{bmatrix} \hat{\beta}_X^2 \\ \hat{\beta}_Y^2 \end{bmatrix} = \begin{bmatrix} \hat{\beta}_X \\ \hat{\beta}_Y \end{bmatrix} - \frac{\pi_1}{1-\pi_1} \begin{bmatrix} U_X \\ U_Y \end{bmatrix} = \frac{1}{1-\pi_1} \begin{bmatrix} \hat{\beta}_X - \pi_1 \cdot \hat{\beta}_X^1 \\ \hat{\beta}_Y - \pi_1 \cdot \hat{\beta}_X^1 \end{bmatrix} \tag{S27}$$

Using the definitions given in Equations (S26) and (S27), it can be easily shown that $\left[\hat{\beta}_X^1, \hat{\beta}_Y^1, \hat{\beta}_X^2, \hat{\beta}_Y^2\right]^T$ follows the following joint distribution:

$$\begin{bmatrix} \hat{\beta}_X^1 \\ \hat{\beta}_Y^1 \\ \hat{\beta}_X^2 \\ \hat{\beta}_Y^2 \end{bmatrix} \sim N\left(\begin{bmatrix} \beta_X \\ \beta_Y \\ \beta_X \\ \beta_Y \end{bmatrix}, \begin{bmatrix} \frac{1}{\pi_1}\sigma_X^2 & \frac{\lambda}{\pi_1}\sigma_X\sigma_Y & 0 & 0 \\ \frac{\lambda}{\pi_1}\sigma_X\sigma_Y & \frac{1}{\pi_1}\sigma_Y^2 & 0 & 0 \\ 0 & 0 & \frac{1}{1-\pi_1}\sigma_X^2 & \frac{\lambda}{1-\pi_1}\sigma_X\sigma_Y \\ 0 & 0 & \frac{\lambda}{1-\pi_1}\sigma_X\sigma_Y & \frac{1}{1-\pi_1}\sigma_Y^2 \end{bmatrix} \right) \tag{S28}$$

If $\lambda = \frac{n_{\text{overlap}}\rho}{\sqrt{n_X n_Y}} = 0$, i.e. there is no sample overlap between exposure and outcome GWASs, Equation (S28) is an equivalent definition of the two-split variant of MR-SimSS and a standard three-sample MR study is essentially created in which $\hat{\beta}_X^1$ can be used for instrument selection and $\left(\hat{\beta}_X^2, \hat{\beta}_Y^2\right)$ can be used for statistical inference using standard two-sample MR methods.

However, if sample overlap exists ($\lambda \neq 0$), this two-split approach may still incur weak instrument bias in the direction of confounding. To address this, we extend MR-SimSS to a three-split framework, as described in the previous section. This additional split can be thought of as introducing another bivariate random vector, in addition to $\mathbf{U}$, defined as:

$$\mathbf{V} = \begin{bmatrix} V_X \\ V_Y \end{bmatrix} \sim N\left(\begin{bmatrix} 0 \\ 0 \end{bmatrix}, \left(\frac{1-\pi_2}{\pi_2}\right) \begin{bmatrix} \sigma_X^2 & \lambda\sigma_X\sigma_Y \\ \lambda\sigma_X\sigma_Y & \sigma_X^2 \end{bmatrix} \right) \tag{S29}$$

In a similar manner, we then consider:

$$\begin{bmatrix}\hat{\beta}_X^{2a}\\ \hat{\beta}_Y^{2a}\end{bmatrix}=\begin{bmatrix}\hat{\beta}_X^{2}\\ \hat{\beta}_Y^{2}\end{bmatrix}+\begin{bmatrix}V_X\\ V_Y\end{bmatrix},\qquad \begin{bmatrix}\hat{\beta}_X^{2b}\\ \hat{\beta}_Y^{2b}\end{bmatrix}=\begin{bmatrix}\hat{\beta}_X^{2}\\ \hat{\beta}_Y^{2}\end{bmatrix}-\frac{\pi_2}{1-\pi_2}\begin{bmatrix}V_X\\ V_Y\end{bmatrix}=\frac{1}{1-\pi_2}\begin{bmatrix}\hat{\beta}_X^{2}-\pi_2\cdot\hat{\beta}_X^{2a}\\ \hat{\beta}_Y^{2}-\pi_2\cdot\hat{\beta}_Y^{2a}\end{bmatrix}\tag{S30}$$

Putting all of the above together, the joint distribution of $\left[\hat{\beta}_X^1, \hat{\beta}_Y^1, \hat{\beta}_X^{2a}, \hat{\beta}_Y^{2a}, \hat{\beta}_X^{2b}, \hat{\beta}_Y^{2b}\right]^T$ can be written as:

$$\begin{bmatrix}\hat{\beta}_X^1\\ \hat{\beta}_Y^1\\ \hat{\beta}_X^{2a}\\ \hat{\beta}_Y^{2a}\\ \hat{\beta}_X^{2b}\\ \hat{\beta}_Y^{2b}\end{bmatrix}\sim N\left(\begin{bmatrix}\beta_X\\ \beta_Y\\ \beta_X\\ \beta_Y\\ \beta_X\\ \beta_Y\end{bmatrix},\begin{bmatrix}\frac{\sigma_X^2}{\pi_1} & \frac{\lambda\sigma_X\sigma_Y}{\pi_1} & 0 & 0 & 0 & 0\\ \frac{\lambda\sigma_X\sigma_Y}{\pi_1} & \frac{\sigma_Y^2}{\pi_1} & 0 & 0 & 0 & 0\\ 0 & 0 & \frac{\sigma_X^2}{\pi_2(1-\pi_1)} & \frac{\lambda\sigma_X\sigma_Y}{\pi_2(1-\pi_1)} & 0 & 0\\ 0 & 0 & \frac{\lambda\sigma_X\sigma_Y}{\pi_2(1-\pi_1)} & \frac{\sigma_Y^2}{\pi_2(1-\pi_1)} & 0 & 0\\ 0 & 0 & 0 & 0 & \frac{\sigma_X^2}{(1-\pi_2)(1-\pi_1)} & \frac{\lambda\sigma_X\sigma_Y}{(1-\pi_2)(1-\pi_1)}\\ 0 & 0 & 0 & 0 & \frac{\lambda\sigma_X\sigma_Y}{(1-\pi_2)(1-\pi_1)} & \frac{\sigma_Y^2}{(1-\pi_2)(1-\pi_1)}\end{bmatrix}\right)\tag{S31}$$

Equation (S31) is therefore an equivalent description of our three-split framework. This is again analogous to a standard three-sample MR study in which $\hat{\beta}_X^1$ is used for instrument selection and $\left(\hat{\beta}_X^{2a}, \hat{\beta}_Y^{2b}\right)$ can be used for exposure-outcome effect estimation using a two-sample MR method of choice.

Binary outcome

Our derivation of the conditional distribution above is provided in the context of a continuous exposure, X and a continuous outcome, Y. We will now show that the same final expression for the conditional distribution holds even if the outcome trait is binary. Similarly, the conditional distribution takes the form:

$$\begin{bmatrix}\hat{\beta}_X^1\\ \hat{\beta}_Y^1\end{bmatrix}\Big|\begin{bmatrix}\hat{\beta}_X\\ \hat{\beta}_Y\end{bmatrix}\sim N\left(\begin{bmatrix}\hat{\beta}_X\\ \hat{\beta}_Y\end{bmatrix},\left(\frac{1-\pi_1}{\pi_1}\right)\Sigma_{\mathrm{A}}\right)\tag{S32}$$

in which $\Sigma_{\mathrm{A}}=\begin{bmatrix}\mathrm{var}(\hat{\beta}_X) & \mathrm{cov}(\hat{\beta}_X,\hat{\beta}_Y)\\ \mathrm{cov}(\hat{\beta}_X,\hat{\beta}_Y) & \mathrm{var}(\hat{\beta}_Y)\end{bmatrix}$ and so, we need to obtain expressions for $\mathrm{cov}(\hat{\beta}_X,\hat{\beta}_Y)$ and $\mathrm{var}(\hat{\beta}_Y)$ with respect to the binary outcome, Y.

For binary $\boldsymbol{Y}$, we assume the logistic model: $\mathrm{logit}(\mathbb{P}(\boldsymbol{Y}=1))=\boldsymbol{G_Y}\boldsymbol{\beta_Y}$, where $\boldsymbol{\beta_Y}=\begin{bmatrix}\beta_{Y_0}\\ \beta_Y\end{bmatrix}$ and $\boldsymbol{G_Y}\in\mathbb{R}^{n_Y\times 2}$ is the design matrix containing the genotypes of individuals with outcome information:

$$\boldsymbol{G_Y}=\begin{bmatrix}1 & G_Y\\ \vdots & \vdots\\ 1 & G_{n_Y}\end{bmatrix}=\begin{bmatrix}\boldsymbol{G}_{\text{overlap}}\\ \boldsymbol{G}_{\boldsymbol{Y}}^{*}\end{bmatrix}\tag{S33}$$

The ML-estimated logistic regression coefficient vector, obtained from regressing $\boldsymbol{Y}$ on $\boldsymbol{G_Y}$, is denoted as $\hat{\boldsymbol{\beta}}_{\boldsymbol{Y}}$ and is represented by:

$$\hat{\boldsymbol{\beta}}_{\boldsymbol{Y}} = \left(\boldsymbol{G}_{\boldsymbol{Y}}^{\mathrm{T}}\widehat{\boldsymbol{W}}\boldsymbol{G}_{\boldsymbol{Y}}\right)^{-\mathbf{1}}\boldsymbol{G}_{\boldsymbol{Y}}^{\mathrm{T}}(\boldsymbol{Y} - \hat{\mathbf{p}}) \tag{S34}$$

where:

$$\widehat{\boldsymbol{W}} = \begin{bmatrix} \hat{p}_1(1-\hat{p}_1) & \cdots & 0 \\ \vdots & \ddots & \vdots \\ 0 & \cdots & \hat{p}_{n_Y}(1-\hat{p}_{n_Y}) \end{bmatrix} \tag{S35}$$

$$\hat{p}_i = \frac{\exp(\hat{\beta}_{Y_0} + \hat{\beta}_Y G_i)}{1 + \exp(\hat{\beta}_{Y_0} + \hat{\beta}_Y G_i)} \tag{S36}$$

and:

$$\hat{\mathbf{p}} = (\hat{p}_1, \dots, \hat{p}_{n_Y})^{\mathrm{T}} \tag{S37}$$

It is well known that $\mathrm{var}(\hat{\boldsymbol{\beta}}_{\boldsymbol{Y}}) \approx \left(\boldsymbol{G}_{\boldsymbol{Y}}^{\mathrm{T}}\widehat{\boldsymbol{W}}\boldsymbol{G}_{\boldsymbol{Y}}\right)^{-\mathbf{1}}$, which can be re-expressed as:

$$\boldsymbol{G}_{\boldsymbol{Y}}^{\mathrm{T}}\widehat{\boldsymbol{W}}\boldsymbol{G}_{\boldsymbol{Y}} = \begin{bmatrix} \sum_{i=1}^{n_Y} \hat{p}_i(1-\hat{p}_i) & \sum_{i=1}^{n_Y} G_i\hat{p}_i(1-\hat{p}_i) \\ \sum_{i=1}^{n_Y} G_i\hat{p}_i(1-\hat{p}_i) & \sum_{i=1}^{n_Y} G_i^2\,\hat{p}_i(1-\hat{p}_i) \end{bmatrix} \approx n_Y \begin{bmatrix} \mathbb{E}[P(1-P)] & \mathbb{E}[GP(1-P)] \\ \mathbb{E}[GP(1-P)] & \mathbb{E}[G^2P(1-P)] \end{bmatrix} \tag{S38}$$

where P is the random variable that takes value $\mathbb{P}(Y=1|\mathrm{G}) = \frac{\exp(\hat{\beta}_{Y_0} + \hat{\beta}_Y \mathrm{G})}{1+\exp(\hat{\beta}_{Y_0} + \hat{\beta}_Y \mathrm{G})}$ for genotype G, and G is the binomial distribution for genotype assuming Hardy Weinberg Equilibrium. Denoting

$$\mathsf{C}_{\boldsymbol{Y}} \approx \begin{bmatrix} \mathbb{E}[P(1-P)] & \mathbb{E}[GP(1-P)] \\ \mathbb{E}[GP(1-P)] & \mathbb{E}[G^2P(1-P)] \end{bmatrix}^{-\mathbf{1}} \tag{S39}$$

it follows that $\left(\boldsymbol{G}_{\boldsymbol{Y}}^{\mathrm{T}}\widehat{\boldsymbol{W}}\boldsymbol{G}_{\boldsymbol{Y}}\right)^{-\mathbf{1}} \approx \frac{\mathsf{C}_Y}{n_Y}$.

Recall from above that for the continuous exposure, we had $\left(\boldsymbol{G}_{\boldsymbol{X}}^{\mathrm{T}}\boldsymbol{G}_{\boldsymbol{X}}\right)^{-\mathbf{1}} \approx \frac{\mathsf{C}_{\boldsymbol{X}}}{n_X}$ in which

$$\mathsf{C}_{\boldsymbol{X}} \approx \begin{bmatrix} 1 & 2(\mathrm{maf}) \\ 2(\mathrm{maf}) & [4(\mathrm{maf})^2 + 2(\mathrm{maf})(1-\mathrm{maf})] \end{bmatrix}^{-1} \tag{S40}$$

Then,

$$\begin{aligned} \mathbf{cov}(\hat{\boldsymbol{\beta}}_{\boldsymbol{X}}, \hat{\boldsymbol{\beta}}_{\boldsymbol{Y}}) &\approx \mathbf{cov}\Big(\frac{\mathsf{C}_{\boldsymbol{X}}}{n_X}\boldsymbol{G}_{\boldsymbol{X}}^{\mathrm{T}}\boldsymbol{X}, \frac{\mathsf{C}_{\boldsymbol{Y}}}{n_X}\boldsymbol{G}_{\boldsymbol{Y}}^{\mathrm{T}}\boldsymbol{Y}\Big) \\ &= \frac{1}{n_X n_Y}\mathbf{cov}(\mathsf{C}_{\boldsymbol{X}}\boldsymbol{G}_{\mathbf{overlap}}^{\mathrm{T}}\boldsymbol{X}_{\mathbf{overlap}}, \mathsf{C}_{\boldsymbol{Y}}\boldsymbol{G}_{\mathbf{overlap}}^{\mathrm{T}}\boldsymbol{Y}_{\mathbf{overlap}}) = \frac{\mathrm{cov}(X,Y)}{n_X n_Y}\,\mathsf{C}_{\boldsymbol{X}}\boldsymbol{G}_{\mathbf{overlap}}\boldsymbol{G}_{\mathbf{overlap}}^{\mathrm{T}}\boldsymbol{C}_{\mathrm{Y}}^{\mathrm{T}} \end{aligned} \tag{S42}$$

As $\boldsymbol{G}_{\mathbf{overlap}}^{\mathrm{T}}\boldsymbol{G}_{\mathbf{overlap}} = n_{\mathrm{overlap}}\boldsymbol{C}_{\boldsymbol{X}}^{-1}$ and $\mathsf{C}_{\boldsymbol{Y}} = \boldsymbol{C}_{\mathrm{Y}}^{\mathrm{T}}$, we have $\mathbf{cov}(\hat{\boldsymbol{\beta}}_{\boldsymbol{X}}, \hat{\boldsymbol{\beta}}_{\boldsymbol{Y}}) \approx \frac{n_{\mathrm{overlap}}\mathrm{cov}(X,Y)\mathsf{C}_{\boldsymbol{Y}}}{n_X n_Y}$.

We want to find an expression for $\text{cov}(\hat{\beta}_X, \hat{\beta}_Y)$, the lower right element of the matrix $\mathbf{cov(\hat{\beta}_X, \hat{\beta}_Y)}$. As the marginal SNP-outcome effects are likely very small, the random variable P can be treated as effectively equal to $\mathbb{P}(Y=1)$. This allows us to simplify the various elements of the matrix $\mathbf{C_Y}$ as follows:

- $\mathbb{E}[P(1-P)] \approx \mathbb{P}(Y=1)\big(1-\mathbb{P}(Y=1)\big) \approx \text{var}(Y)$
- $\mathbb{E}[G^2P(1-P)] \approx \mathbb{P}(Y=1)\big(1-\mathbb{P}(Y=1)\big)\mathbb{E}[G^2] \approx \text{var}(Y)\mathbb{E}[G^2]$
- $\mathbb{E}[GP(1-P)] \approx \mathbb{P}(Y=1)\big(1-\mathbb{P}(Y=1)\big)\mathbb{E}[G] \approx \text{var}(Y)\mathbb{E}[G]$

Thus, the lower right element of $\mathbf{C_Y}$ is $\frac{\mathbb{E}[P(1-P)]}{\mathbb{E}[P(1-P)]\mathbb{E}[G^2P(1-P)]-(\mathbb{E}[GP(1-P)])^2} \approx \frac{\text{var}(Y)}{(\text{var}(Y))^2(\mathbb{E}[G^2]-(\mathbb{E}[G])^2)} = \frac{1}{\text{var}(Y)\text{var}(G)}$. As $\text{var}(G) \approx 2(\text{maf})(1-\text{maf})$, assuming Hardy-Weinberg equilibrium, and $\text{cov}(X,Y) = \rho\sqrt{\text{var}(X)\text{var}(Y)}$, where ρ is the exposure-outcome correlation, we get:

$$\text{cov}(\hat{\beta}_X, \hat{\beta}_Y) \approx \frac{n_{\text{overlap}}\rho\sqrt{\text{var}(X)}}{n_X n_Y \cdot \sqrt{\text{var}(Y)} \cdot 2(\text{maf})(1-\text{maf})} \tag{S43}$$

From above, $\text{var}(\hat{\boldsymbol{\beta}}_Y) \approx \frac{\mathbf{C_Y}}{n_Y}$, with $\text{var}(\hat{\beta}_Y)$ being equal to the lower right element of $\mathbf{C_Y}$ divided by n_Y, i.e. $\text{var}(\hat{\beta}_Y) \approx \frac{1}{\text{var}(Y)\cdot n_Y 2(\text{maf})(1-\text{maf})}$.

An expression for the conditional distribution is therefore given by:

$$\begin{bmatrix}\hat{\beta}_X^1 \\ \hat{\beta}_Y^1\end{bmatrix} \mid \begin{bmatrix}\hat{\beta}_X \\ \hat{\beta}_Y\end{bmatrix} \sim N\left(\begin{bmatrix}\hat{\beta}_X \\ \hat{\beta}_Y\end{bmatrix}, \left(\frac{1-\pi_1}{\pi_1}\right)\left(\frac{1}{2(\text{maf})(1-\text{maf})}\right)\begin{bmatrix}\frac{\text{var}(X)}{n_X} & \frac{n_{\text{overlap}}\rho\sqrt{\text{var}(X)}}{n_X n_Y \cdot \sqrt{\text{var}(Y)}} \\ \frac{n_{\text{overlap}}\rho\sqrt{\text{var}(X)}}{n_X n_Y \cdot \sqrt{\text{var}(Y)}} & \frac{1}{n_Y \cdot \text{var}(Y)}\end{bmatrix}\right) \tag{S44}$$

In this case, we make use of standard error estimates

$$\hat{\sigma}_X^2 \approx \text{var}(\hat{\beta}_X) \approx \frac{\text{var}(X)}{n_X 2(\text{maf})(1-\text{maf})}, \quad \hat{\sigma}_Y^2 \approx \text{var}(\hat{\beta}_Y) \approx \frac{1}{\text{var}(Y)\cdot n_Y 2(\text{maf})(1-\text{maf})} \tag{S45}$$

and re-express the conditional distribution as follows, with $\lambda = \frac{n_{\text{overlap}}\rho}{\sqrt{n_X n_Y}}$:

$$\begin{bmatrix}\hat{\beta}_X^1 \\ \hat{\beta}_Y^1\end{bmatrix} \mid \begin{bmatrix}\hat{\beta}_X \\ \hat{\beta}_Y\end{bmatrix} \sim N\left(\begin{bmatrix}\hat{\beta}_X \\ \hat{\beta}_Y\end{bmatrix}, \left(\frac{1-\pi_1}{\pi_1}\right)\begin{bmatrix}\hat{\sigma}_X^2 & \lambda\sigma_X\sigma_Y \\ \lambda\sigma_X\sigma_Y & \hat{\sigma}_Y^2\end{bmatrix}\right) \tag{S46}$$

Derivation of standard error for MR-SimSS estimator

As demonstrated in Equation (3) in the main manuscript, the final MR-SimSS causal effect estimate, $\hat{\beta}$, is defined as the average across N_{iter} iterations, i.e. $\hat{\beta} = \frac{1}{N_{\text{iter}}}\sum_{k=1}^{N_{\text{iter}}}\hat{\beta}^{(k)}$, in which $\hat{\beta}^{(k)}$ is the causal

effect estimate from iteration k of the simulated sample splitting process, computed using a summary-level MR method such as IVW or MR-RAPS.

We derive an estimator of the standard error of the limiting MR-SimSS estimate $\hat{\beta} = \lim_{N_{\text{iter}}\to\infty} \frac{1}{N_{\text{iter}}}\sum_{k=1}^{N_{\text{iter}}} \hat{\beta}^{(k)}$, which we use to approximate the standard error of $\frac{1}{N_{\text{iter}}}\sum_{k=1}^{N_{\text{iter}}} \hat{\beta}^{(k)}$. First, by the law of large numbers and noticing $\mathbb{E}\left[\hat{\beta}^{(*)} \mid \hat{\beta}\right] = \hat{\beta}$ where $\hat{\beta}^{(*)}$ is the causal effect estimate obtained from an iteration of MR-SimSS, we get:

$$\frac{1}{N_{\text{iter}}}\sum_{k=1}^{N_{\text{iter}}}\left(\hat{\beta}^{(k)} - \hat{\beta}\right)^2 \rightarrow \mathbb{E}\left[\left(\hat{\beta}^{(*)} - \hat{\beta}\right)^2 \mid \hat{\beta}\right] = \mathbb{E}\left[\left(\hat{\beta}^{(*)}\right)^2 \mid \hat{\beta}\right] - \hat{\beta}^2 \quad \text{(S47)}$$

Note that in the above expressions, the conditional distribution of the MR-SimSS iterates $\hat{\beta}^{(*)}$ around their long-run average $\hat{\beta}$ depends on the vectors of observed variant-exposure and variant-outcome associations, which determine both $\hat{\beta}^{(k)}$ and their long-run Monte Carlo average $\hat{\beta}$. In a slight abuse of notation, we denote this conditional distribution simply as $\left(\hat{\beta}^{(*)} - \hat{\beta}\right)^2 \mid \hat{\beta}$. The above implies the following, with the approximation being arbitrarily close for large N_{iter}:

$$\frac{1}{N_{\text{iter}}}\sum_{k=1}^{N_{\text{iter}}}\left(\hat{\beta}^{(*)} - \hat{\beta}\right)^2 \approx \mathbb{E}\left[\left(\hat{\beta}^{(*)}\right)^2 \mid \hat{\beta}\right] - \hat{\beta}^2 - \beta^2 + \beta^2 \quad \text{(S48)}$$

Taking expectations again, over the distribution of $\hat{\beta}$:

$$\begin{aligned}\frac{1}{N_{\text{iter}}}\mathbb{E}\left[\sum_{k=1}^{N_{\text{iter}}}\left(\hat{\beta}^{(*)} - \hat{\beta}\right)^2\right] &\approx \mathbb{E}\left[\left(\hat{\beta}^{(*)}\right)^2\right] - \beta^2 - \left(\mathbb{E}\left[\left(\hat{\beta}\right)^2\right] - \beta^2\right) \\ &= \operatorname{var}\left[\hat{\beta}^{(*)}\right] - \operatorname{var}\left[\hat{\beta}\right]\end{aligned} \quad \text{(S49)}$$

using the well-known identity $\mathbb{E}[X^2] - (\mathbb{E}[X])^2 = \operatorname{var}(X)$ and $\mathbb{E}\left[\hat{\beta}^{(*)}\right] = \mathbb{E}\left[\mathbb{E}\left[\hat{\beta}^{(*)}\middle|\hat{\beta}\right]\right] = \mathbb{E}\left[\hat{\beta}\right] = \beta$. Therefore, an unbiased expression for $\operatorname{var}\left[\hat{\beta}\right]$ is:

$$\begin{aligned}\operatorname{var}\left[\hat{\beta}\right] &= \operatorname{var}\left[\hat{\beta}^{(k)}\right] - \frac{1}{N_{\text{iter}}}\mathbb{E}\left[\sum_{k=1}^{N_{\text{iter}}}\left(\hat{\beta}^{(k)} - \hat{\beta}\right)^2\right] \\ &= \frac{1}{N_{\text{iter}}}\sum_{k=1}^{N_{\text{iter}}}[\operatorname{se}(\hat{\beta}^{(k)})]^2 - \frac{1}{N_{\text{iter}}}\sum_{k=1}^{N_{\text{iter}}}[\hat{\beta}^{(k)} - \hat{\beta}]^2\end{aligned} \quad \text{(S50)}$$

Rearranging this expression gives Equation (4) in the main manuscript:

$$\operatorname{se}(\hat{\beta}) = \sqrt{\frac{1}{N_{\text{iter}}}\sum_{k=1}^{N_{\text{iter}}}[\operatorname{se}(\hat{\beta}^{(k)})]^2 - \frac{1}{N_{\text{iter}}}\sum_{k=1}^{N_{\text{iter}}}[\hat{\beta}^{(k)} - \hat{\beta}]^2} \quad \text{(S51)}$$

Here, $\operatorname{se}(\hat{\beta}^{(k)})$ denotes the standard error reported by the MR method in iteration k.

Estimating correlation

We illustrate how the correlation parameter $\lambda = \frac{n_{\text{overlap}}\rho}{\sqrt{n_X n_Y}}$ can be estimated using only GWAS summary statistics for a set of genome-wide genetic variants $j = 1, \ldots, N$ with available data $\{\hat{\beta}_{X_j}, \sigma_{X_j}, \hat{\beta}_{Y_j}, \sigma_{Y_j}\}$.

Assuming that $(\hat{\beta}_{X_j}, \hat{\beta}_{Y_j})$ follows a bivariate normal distribution with mean $(\beta_{X_j}, \beta_{Y_j})$ and covariance matrix:

$$\begin{pmatrix} \sigma_{X_j}^2 & \lambda\sigma_{X_j}\sigma_{Y_j} \\ \lambda\sigma_{X_j}\sigma_{Y_j} & \sigma_{Y_j}^2 \end{pmatrix} \tag{S52}$$

the joint density function for variant j is:

$$f\left(\hat{\beta}_{X_j}, \hat{\beta}_{Y_j}\right) = \frac{1}{2\pi\sqrt{1-\lambda^2}\sigma_{X_j}\sigma_{Y_j}} \exp\left\{-\frac{1}{2(1-\lambda^2)}\left[\left(\frac{\hat{\beta}_{X_j}-\beta_{X_j}}{\sigma_{X_j}}\right)^2 - 2\lambda\left(\frac{\hat{\beta}_{X_j}-\beta_{X_j}}{\sigma_{X_j}}\right)\left(\frac{\hat{\beta}_{Y_j}-\beta_{Y_j}}{\sigma_{Y_j}}\right) + \left(\frac{\hat{\beta}_{Y_j}-\beta_{Y_j}}{\sigma_{Y_j}}\right)^2\right]\right\} \tag{S53}$$

This leads to the following log-likelihood for λ, assuming known β_{X_j}, β_{Y_j}:

$$l(\lambda) = -N\log(2\pi) - \frac{N}{2}\log(1-\lambda^2) - \log\left(\prod_{j=1}^{N}\sigma_{X_j}\sigma_{Y_j}\right) - \frac{1}{2(1-\lambda^2)}\sum_{j=1}^{N}\left[z_{X_j}^2 - 2\lambda z_{X_j}z_{Y_j} + z_{Y_j}^2\right] \tag{S54}$$

where $z_{X_j} = \frac{\hat{\beta}_{X_j}-\beta_{X_j}}{\sigma_{X_j}}$, and similarly for z_{Y_j}.

Since β_{X_j} and β_{Y_j} are generally unknown, we restrict attention to a subset of variants for which $\beta_{X_j} = \beta_{Y_j} = 0$ is a plausible assumption – i.e. variants with small effect sizes. These are selected based on having both $\left|z_{X_j}\right| < 0.5$ and $\left|z_{Y_j}\right| < 0.5$, yielding a subset of N_{sub} variants.

For this subset, the conditional log-likelihood becomes:

$$l(\lambda) = -\frac{1}{2(1-\lambda^2)}\sum_{j=1}^{N_{\text{sub}}}\left[z_{X_j}^2 - 2\lambda z_{X_j}z_{Y_j} + z_{Y_j}^2\right] - N_{\text{sub}}\log\left(\int_{-0.5}^{0.5}\int_{-0.5}^{0.5}\frac{1}{2\pi\sqrt{1-\lambda^2}}\exp\left\{-\frac{1}{2(1-\lambda^2)}\left[x^2 - 2\lambda xy + y^2\right]\right\}dx\,dy\right) \tag{S55}$$

This integral represents the probability of selecting variants with small z-scores under the bivariate normal model, and must be evaluated numerically. The log-likelihood can be maximized using numerical integration (e.g. integral2 from the R package pracma) and optimization (e.g. optimize in R) to obtain the MLE of λ.

Variant pre-filtering

To reduce computational burden in MR-SimSS, we pre-filter variants based on their probability of selection under the sample splitting scheme. At each iteration, SNP j is selected if the simulated value $|z_j^1|$ exceeds the significance threshold $\Phi^{-1}\left(1-\frac{\alpha}{2}\right)$, in which $\Phi(\cdot)$ is the standard normal cumulative

distribution function and α is the chosen p-value significance level. As a default, we use the common genome-wide significance level $\alpha = 5 \times 10^{-8}$, which gives a corresponding threshold of approximately $\Phi^{-1}\left(1 - \frac{5\times10^{-8}}{2}\right) \approx 5.45$. However, this threshold should be reduced if necessary to increase the number of instruments at each iteration.

Assuming $\hat{\sigma}_{X_j}^1 \approx \sqrt{1/\pi_1} \cdot \sigma_{X_j}$, we define:

$$z_j^1 = \frac{\hat{\beta}_{X_j}^1}{\sqrt{1/\pi_1} \cdot \sigma_{X_j}}, \qquad z_j = \frac{\hat{\beta}_{X_j}}{\sqrt{1/\pi_1} \cdot \sigma_{X_j}} \tag{S56}$$

Using previous derivations, $Z_j^1 | \hat{\beta}_{X_j} \sim N\left(z_j, 1 - \pi_1\right)$. Thus, the selection probability is:

$$P\left(\left|Z_j^1\right| > \Phi^{-1}\left(1 - \frac{\alpha}{2}\right) | z_j\right) = \Phi\left(\frac{-\Phi^{-1}\left(1 - \frac{\alpha}{2}\right) + z_j}{\sqrt{1 - \pi_1}}\right) + \Phi\left(\frac{-\Phi^{-1}\left(1 - \frac{\alpha}{2}\right) - z_j}{\sqrt{1 - \pi_1}}\right) \tag{S57}$$

This probability can be used to exclude variants with negligible likelihood of being selected.

After computing $P\left(\left|Z_j^1\right| > \Phi^{-1}\left(1 - \frac{\alpha}{2}\right) | z_j\right)$ for all variants $j = 1, \dots, N$, we construct a reduced variant set by imposing a lower bound on the probability that the restricted and full versions of MR-SimSS select the same instruments.

Variants are first ranked in ascending order of their selection probabilities. Therefore, let $p_j = P\left(\left|Z_j^1\right| > \Phi^{-1}\left(1 - \frac{\alpha}{2}\right) | z_j\right)$ and without loss of generality, assume variants are ordered by p_j, i.e. $p_1 < p_2 < \cdots < p_N$. Then,

$$\begin{aligned} &P(\text{a variant from } \{1, \dots, k\} \text{ is selected on first split if all variants are candidate instruments}) \\ &= P\left(\bigcup_{j=1}^k \left(\left|Z_j^1\right| > \Phi^{-1}\left(1 - \frac{\alpha}{2}\right)\right) | z_1, \dots, z_k\right) < \sum_{j=1}^k P\left(\left|Z_j^1\right| > \Phi^{-1}\left(1 - \frac{\alpha}{2}\right) | z_j\right) \end{aligned} \tag{S58}$$

Setting k such that the above sum in Equation (S58) is less than 0.05, the complement probability that the set of instruments selected on the first split from the candidate instruments $\{1, \dots, N\} \backslash \{1, \dots, k\}$ (i.e. the restricted version of MR-SimSS) agrees with the instruments selected from $\{1, \dots, N\}$ (i.e. the full version of MR-SimSS using all variants) under a scenario where the two simulations are "coupled" (i.e. the same split one z-statistics are simulated for the variants in the set $\{1, \dots, N\} \backslash \{1, \dots, k\}$ in both simulations) must be at least 0.95.

Therefore, for each variant, the cumulative sum is computed and all variants with cumulative selection probability exceeding 0.05 are subsequently retained. This restricts the variant set used in MR-SimSS while maintaining a high probability (95%) of selecting variants that would have been chosen from the full set. Empirically, this results in negligible impact on causal estimates but substantially reduces runtime.
