# Supplementary material for: Simulated sample splitting approach to address biases due to instrument selection and participant overlap in two-sample Mendelian Randomization studies
Source: PLoS Genet. 2026 May 8;22(5):e1011949. doi: 10.1371/journal.pgen.1011949 (PMC13178975; doi:10.1371/journal.pgen.1011949)
Supplement: Attachment — Submitted filename: review.pdf [file pgen.1011949.s002.pdf]

Referee report: PGENETICS-D-25-01198

This paper proposes a simple yet effective way to address selection bias due to instrument selection in two-sample Mendelian randomization. The main idea is as follows (with some paraphrasing). Consider the usual measurement error model for two-sample MR:

$$\begin{pmatrix}\hat\beta_{Xj}\\ \hat\beta_{Yj}\end{pmatrix} \sim \mathrm{N}\left(\begin{pmatrix}\beta_{Xj}\\ \beta_{Yj}\end{pmatrix}, \begin{pmatrix}\sigma_{Xj}^2 & \lambda\sigma_{Xj}\sigma_{Yj}\\ \lambda\sigma_{Xj}\sigma_{Yj} & \sigma_{Yj}^2\end{pmatrix}\right)$$

Practitioners often select a genetic instrument (indexed by j) if $|\hat\beta_{Xj}/\sigma_{Xj}|$ is large and use the same $\hat\beta_{Xj}$ for statistical inference. This results in selection bias, as $\hat\beta_{Xj}$ no longer follows a normal distribution and is larger than β_{Xj} on average after selection. The authors' main idea is to include an additional random variable

$$Z_{Xj} \sim \mathrm{N}(0, \frac{1-\pi}{\pi}\sigma_{Xj}^2)$$

and consider

$$\hat\beta_{Xj}^1 = \hat\beta_{Xj} + Z_{Xj}, \quad \hat\beta_{Xj}^2 = \hat\beta_{Xj} - \frac{\pi}{1-\pi}Z_{Xj} = \frac{\hat\beta_{Xj} - \pi\hat\beta_{Xj}^1}{1-\pi},$$

so we have

$$\begin{pmatrix}\hat\beta_{Xj}^1\\ \hat\beta_{Xj}^2\\ \hat\beta_{Yj}\end{pmatrix} \sim \mathrm{N}\left(\begin{pmatrix}\beta_{Xj}\\ \beta_{Xj}\\ \beta_{Yj}\end{pmatrix}, \begin{pmatrix}\frac{1}{\pi}\sigma_{Xj}^2 & 0 & \lambda\sigma_{Xj}\sigma_{Yj}\\ 0 & \frac{1}{1-\pi}\sigma_{Xj}^2 & \lambda\sigma_{Xj}\sigma_{Yj}\\ \lambda\sigma_{Xj}\sigma_{Yj} & \lambda\sigma_{Xj}\sigma_{Yj} & \sigma_{Yj}^2\end{pmatrix}\right)$$

If $\lambda = 0$ (no sample overlap), this essentially creates a standard three-sample MR study for which $\hat\beta_{Xj}^1$ can be used for instrument selection and $(\hat\beta_{Xj}^2, \hat\beta_{Yj})$ can be used for statistical inference using standard two-sample MR methods.

1 Major comments

My main comments are as follows:

1. The authors described the "orthogonal randomization" trick outlined above as "simulated sample splitting". Although these are just equivalent ways of describing the same trick, compared to equation (1) in the paper I think it is mathematically much cleaner to say the method is simply injecting noise to the variant-exposure summary data to create an artificial three-sample design. This way the proof is straightforward and doesn't require a Supplementary Note.

2. A major limitation of this method is that it assumes "linkage disequilibrium (LD) pruning or clumping has already been applied, resulting in a set of uncorrelated variants with summary statistics available in both GWASs". However, one needs to use the variant-exposure GWAS to perform LD clumping, so the normal measurement error model is not correct after LD clumping. Fortunately, I think there is a simple modification that saves the day. Instead of assuming the genetic instruments are independent, we can start with correlated variants and it is not difficult to show that $\mathrm{Cor}(\hat{\beta}_{Xj}, \hat{\beta}_{Xk})$ is the same as the Pearson correlation between variant j and variant k, which can be estimated using a reference panel. Now instead of injecting independent noise Z_{Xj} and Z_{Xk}, we can make $\mathrm{Cor}(Z_{Xj}, Z_{Xk}) = \mathrm{Cor}(\hat{\beta}_{Xj}, \hat{\beta}_{Xk})$. With some simple algebra it can be shown that $\mathrm{Cor}(\hat{\beta}^1_{Xj}, \hat{\beta}^2_{Xk}) = 0$, so $(\hat{\beta}^1_{Xj}, \hat{\beta}^1_{Xk})$ is independent of $(\hat{\beta}^2_{Xj}, \hat{\beta}^2_{Xk})$. One can then apply LD clumping to $(\hat{\beta}^1_{Xj})$ without incurring selection bias on $(\hat{\beta}^2_{Xk})$. In practice, this means that one need to find a way to sample a Gaussian process (or practically a high-dimensional Gaussian vector) Z_{Xj} with covariance function given by the LD matrix. LLMs suggest that this can be done using block-diagonal approximation to the LD matrix and packages like simGWAS and GWASBrewer.

3. I was not able to decipher how the authors address sample overlap by the 3-split framework, which further "split"/add noise to $\hat{\beta}^2_{Xj}$. The authors wrote "These are simulated using a second conditional distribution, analogous to the form above." But more details are needed and I cannot find the exact formula for this in the Supplementary Note. Note that sample overlap is not necessarily a problem for two-sample summary-data MR: a solution to this is given in MR-GRAPPLE (an extension to MR-RAPS, see `https://doi.org/10.1371/journal.pgen.1009575`), which also includes a way of estimating the parameter λ that is similar to the proposal in the Supplementary Note of this paper. What's not so clear is that $\hat{\beta}^1_{Xj}$ and $\hat{\beta}_{Yj}$ are

correlated (see my formula above), so selecting on $\hat{\beta}^1_{Xj}$ changes the distribution of $\hat{\beta}_{Yj}$. But I think this can be worked out by adding noise to $\hat{\beta}_{Yj}$:

$$\hat{\beta}^1_{Yj} = \hat{\beta}_{Yj} - Z_{Yj}, \quad \hat{\beta}^2_{Yj} = \hat{\beta}_{Yj} + \frac{1-\pi}{\pi} Z_{Yj},$$

where $\mathrm{Cor}(Z_{Xj}, Z_{Yj}) = \lambda$ and $\mathrm{Var}(Z_{Yj}) = \frac{\pi}{1-\pi}\sigma^2_{Yj}$. With some algebra, it can be shown that

$$\begin{pmatrix} \hat{\beta}^1_{Xj} \\ \hat{\beta}^2_{Xj} \\ \hat{\beta}^1_{Yj} \\ \hat{\beta}^2_{Yj} \end{pmatrix} \sim \mathrm{N}\left(\begin{pmatrix} \beta_{Xj} \\ \beta_{Xj} \\ \beta_{Yj} \\ \beta_{Yj} \end{pmatrix}, \begin{pmatrix} \frac{1}{\pi}\sigma^2_{Xj} & 0 & 0 & \frac{\lambda}{\pi}\sigma_{Xj}\sigma_{Yj} \\ 0 & \frac{1}{1-\pi}\sigma^2_{Xj} & \frac{\lambda}{1-\pi}\sigma_{Xj}\sigma_{Yj} & 0 \\ 0 & \frac{\lambda}{1-\pi}\sigma_{Xj}\sigma_{Yj} & \frac{1}{1-\pi}\sigma^2_{Yj} & 0 \\ \frac{\lambda}{\pi}\sigma_{Xj}\sigma_{Yj} & 0 & 0 & \frac{1}{\pi}\sigma^2_{Yj} \end{pmatrix} \right).$$

So one can use $(\hat{\beta}^1_{Xj})$ for instrument selection (with LD clumping, see point 2 above) and then MR-GRAPPLE with $(\hat{\beta}^2_{Xj}, \hat{\beta}^1_{Yj})$. Note that we cannot use standard methods (like IVW) on $(\hat{\beta}^2_{Xj}, \hat{\beta}^2_{Yj})$ (even though they are independent) because selecting on $(\hat{\beta}^1_{Xj})$ changes the distribution of $\hat{\beta}^2_{Yj}$.

2 Minor comments

1. The idea of selecting instruments after injecting some noise is also suggested in a previous paper (`https://doi.org/10.1214/22-AOS2247`). But that paper did not use orthogonalization to create independent data for inference and did not consider selection by LD clumping (so suffers from my same criticism in point 2 above).

2. Do the authors have plans to implement this in/integrate this with TwoSampleMR (after hopefully taking in my suggestions above)?

3. I did not get the "probabilistic variant pre-selection" part. It looks like a deterministic selection procedure so I have doubts about whether it can be justified.

4. The authors should compare their real data results in "Effect of body mass index on type 2 diabetes" with true three-sample estimates.

5. Line 61: "most MR analyses now use a sample, or summary-level, design." Confusing, why "or"?

6. Line 83: "A genetic variant is considered a weak instrument if it explains only a small proportion of variation in the exposure". This is not quite right. "Weakness" is also relative to the sample size.

7. Line 86: "In a complete overlap (one-sample) setting, causal estimates are biased toward the observational association, increasing the risk of Type I error." The term "Type I error" is from hypothesis testing and is ambiguous in this setting about causality. I would say something like "... are biased toward the observational association, which may increase false positive findings".
