# Supplementary material for: Simulated sample splitting approach to address biases due to instrument selection and participant overlap in two-sample Mendelian Randomization studies
Source: PLoS Genet. 2026 May 8;22(5):e1011949. doi: 10.1371/journal.pgen.1011949 (PMC13178975; doi:10.1371/journal.pgen.1011949)
Supplement: Attachment — Submitted filename: Response-to-Reviewers.pdf [file pgen.1011949.s004.pdf]

Amanda Forde
School of Mathematical and Statistical Sciences
University of Galway
Galway
Ireland
Email: amanda.forde@universityofgalway.ie
Monday, 2nd March 2026

Dear Editors,

We are pleased to present a revised version of our manuscript "Simulated sample splitting approach to address biases due to instrument selection and participant overlap in two-sample Mendelian Randomization studies".

We thank the reviewers for their thoughtful, detailed, and constructive comments, which have substantially improved the quality of the manuscript. We have carefully considered each point raised and provide a detailed, point-by-point response to all comments below. We look forward to hearing back from you soon.

Yours sincerely,
Amanda Forde

Reviewer #1:

This is a creative and timely solution to the winner's curse problem in MR. I enjoyed reading the paper, and have only minor comments:

1. **Table 1 basically shows no bias in any method except dIVW, which isn't very useful. I suppose it's because it averages over situations with different degrees of overlap. It may be more informative to break this table down a bit, with full results still in supplement.**
   We thank the reviewer for this comment – they are correct. The average bias reported previously was not that informative since it could be low even when there was substantial bias at every combination of parameter settings, if positive and negative biases cancelled out over different parameter settings (or simulation scenarios). This happened in the previous Table 1. To reflect this, we have now included 'average absolute bias' as an additional column in the table, since absolute bias can't average to 0 over differing scenarios. We have also now restricted Table 1 to refer to a scenario where the exposure-outcome correlation is 0.5, which reduces the issue of positive and negative biases cancelling, although we still average over differing scenarios regarding heritability and proportion of 'true-effect' variants. Completely aggregated results, and results for differing values of the correlation are given in the Supplementary Note. As one can see in the new Table 1 (as well as in the completely aggregated results in the Supplementary Note), average absolute bias is minimised for SimSS-3-RAPS. We have also slightly reworded the Results Section with more focus given to independent overlap-correlation configurations, to avoid confusion with respect to biases cancelling each other out.

2. **The dIVW results are not really comparable with the others because it is using the full set of 1M SNPs. I appreciate that it needs a large number of SNPs to get a good bias adjustment, but this might explain some of the differences from other methods. MR-RAPS can also work with a large number of SNPs, so if you want to consider whole-genome instruments, you could perhaps compare dIVW to MR-RAPS (others have found them to be very similar).**
   We thank the reviewer for this comment. On reflection, we agree and therefore, we have removed dIVW from our simulation study. Note that the simulation results in the original manuscript showed that dIVW is highly sensitive to sample overlap. However, they also showed that dIVW can provide unbiased estimates in settings with non-overlapping samples. For this reason, we feel that dIVW should still be included in the same-trait empirical analyses as it can act as a useful benchmark to compare MR-SimSS and other methods against.

3. **Supp p17, "By standard linear model theory.." - var(X) should be the residual variance (which is approx var(X) when SNPs have small effects). Also, the normal distribution is asymptotic.**

We agree with this comment – unless X is normally distribution, the stated distribution is asymptotic. We have rephrased the statement as follows:
"From standard asymptotic theory (assuming the linear model is correct, observations are independent and individual variants explain only a small fraction of the variation in $\boldsymbol{X}$), $\widehat{\boldsymbol{\beta}}_X \sim N(\boldsymbol{\beta}_X, \mathrm{var}(X)(\boldsymbol{G}_X^{\mathrm{T}}\boldsymbol{G}_X)^{-\mathbf{1}})$."

4. **Supp p18, below eq S9 the sentence beginning "Hence, " is not rigorous - I know what you mean, but it's illogical to define C as approximately equal to something.**
   To address this comment, the sentence below eq S9 now reads "Letting $\mathbf{C} = \mathrm{n_X}\left(\mathbb{E}\left[\mathbf{G}_\mathbf{X}^{\mathrm{T}}\mathbf{G}_\mathbf{X}\right]\right)^{-\mathbf{1}} = \mathrm{n_Y}\left(\mathbb{E}\left[\mathbf{G}_\mathbf{Y}^{\mathrm{T}}\mathbf{G}_\mathbf{Y}\right]\right)^{-\mathbf{1}}$ allows us…", while S6 has been amended to:
   "Applying the law of large numbers, we note: $\mathbb{E}\left[\mathbf{G}_\mathbf{X}^{\mathrm{T}}\mathbf{G}_\mathbf{X}\right] \approx \mathbf{G}_\mathbf{X}^{\mathrm{T}}\mathbf{G}_\mathbf{X} \approx \cdots$ (S6)"

5. **Supp p19, eq S19 the 4th expression should be var instead of cov? (can't have covariance of one variable)**
   The 4th expression should be var instead of cov, it has been changed accordingly.

6. **Supp p20, above eq S24, "For binary Y..." there is no error term in the logistic regression equation.**
   We thank the reviewer for noticing this mistake. The error term has now been removed.

7. **Supp p21 first line, is the W on the RHS correct?**
   The W on the RHS is incorrect – it is a typo which has now been removed.

Reviewer #2:

This paper proposes a simple yet effective way to address selection bias due to instrument selection in two-sample Mendelian randomization. The main idea is as follows (with some paraphrasing). Consider the usual measurement error model for two-sample MR:

$$\begin{pmatrix} \hat{\beta}_{X_j} \\ \hat{\beta}_{Y_j} \end{pmatrix} \sim N\left(\begin{pmatrix} \beta_{X_j} \\ \beta_{Y_j} \end{pmatrix}, \begin{pmatrix} \sigma_{X_j}^2 & \lambda\sigma_{X_j}\sigma_{Y_j} \\ \lambda\sigma_{X_j}\sigma_{Y_j} & \sigma_{Y_j}^2 \end{pmatrix} \right)$$

Practitioners often select a genetic instrument (indexed by j) if $\left|\widehat{\boldsymbol{\beta}}_{X_j}/\boldsymbol{\sigma}_{X_j}\right|$ is large and use the same $\widehat{\boldsymbol{\beta}}_{X_j}$ for statistical inference. This results in selection bias, as $\widehat{\boldsymbol{\beta}}_{X_j}$ no longer follows a normal distribution and is larger than $\boldsymbol{\beta}_{X_j}$ on average after selection. The authors' main idea is to include an additional random variable

$$Z_{X_j} \sim N\left(0, \frac{1-\pi}{\pi}\sigma_{X_j}^2\right)$$

and consider

$$\hat{\beta}^1_{X_j} = \hat{\beta}_{X_j} + Z_{X_j}, \qquad \hat{\beta}^2_{X_j} = \hat{\beta}_{X_j} - \frac{\pi}{1-\pi} Z_{X_j} = \frac{\hat{\beta}_{X_j} - \pi\hat{\beta}^1_{X_j}}{1-\pi}$$

so we have

$$\begin{pmatrix} \hat{\beta}^1_{X_j} \\ \hat{\beta}^2_{X_j} \\ \hat{\beta}_{Y_j} \end{pmatrix} \sim N\left(\begin{pmatrix} \beta_{X_j} \\ \beta_{X_j} \\ \beta_{Y_j} \end{pmatrix}, \begin{pmatrix} \frac{1}{\pi}\sigma^2_{X_j} & 0 & \lambda\sigma_{X_j}\sigma_{Y_j} \\ 0 & \frac{1}{1-\pi}\sigma^2_{X_j} & \lambda\sigma_{X_j}\sigma_{Y_j} \\ \lambda\sigma_{X_j}\sigma_{Y_j} & \lambda\sigma_{X_j}\sigma_{Y_j} & \sigma^2_{Y_j} \end{pmatrix} \right)$$

If $\lambda = 0$ (no sample overlap), this essentially creates a standard three-sample MR study for which $\widehat{\boldsymbol{\beta}}^1_{X_j}$ can be used for instrument selection and $\left(\widehat{\boldsymbol{\beta}}^2_{X_j}, \widehat{\boldsymbol{\beta}}_{Y_j}\right)$ can be used for statistical inference using standard two-sample MR methods.

My major comments are as follows:

1. The authors described the “orthogonal randomization” trick outlined above as “simulated sample splitting”. Although these are just equivalent ways of describing the same trick, compared to equation (1) in the paper I think it is mathematically much cleaner to say the method is simply injecting noise to the variant-exposure summary data to create an artificial three-sample design. This way the proof is straightforward and doesn’t require a Supplementary Note.

3. I was not able to decipher how the authors address sample overlap by the 3-split framework, which further “split”/ add noise to $\widehat{\boldsymbol{\beta}}^2_{X_j}$. The authors wrote “These are simulated using a second conditional distribution, analogous to the form above”. But more details are needed and I cannot find the exact formula for this in the Supplementary Note. Note that sample overlap is not necessarily a problem for two-sample summary-data MR: a solution to this is given in MR-GRAPPLE (an extension to MR-RAPS, see https://doi.org/10.1371/journal.pgen.1009575), which also includes a way of estimating the parameter λ that is similar to the proposal in the Supplementary Note of this paper. What’s not so clear is that $\hat{\beta}^1_{X_j}$ and $\hat{\beta}_{Y_j}$ are correlated (see my formula above), so selecting on $\hat{\beta}^1_{X_j}$ changes the distribution of $\hat{\beta}_{Y_j}$. But I think this can be worked out by adding noise to $\hat{\beta}_{Y_j}$:

$$\hat{\beta}^1_{Y_j} = \hat{\beta}_{Y_j} - Z_{Y_j}, \qquad \hat{\beta}^2_{Y_j} = \hat{\beta}_{Y_j} + \frac{1-\pi}{\pi} Z_{Y_j}$$

where $\mathrm{cor}\left(Z_{X_j}, Z_{Y_j}\right) = \lambda$ and $\mathrm{var}\left(Z_{Y_j}\right) = \frac{\pi}{1-\pi}\sigma^2_{Y_j}$. With some algebra, it can be shown that:

$$\begin{pmatrix} \hat{\beta}^1_{X_j} \\ \hat{\beta}^2_{X_j} \\ \hat{\beta}^1_{Y_j} \\ \hat{\beta}^2_{Y_j} \end{pmatrix} \sim N\left(\begin{pmatrix} \beta_{X_j} \\ \beta_{X_j} \\ \beta_{Y_j} \\ \beta_{Y_j} \end{pmatrix}, \begin{pmatrix} \frac{1}{\pi}\sigma^2_{X_j} & 0 & 0 & \frac{\lambda}{\pi}\sigma_{X_j}\sigma_{Y_j} \\ 0 & \frac{1}{1-\pi}\sigma^2_{X_j} & \frac{\lambda}{1-\pi}\sigma_{X_j}\sigma_{Y_j} & 0 \\ 0 & \frac{\lambda}{1-\pi}\sigma_{X_j}\sigma_{Y_j} & \frac{1}{1-\pi}\sigma^2_{Y_j} & 0 \\ \frac{\lambda}{\pi}\sigma_{X_j}\sigma_{Y_j} & 0 & 0 & \frac{1}{\pi}\sigma^2_{Y_j} \end{pmatrix} \right)$$

So one can use $\left(\hat{\beta}^1_{X_j}\right)$ for instrument selection (with LD clumping, see point 2 above) and then MR-GRAPPLE with $\left(\hat{\beta}^2_{X_j}, \ \hat{\beta}^1_{Y_j}\right)$. Note that we cannot use standard methods (like IVW) on $\left(\hat{\beta}^2_{X_j}, \ \hat{\beta}^2_{Y_j}\right)$ (even though they are independent) because selecting on $\left(\hat{\beta}^1_{X_j}\right)$ changes the distribution of $\hat{\beta}^2_{Y_j}$.

We thank the reviewer for both of the above comments and for providing an alternative description of our proposed method via "inserting randomness". For clarity, in what follows, we will re-describe our method in a similar format to that of the reviewer.

For a given genetic variant j, we consider the following model, in which variant-exposure and variant-outcome association estimates are denoted by $\hat{\beta}_{X_j}$ and $\hat{\beta}_{Y_j}$ respectively:

$$\begin{pmatrix} \hat{\beta}_{X_j} \\ \hat{\beta}_{Y_j} \end{pmatrix} \sim N\left(\begin{pmatrix} \beta_{X_j} \\ \beta_{Y_j} \end{pmatrix}, \begin{pmatrix} \sigma^2_{X_j} & \lambda\sigma_{X_j}\sigma_{Y_j} \\ \lambda\sigma_{X_j}\sigma_{Y_j} & \sigma^2_{Y_j} \end{pmatrix} \right)$$

Here, $\lambda = \frac{n_{\text{overlap}}\rho}{\sqrt{n_X n_Y}}$ in which $\rho = \text{cor}(X, Y)$ denotes the correlation between the exposure and outcome, potentially non-zero due to confounding and/or obtaining association estimates from overlapping samples. In order to eliminate Winner's Curse, we simulate a splitting process (random splitting of individual-level data into two fractions π_1 and $1 - \pi_1$) by drawing from our derived asymptotic conditional distribution of the GWAS estimates in the π_1-fraction, conditional on the full-sample GWAS statistics (see Equation (1) in main manuscript). Instruments are then selected using z-statistics $z^1_j = \frac{\hat{\beta}^1_{X_j}}{\sigma^1_{X_j}}$. Association estimates in the remaining $(1 - \pi_1)$-fraction can be reconstructed as: $\hat{\beta}^2_{X_j} \approx \frac{\hat{\beta}_{X_j} - \pi_1\hat{\beta}^1_{X_j}}{1-\pi_1}$ and $\hat{\beta}^2_{Y_j} \approx \frac{\hat{\beta}_{Y_j} - \pi_1\hat{\beta}^1_{Y_j}}{1-\pi_1}$. It is these association estimates that are then inputted into the summary-level MR method, such as IVW. This describes the 2-split implementation of our proposed method, MR-SimSS.

In alignment with the reviewer's comments, our main idea can also be described as follows. We introduce an additional bivariate random vector with correlated components:

$$\mathbf{U} = \begin{pmatrix} U_{X_j} \\ U_{Y_j} \end{pmatrix} \sim N\left(\begin{pmatrix} 0 \\ 0 \end{pmatrix}, \frac{1-\pi_1}{\pi_1} \begin{pmatrix} \sigma^2_{X_j} & \lambda\sigma_{X_j}\sigma_{Y_j} \\ \lambda\sigma_{X_j}\sigma_{Y_j} & \sigma^2_{Y_j} \end{pmatrix} \right)$$

and consider:

$$\begin{pmatrix}\hat{\beta}^1_{X_j}\\ \hat{\beta}^1_{Y_j}\end{pmatrix} = \begin{pmatrix}\hat{\beta}_{X_j}\\ \hat{\beta}_{Y_j}\end{pmatrix} + \begin{pmatrix}U_{X_j}\\ U_{Y_j}\end{pmatrix}, \quad \begin{pmatrix}\hat{\beta}^2_{X_j}\\ \hat{\beta}^2_{Y_j}\end{pmatrix} = \begin{pmatrix}\hat{\beta}_{X_j}\\ \hat{\beta}_{Y_j}\end{pmatrix} - \frac{\pi_1}{1-\pi_1}\begin{pmatrix}U_{X_j}\\ U_{Y_j}\end{pmatrix} = \frac{1}{1-\pi_1}\begin{bmatrix}\hat{\beta}_{X_j} - \pi_1\hat{\beta}^1_{X_j}\\ \hat{\beta}_{Y_j} - \pi_1\hat{\beta}^1_{Y_j}\end{bmatrix}$$

The above line is equivalent to Equation (2) in the manuscript. It can then be shown that we have the following joint distribution:

$$\begin{pmatrix}\hat{\beta}^1_{X_j}\\ \hat{\beta}^1_{Y_j}\\ \hat{\beta}^2_{X_j}\\ \hat{\beta}^2_{Y_j}\end{pmatrix} \sim N\left(\begin{pmatrix}\beta_{X_j}\\ \beta_{Y_j}\\ \beta_{X_j}\\ \beta_{Y_j}\end{pmatrix}, \begin{pmatrix}\frac{1}{\pi_1}\sigma^2_{X_j} & \frac{\lambda}{\pi_1}\sigma_{X_j}\sigma_{Y_j} & 0 & 0\\ \frac{\lambda}{\pi_1}\sigma_{X_j}\sigma_{Y_j} & \frac{1}{\pi_1}\sigma^2_{Y_j} & 0 & 0\\ 0 & 0 & \frac{1}{1-\pi_1}\sigma^2_{X_j} & \frac{\lambda}{1-\pi_1}\sigma_{X_j}\sigma_{Y_j}\\ 0 & 0 & \frac{\lambda}{1-\pi_1}\sigma_{X_j}\sigma_{Y_j} & \frac{1}{1-\pi_1}\sigma^2_{Y_j}\end{pmatrix}\right)$$

If $\lambda = 0$, this essentially creates a standard three-sample MR study for which $\hat{\beta}^1_{X_j}$ can be used for instrument selection and $\left(\hat{\beta}^2_{X_j},\ \hat{\beta}^2_{Y_j}\right)$ can be used for statistical inference using standard two-sample MR methods. We note that this differs from the interpretation of our approach provided by the reviewer, and we also note the difference in how we have defined $\left(\hat{\beta}^1_{Y_j},\ \hat{\beta}^2_{Y_j}\right)$ versus the reviewer's definition given in comment #3.

However, our 2-split approach may still incur weak instrument bias in the direction of confounding if the exposure and outcome GWASs overlap *(* $\lambda \neq 0$*)*. To address this, we propose extending the approach to a 3-split framework in which the $(1-\pi_1)$-fraction is further conceptually split into sub-fractions of relative sizes π_2 and $1-\pi_2$, with simulated exposure estimates derived from the π_2-subset and outcome estimates from the remaining $(1-\pi_2)$-subset. In the manuscript, we state that "these are simulated using a second conditional distribution, analogous to the form above". We will elaborate on this statement to provide a clearer description of this second-splitting process. In a similar manner to the first-split, we begin by drawing from our derived asymptotic conditional distribution of the GWAS estimates in the π_2-fraction, conditional on the $(1-\pi_1)$-fraction GWAS statistics (see Equation (S24) in the revised Supplementary Note). Thus, for each variant j, association estimates $\hat{\beta}^{2\mathrm{a}}_{X_j}$ and $\hat{\beta}^{2\mathrm{b}}_{Y_j}$ (termed as $\hat{\beta}^{(1-\pi)p}_{X_j}$ and $\hat{\beta}^{(1-\pi)p}_{Y_j}$ in the original manuscript) are simulated according to:

$$\begin{pmatrix}\hat{\beta}^{2\mathrm{a}}_{X_j}\\ \hat{\beta}^{2\mathrm{a}}_{Y_j}\end{pmatrix} \Big| \begin{pmatrix}\hat{\beta}^2_{X_j}\\ \hat{\beta}^2_{Y_j}\end{pmatrix} \sim N\left(\begin{pmatrix}\hat{\beta}^2_{X_j}\\ \hat{\beta}^2_{Y_j}\end{pmatrix}, \frac{1-\pi_2}{\pi_2}\cdot\frac{1}{1-\pi_1}\begin{pmatrix}\sigma^2_{X_j} & \lambda\sigma_{X_j}\sigma_{Y_j}\\ \lambda\sigma_{X_j}\sigma_{Y_j} & \sigma^2_{Y_j}\end{pmatrix}\right)$$

Variant-outcome association estimates in the remaining $(1-\pi_2)$-fraction can be reconstructed as: $\hat{\beta}^{2\mathrm{b}}_{Y_j} \approx \frac{\hat{\beta}^2_{Y_j} - \pi_2\hat{\beta}^{2\mathrm{a}}_{Y_j}}{1-\pi_2}$. It is then the association estimates $\left(\hat{\beta}^{2\mathrm{a}}_{X_j},\ \hat{\beta}^{2\mathrm{b}}_{Y_j}\right)$ that are inputted into the summary-level MR method. Even in cases in which $\lambda \neq 0$, this simulated 3-split design is equivalent to the standard three-sample MR study for which $\hat{\beta}^1_{X_j}$ is used for

instrument selection and $\left(\hat{\beta}_{X_j}^{2a},\ \hat{\beta}_{Y_j}^{2b}\right)$ are used for estimation with standard two-sample MR methods.

This additional split can also be explained as follows. In addition to $\mathbf{U}$, another bivariate random vector is introduced with components:

$$\mathbf{V} = \begin{pmatrix} V_{X_j} \\ V_{Y_j} \end{pmatrix} \sim N\left(\begin{pmatrix} 0 \\ 0 \end{pmatrix}, \frac{1-\pi_2}{\pi_2}\begin{pmatrix} \sigma_{X_j}^2 & \lambda\sigma_{X_j}\sigma_{Y_j} \\ \lambda\sigma_{X_j}\sigma_{Y_j} & \sigma_{Y_j}^2 \end{pmatrix}\right)$$

and we then consider:

$$\begin{pmatrix} \hat{\beta}_{X_j}^{2a} \\ \hat{\beta}_{Y_j}^{2a} \end{pmatrix} = \begin{pmatrix} \hat{\beta}_{X_j}^{2} \\ \hat{\beta}_{Y_j}^{2} \end{pmatrix} + \begin{pmatrix} V_{X_j} \\ V_{Y_j} \end{pmatrix}, \qquad \begin{pmatrix} \hat{\beta}_{X_j}^{2b} \\ \hat{\beta}_{Y_j}^{2b} \end{pmatrix} = \begin{pmatrix} \hat{\beta}_{X_j}^{2} \\ \hat{\beta}_{Y_j}^{2} \end{pmatrix} - \frac{\pi_2}{1-\pi_2}\begin{pmatrix} V_{X_j} \\ V_{Y_j} \end{pmatrix} = \frac{1}{1-\pi_2}\begin{bmatrix} \hat{\beta}_{X_j}^{2} - \pi_2\hat{\beta}_{X_j}^{2a} \\ \hat{\beta}_{Y_j}^{2} - \pi_2\hat{\beta}_{Y_j}^{2a} \end{bmatrix}$$

Putting all of the above together, the joint distribution of $[\hat{\beta}_{X_j}^{1}, \hat{\beta}_{Y_j}^{1}, \hat{\beta}_{X_j}^{2a}, \hat{\beta}_{Y_j}^{2a}, \hat{\beta}_{X_j}^{2a}, \hat{\beta}_{Y_j}^{2b}]^T$ can be written as:

$$\begin{pmatrix} \hat{\beta}_{X_j}^{1} \\ \hat{\beta}_{Y_j}^{1} \\ \hat{\beta}_{X_j}^{2a} \\ \hat{\beta}_{Y_j}^{2a} \\ \hat{\beta}_{X_j}^{2b} \\ \hat{\beta}_{Y_j}^{2b} \end{pmatrix} \sim N\left(\begin{pmatrix} \beta_{X_j} \\ \beta_{Y_j} \\ \beta_{X_j} \\ \beta_{Y_j} \\ \beta_{X_j} \\ \beta_{Y_j} \end{pmatrix}, \begin{pmatrix} \frac{\sigma_{X_j}^2}{\pi_1} & \frac{\lambda\sigma_{X_j}\sigma_{Y_j}}{\pi_1} & 0 & 0 & 0 & 0 \\ \frac{\lambda\sigma_{X_j}\sigma_{Y_j}}{\pi_1} & \frac{\sigma_{Y_j}^2}{\pi_1} & 0 & 0 & 0 & 0 \\ 0 & 0 & \frac{\sigma_{X_j}^2}{\pi_2(1-\pi_1)} & \frac{\lambda\sigma_{X_j}\sigma_{Y_j}}{\pi_2(1-\pi_1)} & 0 & 0 \\ 0 & 0 & \frac{\lambda\sigma_{X_j}\sigma_{Y_j}}{\pi_2(1-\pi_1)} & \frac{\sigma_{Y_j}^2}{\pi_2(1-\pi_1)} & 0 & 0 \\ 0 & 0 & 0 & 0 & \frac{\sigma_{X_j}^2}{(1-\pi_2)(1-\pi_1)} & \frac{\lambda\sigma_{X_j}\sigma_{Y_j}}{(1-\pi_2)(1-\pi_1)} \\ 0 & 0 & 0 & 0 & \frac{\lambda\sigma_{X_j}\sigma_{Y_j}}{(1-\pi_2)(1-\pi_1)} & \frac{\sigma_{Y_j}^2}{(1-\pi_2)(1-\pi_1)} \end{pmatrix}\right)$$

We agree that this description of MR-SimSS via “inserting randomness” is definitely neater mathematically. However, we feel that most people, particularly those with a non-mathematical research background, will likely understand the sample splitting analogy better. That said, given the reviewer’s comment, we have chosen to also include this alternative proof of the method, with respect to a continuous response, in the Supplementary Note. We also note that the exact formula for the second conditional distribution is now available in the Supplementary Note (see Equation (S24)) as well. Furthemore, we have acknowledged GRAPPLE’s similar approach to controlling for sample overlap in the Discussion section of our manuscript.

2. **A major limitation of this method is that it assumes "linkage disequilibrium (LD) pruning or clumping has already been applied, resulting in a set of uncorrelated variants with summary statistics available in both GWASs". However, one needs to use the variant-exposure GWAS to perform LD clumping, so the normal measurement error model is not correct after LD clumping. Fortunately, I think there is a simple modification that saves the day. Instead of assuming the genetic instruments are independent, we can start with correlated variants and it is not difficult to show that** $\mathrm{cor}\left(\hat{\beta}_{X_j},\ \hat{\beta}_{X_k}\right)$ **is the same as the Pearson correlation between variant *j* and variant *k*, which can be estimated using a reference panel. Now instead of injecting instrument noise** Z_{X_j} **and** Z_{X_k}**, we can make** $\mathrm{cor}\left(Z_{X_j},\ Z_{X_k}\right) = \mathrm{cor}\left(\hat{\beta}_{X_j},\ \hat{\beta}_{X_k}\right)$**. With some simple algebra, it can be shown that** $\mathrm{cor}\left(\hat{\beta}^{1}_{X_j},\ \hat{\beta}^{2}_{X_k}\right) = 0$ **so** $\left(\hat{\beta}^{1}_{X_j},\ \hat{\beta}^{1}_{X_k}\right)$ **is independent of** $\left(\hat{\beta}^{2}_{X_j},\ \hat{\beta}^{2}_{X_k}\right)$**. One can then apply LD clumping to** $\left(\hat{\beta}^{1}_{X_j}\right)$ **without incurring selection bias on** $\left(\hat{\beta}^{2}_{X_k}\right)$**. In practice, this means that one need to find a way to sample a Gaussian process (or practically a high-dimensional Gaussian vector)** Z_{X_j} **with covariance function given by the LD matrix. LLMs suggest that this can be done using block-diagonal approximation to the LD matrix and packages like simGWAS and GWASBrewer.**

We thank the reviewer for this clever suggestion. While we agree in theory that one could address the selection bias due to clumping via injecting correlated noise (simulated via a Gaussian process according to the true LD matrix of the genetic variants), in practice this is likely to substantially slow down the algorithm compared to simulating independent associations at each variant. Already, we have had to make some computational compromises to speed up implementation. For instance, the deterministic pre-selection of a set of variants that have a high probability of ending up as the final instruments on the first split was necessary. While that calculation (which is now justified more rigorously in the Supplementary Note than before) is still valid if the variant associations are correlated in an LD block, it likely won't result in the same computational savings as before, given that groups of genetic variants in the same LD block are likely to be significant at the same time.

In practice, we recommend running the algorithm on an LD-pruned (rather than LD-clumped) set of variants and have amended the sentence mentioned by the reviewer to reflect this recommendation, i.e. "we… assume that linkage disequilibrium (LD) pruning has already been applied, resulting in a set of uncorrelated variants". Using a set of pruned variants ensures that there is no additional selection bias induced (i.e. bias arising from retaining the most strongly exposure-associated variants in each LD region). Therefore, the normal measurement error model for the selected variants via LD-pruning is appropriate, as the distribution of the selected variant-exposure associations remains unchanged. For illustrative purposes, we have included a same-trait BMI-BMI analysis which has been performed twice – once using a set of LD-pruned variants and once using a set of LD-clumped variants (Supplementary Fig. 14, Supplementary Table 8). In order to compare like with like, LD-pruning parameters were chosen so that both sets of

variants contained a similar number of instruments (variants deemed significant according to the p-value threshold of 5×10^{-8}). This analysis shows the two MR-SimSS approaches that integrate MR-RAPS (SimSS-2-RAPS and SimSS-3-RAPS) providing unbiased causal effect estimates when used with LD-pruned variants, but not with LD-clumped variants. Due to selecting the variant with the smallest p-value in each region of the genome, the clumping process induces another form of selection bias. This leads to MR estimates obtained with clumped sets generally suffering from a greater magnitufe of bias towards the null than their pruned counterparts. This is a very important point, particularly with respect to MR-SimSS implementation, and thus, we are very grateful to the reviewer for highlighting that attention was not given to this in the original manuscript.

We now provide more detailed comments regarding the above in the Discussion section of the manuscript. Futhermore, we also include the reviewer's interesting suggestion as possible future work as it may increase the power of the algorithm and allow application of the algorithm with LD-clumped data.

Minor comments:

4. **The idea of selecting instruments after injecting some noise is also suggested in a previous paper (https://doi.org/10.1214/22-AOS2247). But that paper did not use orthogonalization to create independent data for inference and did not consider selection by LD clumping (so suffers from my same criticism in point 2 above).**
We thank the reviewer for drawing our attention to this previous paper. We mentioned this approach (RIVW) in the Discussion section of our original manuscript, where we have discussed differences between it and MR-SimSS. For example, "RIVW… requires that the exposure and outcome GWASs have been performed with non-overlapping samples and is vulnerable to unbalanced pleiotropy. In contrast… MR-SimSS can mitigate Winner's Curse bias irrespective of sample overlap and can also be used with existing MR methods that are resistant to weak instrument bias and certain types of pleiotropy.".

5. **Do the authors have plans to implement this in/integrate this with TwoSampleMR (after hopefully taking in my suggestions above)?**
Yes, we certainly hope to – we are currently working with the second author to make this a reality.

6. **I did not get the "probabilistic variant pre-selection" part. It looks like a deterministic selection procedure so I have doubts about whether it can be justified.**
We thank the reviewer for stating their concern here. It is true that "deterministic procedure" is a better description and thus, we have updated the sentence where this is mentioned in the manuscript accordingly. We have also updated the corresponding section "Variant pre-filtering" in the Supplementary Note to provide a more complete argument of the strategy.

7. **The authors should compare their real data results in "effect of body mass index on type II diabetes" with true three-sample estimates.**
   A thorough response to this comment is provided at the end of this document, in conjunction with a response to Reviewer #3's Comment #8.

8. **Line 61: "most MR analyses now use a sample, or summary-level, design." Confusing, why "or"?**
   This has been changed to reflect reviewer's suggestion, and now reads as ".. most MR analyses now use a summary-level design".

9. **Line 83: "A genetic variant is considered a weak instrument if it explains only a small proportion of variation in the exposure". This is not quite right. "Weakness" is also relative to the sample size.**
   We thank the reviewer for this helpful clarification. We agree that instrument strength is relative to sample size, and have revised the wording accordingly to reflect that: "A genetic variant is considered a weak instrument if it has a weak statistical association with the exposure relative to the sample size, resulting in finite-sample bias in the causal effect estimate."

10. **Line 86: "In a complete overlap (one-sample) setting, causal estimates are biased toward the observational association, increasing the risk of Type I error." The term "Type I error" is from hypothesis testing and is ambiguous in this setting about causality. I would say something like "... are biased toward the observational association, which may increase false positive findings".**
    This has been changed to reflect reviewer's suggestion.

Reviewer #3:

The paper presents an original and smart idea how to simulate summary statistics from sample splitting and uses this to reduce Winner's curse bias in Mendelian Randomisation. I find the m/s well-written, the idea truly original and the results are sound. While it does not seem to be a game-changer for well-powered MR studies, it has numerous merits and deserves publication. Below, I list a few comments that could help improving the paper.

1. **Since the authors already simulate a 3way split of the samples, can't they use the first split to select the SNPs and from the second split they could use the fraction "p" for the exposure and fraction "1-p" for the outcome and do the reverse as well? Would this ensure that there is no sample overlap?**
   We thank the reviewer for this thoughtful suggestion. Firstly, we note that due to "p" also being used to represent the proportion of causal variants in our simulation study, fraction "p" and fraction "$1 - p$" have been renamed "π_2" and "$1 - \pi_2$", respectively. With respect to the reviewer's comment, yes – in our current three-split framework, the

π_2-subset is used to supply variant-exposure association estimates ($\hat{\beta}_{X_j}^{2a}$), while the independent $(1-\pi_2)$-subset provides variant-outcome association estimates ($\hat{\beta}_{Y_j}^{2b}$). On each iteration of sample-splitting, $\left\{\hat{\beta}_{X_j}^{2a}, \sigma_{X_j}^{2a}, \hat{\beta}_{Y_j}^{2b}, \sigma_{Y_j}^{2b}\right\}$ are then input into the 2-sample MR method of choice. It is certainly possible to "do the reverse as well" as the reviewer suggested, i.e. obtain variant-exposure association estimates from the $(1-\pi_2)$-subset ($\hat{\beta}_{X_j}^{2b}$) and obtain variant-outcome association estimates from the π_2-subset ($\hat{\beta}_{Y_j}^{2a}$). Therefore, our approach could be adapted so that on each sample-splitting iteration, $\left\{\hat{\beta}_{X_j}^{2a}, \sigma_{X_j}^{2a}, \hat{\beta}_{Y_j}^{2b}, \sigma_{Y_j}^{2b}\right\}$ are first input into the 2-sample MR method to provide a causal effect estimate and following this, the 2-sample MR method is also applied to $\left\{\hat{\beta}_{X_j}^{2b}, \sigma_{X_j}^{2b}, \hat{\beta}_{Y_j}^{2a}, \sigma_{Y_j}^{2a}\right\}$ to obtain another estimate of the causal effect. Thus, for a given number of iterations (N_{iter}), this adaptation would essentially provide us with double the amount of causal effect estimates which are then averaged over to provide the final estimate, $\hat{\beta}$ (see Equation (3) in the manuscript). If N_{iter} were small, less than 100 say, this adaptation would no doubt ensure greater stability of our final estimate and lead to a reduction in the standard error. However, N_{iter} is already set at $N_{iter} = 1000$ and thus, we anticipate that adapting the MR-SimSS algorithm in this manner would likely only provide a marginal benefit while increasing the runtime of the algorithm. To illustrate this, we have performed a small simulation study (100 replicates) in which we considered the fixed setting: sample size ($n_X = n_Y = 200{,}000$), exposure heritability ($h^2 = 0.7$), proportion of causal variants ($p = 0.01$), exposure-outcome correlation ($\rho = 0.5$), exposure-outcome causal effect ($\beta = 0.3$), full sample overlap ($n_{overlap} = 1 \cdot n_X$). In the figures below, SimSS-3-RAPS-A represents our original definition of the approach while SimSS-3-RAPS-B represents the aforementioned adaptation of MR-SimSS. Fig. A) illustrates how extremely similar the two methods are, with respect to estimating the causal effect, while Fig. C) depicts the greater runtime of SimSS-3-RAPS-B. Fig. B) shows that while SimSS-3-RAPS-B may lead to reduced variability in the standard error, there doesn't seem to be any empirical evidence that it will provide a significantly smaller standard error when using such a large number of iterations ($N_{iter} = 1000$).

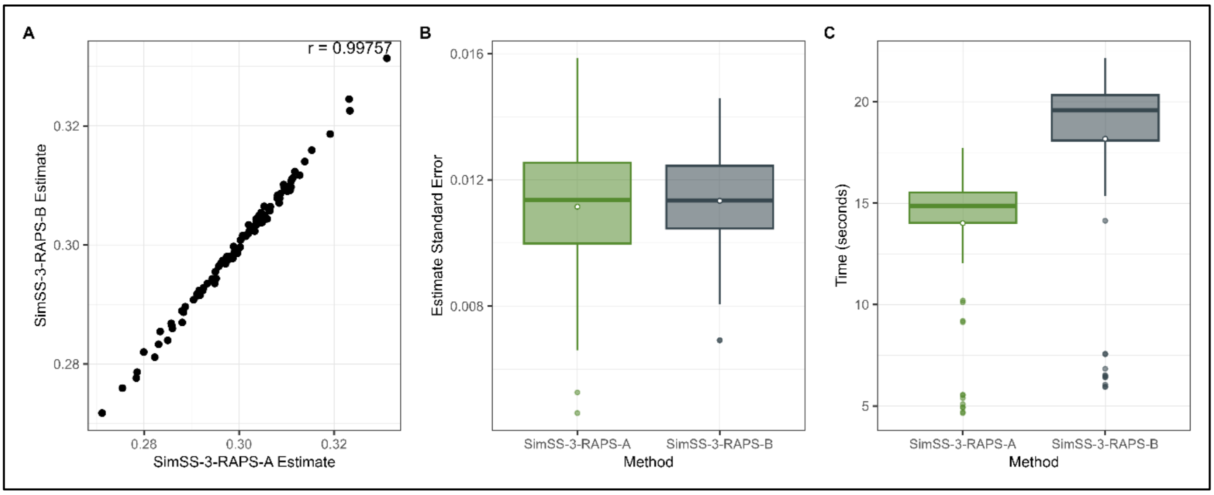

2. **The simulation exercise seems to be too simple, there is no pleiotropy introduced, while in real settings both correlated and uncorrelated pleiotropy is frequent. These need to be explored.**
   We thank the reviewer for this comment. We have now extended our simulation study to include settings in which uncorrelated pleiotropy, both balanced and directional, and correlated pleiotropy was present, with non-overlapping and fully overlapping samples. Parameters were fixed across settings as follows: sample size ($n_X = n_Y = 200{,}000$), exposure heritability ($h^2 = 0.7$), proportion of causal variants ($p = 0.01$), exposure-outcome correlation ($\rho = 0.5$) and exposure-outcome causal effect ($\beta = 0.3$). First, uncorrelated pleiotropy was incorporated by introducing variant-specific direct effects α_j on the outcome, such that the true variant-outcome association satisfied $\beta_{Y_j} = \beta \cdot \beta_{X_j} + \alpha_j$. For pleiotropic variants, α_j was drawn independently of the variant-exposure effect β_{X_j} from a normal distribution with mean 0 (balanced pleiotropy) or 0.2 (directional pleiotropy) and unit variance, ensuring $\mathrm{cov}(\alpha_j, \beta_{X_j}) = 0$. Pleiotropy was restricted to a proportion $p_\alpha = 0.2$ (20%) of exposure-associated variants and rescaled to explain a fixed fraction ($h^2_{\mathrm{pleio}} = 0.05$) of the outcome variance. Correlated pleiotropy was then introduced by generating variant-specific direct effects that were correlated with instrument strength. For a proportion $p_\alpha = 0.2$ of exposure-associated variants, pleiotropic effects were generated as $\alpha_j = \lambda \cdot \beta_{X_j} + \varepsilon_j$ where $\varepsilon_j \sim N(0.2, 1)$ and λ was selected to achieve $\mathrm{corr}(\alpha, \beta_X) = 0.2$ (positive correlation) or $\mathrm{corr}(\alpha, \beta_X) = -0.2$ (negative correlation). As in the uncorrelated setting, pleiotropic effects were rescaled to explain 5% of the outcome variance.

   We have now noted in the manuscript that additional simulations have been conducted under pleiotropy, with corresponding figures (Supplementary Figs. 12-15) and tables (Supplementary Tables 11-16) included in the Supplementary Note. These results further demonstrate MR-SimSS' ability to avoid Winner's Curse and overlap-induced bias, while retaining the properties of the embedded two-sample MR method. As MR-RAPS itself is not resistant to correlated pleiotropy, we do not expect SimSS-3-RAPS to be robust in these simulated correlated pleiotropic settings. However, MR weighted median is theoretically resistant to pleiotropy-induced bias in these settings as more than 50% of instruments should be valid IVs. Therefore, we have also included an additional variant of MR-SimSS in the auxiliary study in which MR-SimSS is combined with the MR weighted median estimator (SimSS-2-Med and SimSS-3-Med). Supplementary Tables 14 & 16 have been included to assist in the interpretation of Supplementary Figs 14-15. We can see that these implementations of MR-SimSS (SimSS-2-Med and SimSS-3-Med) clearly exhibit resistance to Winner's Curse and pleiotropy-induced bias. However, their estimates remain biased downwards due to the weighted median's susceptibility to weak instrument bias. Note that we have added additional comments regarding the above to the Discusssion section of the manuscript.

3. **Another relevant methods, such as MRlap [https://pubmed.ncbi.nlm.nih.gov/37036286/] would be interesting to test against MR-SimSS, since it claims to handle sample overlap and winner's curse related biases.**
We thank the reviewer for raising this point and agree that it would be interesting to compare MRlap to MR-SimSS. However, in order to estimate underlying parameters required to correct the naïve/classical IVW estimator, both distance pruning and LDSC analyses are incorporated into the MRlap algorithm. As our simulation study involves the direct simulation of GWAS summary statistics, MRlap therefore cannot be included in the study (MRlap algorithm requires additional genome-wide information). Despite this, we have included MRlap in our same-trait (BMI-BMI) empirical analyses (see Fig. 3 and Table 2 in the main manuscript), and have updated the corresponding Results section accordingly.

4. **The authors should investigate the power of these methods. Given the settings, IVW would not produce false positives, thus its discovery power is key to be compared to that of MR-SimSS. Simulations show a very small downward bias of IVW (~3% relative bias), but 25% lower SE compared to MR-SimSS, which implies superior power for IVW.**
We thank the reviewer for sharing their concern regarding the power of the methods. Firstly, we would like to draw the reviewer's attention to Supplementary Fig. 5 and Supplementary Table 4 which show the results obtained when simulations were conducted with fully overlapping samples under the null, i.e. true causal effect β = 0. It is well known that the combination of sample overlap and weak instrument bias will lead to inflated Type I error for IVW when the exposure-outcome relationship is confounded in the population. In fact, this also extends to methods that are robust to weak instrument bias under no sample overlap, such as MR-RAPS. Supplementary Table 4 shows that across the simulated settings with β = 0, the application of naïve IVW will produce extra false positives as its false positive rate is consistently much greater than 0.05 [range: 0.22-0.67]. In contrast, our simulations demonstrate that MR-SimSS in its three-split form, when paired with MR-RAPS/IVW, has roughly 95% coverage, and consequentially 5% Type I error. As we have stated in our discussion section, we feel that this is an important advantage of our proposed sample splitting approach which should be emphasised. Under general conditions, it is essential that MR methods have preserved Type I error, particularly given what has been referred to as a 'credibility crisis' in Mendelian Randomization [Burgess et al. (2024)].

However, it is true that in similar simulated settings with no sample overlap, IVW would likely have a similar false positive rate to MR-SimSS but would also have greater discovery power. In order to address the reviewer's concern regarding an investigation of power, we have performed additional simulations. We evaluated power under a fixed baseline simulation setting by varying only the true causal effect size, β, while holding all other parameters constant: sample size ($n_X = n_Y = 200{,}000$), exposure heritability

$(h^2 = 0.3)$, proportion of causal variants $(p = 0.01)$, exposure-outcome correlation $(\rho = 0.5)$. Both zero sample overlap $(n_{overlap} = 0)$ and complete overlap $(n_{overlap} = 1 \cdot n_X)$ scenarios were considered. The true causal effect, β, was fixed at: $\beta \in \{0, 0.01, 0.02, 0.03, 0.04, 0.05, 0.06, 0.07, 0.08\}$. For each value of β, $N_{\text{sim}} = 100$ independent simulation replicates were generated and the following methods: SimSS-3-IVW, SimSS-3-RAPS, IVW and RAPS, were applied to the simulated summary data. Power was estimated as the empirical probability of rejecting the null hypothesis, defined as the proportion of simulation replications in which the corresponding p-value was less than or equal to 0.05:

$$\widehat{\text{Power}}(\beta) = \frac{1}{N_{\text{sim}}} \sum_{k=1}^{N_{\text{sim}}} \mathbb{I}\{p_k(\beta) \leq 0.05\}$$

In Fig. 1 below, the estimated power is plotted against the true causal effect, β for each method. Fig. 2 is included as it can give additional context in some cases as to why certain values of estimated power were obtained.

As shown in Fig. 1, in settings with non-overlapping samples, appropriate Type I error control is exhibited by all methods as all rejection rates are less than or equal to 0.05 when the true causal effect is zero. While IVW and RAPS demonstrate superior power when the true causal effect is very small, power reaches saturation (power = 1) for all methods as the causal effect size increases beyond 0.05. However, when sample overlap is introduced, IVW and RAPS clearly fail to control Type I error. Even with a null causal effect, these methods generate a concerningly high rate of false positives, having reported a significant effect in approximately 100% of simulated settings. In contrast, both sample splitting approaches (SimSS-3-IVW, SimSS-3-RAPS) maintain correct Type I error rates while following a standard power trajectory as the effect size increases.

Causal effect estimation accuracy is visualised in Fig. 2. When sample overlap is zero and the true effect is small, all four methods seem to provide unbiased estimates. However, despite IVW and RAPS showing slightly higher precision (indicated by narrower interquartile ranges), bias towards the null is evident as the true causal effect increases. SimSS-3-RAPS maintains consistent estimation accuracy across the entire range of effect sizes. Under full sample overlap, IVW and RAPS demonstrate severe bias toward the observational correlation. Conversely, SimSS-3-RAPS remains unbiased, with median estimates aligning closely with the true causal effect.

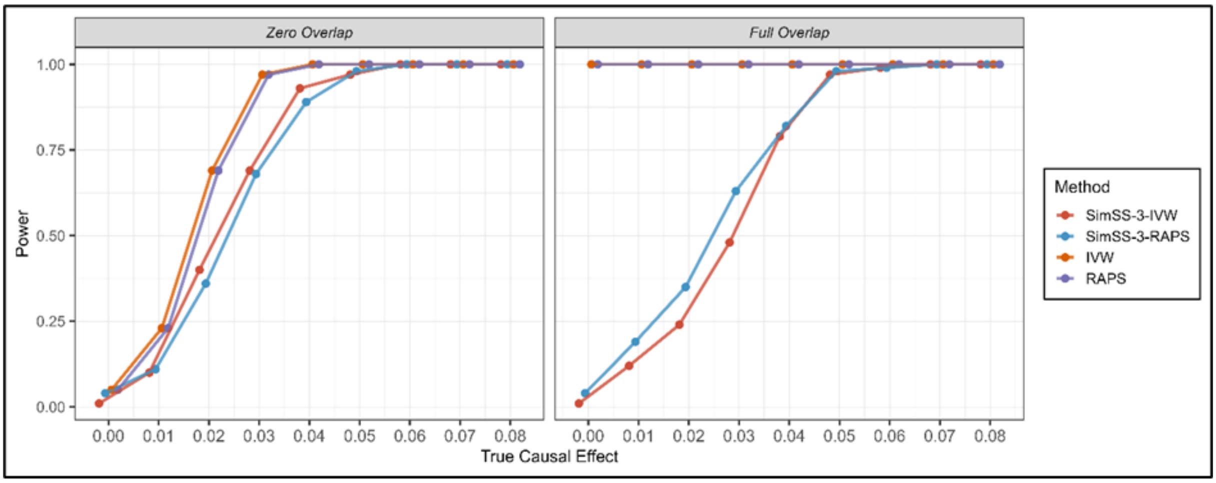

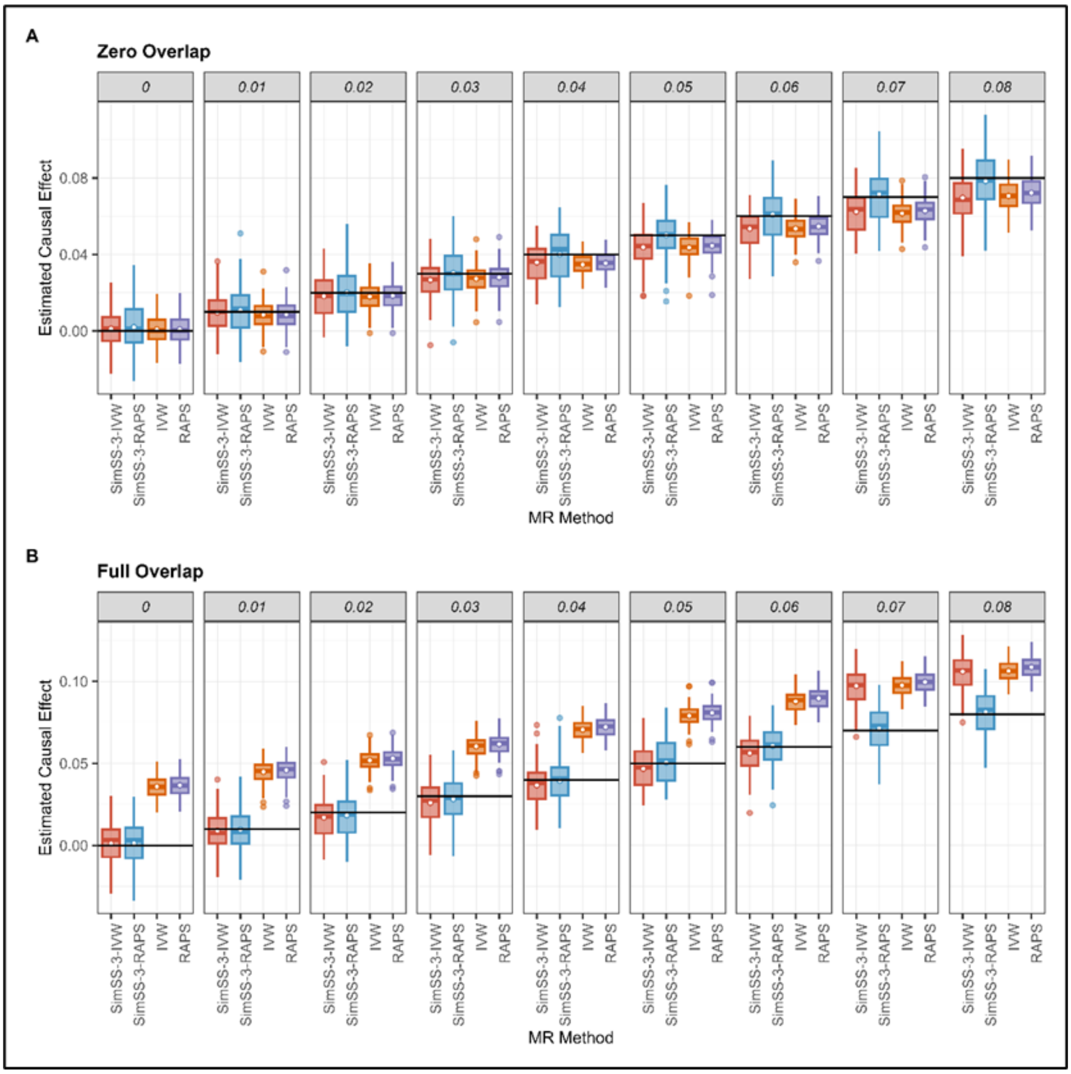

These two figures are now included in the Supplementary Note (Supplementary Figs. 12 and 13) and we have updated the following paragraph in the Discussion section: "Our simulations also demonstrate that MR-SimSS in its three-split variety, when paired with MR-RAPS has roughly 95% coverage, and consequentially 5% Type I error, under the null

hypothesis of no causal effect (Supplementary Fig. 5). While it is true that in zero overlap scenarios, classical MR methods, such as IVW, can demonstrate superior power over MR-SimSS variants when the true causal effect is small, these methods are highly susceptible to bias when sample overlap exists, resulting in greatly inflated Type I errors (Supplementary Figs. 12-13). This Type I error inflation is also seen for methods that are robust to weak instrument bias under no sample overlap, such as MR-RAPS, as we show in our simulations. In contrast, our study shows that SimSS-3-RAPS has the ability to retain a 5% Type I error, irrespective of the degree of sample overlap. Given what has been referred to as a 'credibility crisis' in Mendelian Randomization [25], it is essential that MR methods have preserved Type I error under general conditions, and thus, the MR-SimSS framework is an important development in this regard."

5. **Since the main disadvantage of MR-SimSS is the sample split, hence lower power, it should be emphasised more than the method is not recommended for MR with N below 100k (or less than 20 IVs)?**
   While we agree with the reviewer's premise, it is difficult to give good practical guidelines without running MR-SimSS first, since it depends primarily on the average number of variants chosen on each iteration, as opposed to the number of variants that are genome-wide significant originally. Instead, we recommend lowering the threshold for instrument selection if there are very few genome-wide variants. Note that using weak IVs will not create weak instrument bias, provided the three-split variants of MR-SimSS is combined with MR-RAPS.

6. **It should be acknowledged in the Discussion that the approach cannot help random over-/under-estimation of SNP effects that is present in the full sample (before splitting) and by definition their method will still preferentially select SNPs whose effects were overestimated in the full sample.**
   We thank the reviewer for this comment. While they are correct that the algorithm will preferentially select variants whose effects are overestimated in the full sample, this isn't a problem since the estimated associations for these variants are corrected for Winner's Curse. Note that the algorithm is probabilistically equivalent to sample splitting individual-level data, using the first split to select instruments, and the second split to estimate the associations for those instruments. The estimated associations in the first split will be overestimated due to Winner's Curse, but the algorithm doesn't use these in the estimation part. Due to sample splitting, the estimated associations in the second split (that are used in the MR meta-analysis) will be unbiased for their true associations, as can be seen in Equation (S28) of the revised Supplementary Note.

7. **While in the particular simulation setting using milder IV selection thresholds does not seem to have a detrimental impact on the causal effect estimation, in general it is rather dangerous to do, since it depends on the genetic architecture how many null SNPs are selected by mistake during this step, which dilutes the causal effect (or biases it in arbitrary direction in case of sample overlap). Thus, in general, this strategy should not be encouraged and readers must be warned of this in the Discussion.**

We thank the reviewer for this comment. However, we respectfully disagree here. Adding weaker instruments to the analysis, including possible null variants, doesn't 'dilute' the causal effect, provided we deal with the dual problems of weak instrument bias and sample overlap (this is demonstrated empirically in Supplementary Table 7). For instance, we have argued theoretically and shown empirically that the application of MR-SimSS in its three-split variety with MR-RAPS removes the intertwined biases from weak instruments and Winner's Curse, even under full sample overlap. The fact that there maybe more null-effect variants selected as instruments should not cause bias for the same reason, provided some genuine instruments are selected.

8. **The BMI->T2D results are not Discussed - I'd be curious to hear what conclusions can be drawn from those differences about the true causal effect and specific weaknesses of the methods.**
   A thorough response to this comment is provided below, in conjunction with a response to Reviewer #2's Comment #7.

9. **It is a bit confusing the \pi is used both to denote polygenicity, and also to refer to the sample split proportion. I'd introduce another variable for one of them.**
   We thank the reviewer for pointing this out. We agree here that different notation should have been used and therefore, we have changed the sample split proportions to π_1 and π_2, with p now denoting polygenicity throughout the manuscript.

<u>**Reviewer #2:**</u>

7. **The authors should compare their real data results in "effect of body mass index on type II diabetes" with true three-sample estimates.**

<u>**Reviewer #3:**</u>

8. **The BMI->T2D results are not Discussed - I'd be curious to hear what conclusions can be drawn from those differences about the true causal effect and specific weaknesses of the methods.**

   We thank the reviewers for their comments regarding the section: "effect of body mass index on type II diabetes". In order to address these comments, we have attempted to augment our work as follows. In an effort to avoid any misleading result patterns, we have now also considered a second set of BMI-T2D analyses in which the second outcome T2D dataset contains the other half of UKBB samples not originally included in the first set. Therefore, for each overlap setting, two BMI-T2D MR analyses were conducted and the causal effect estimates were averaged. These pooled estimates are illustrated in Fig. 4 and provided in Table 3. The standard error value given is the average standard error across the two studies, which we note is a conservative approximation of

the standard error of the pooled effect estimate. This average standard error was used to compute the corresponding confidence intervals shown in Fig. 4.
In our study, the traditional IVW approach yielded average estimated effects ranging from 1.178, for non-overlapping samples, to 1.243, for fully overlapping samples. In contrast, the range of causal effect estimates provided by SimSS-3-RAPS is ~40% smaller, from 1.257 to 1.296. We also note that the standard deviation of SimSS-3-RAPS averaged estimates (0.014) was less than half of that of IVW (0.0282), giving evidence that SimSS-3-RAPS can provide more consistent effect estimates across different degrees of sample overlap between exposure and outcome data sets. The difference between SimSS-3-RAPS and IVW estimates was greatest in the zero overlap setting, with our results suggesting that IVW underestimated the causal effect by ~8% due to downward bias, potentially caused by both Winner's Curse and weak instruments. If we were to repeat this splitting of UKBB and estimation of the BMI-T2D causal effect several times, we anticipate that SimSS-3-RAPS' estimation consistency would be even more evident.

Unfortunately, as we have used the entire UKBB for these analyses by splitting it in half, the consideration of a three-sample design in which another fraction of the UKBB is used solely for instrument selection purposes is currently outside the scope of our work. However, we did conduct a separate "three-sample" MR study in which we used the BMI GWAS dataset from the GIANT Consortium to select instrument variants. The MR analysis with our UKBB GWAS summary statistics derived from non-overlapping samples was then repeated. There were 151 genetic variants which had p-values less than the genome-wide significance threshold in the GIANT dataset that were also contained in our pruned UKBB datasets. This analysis provided a three-sample IVW estimate of 1.22 [95% CI: 1.14-1.31]. However, as there may be overlapping samples between GIANT and UKBB, it is possible that this estimate may suffer from some Winner's Curse bias, in addition to weak instrument bias toward the null. Our results above suggest that a three-sample estimate corrected for weak instrument bias, in which the three samples are truly independent, is more likely to be in the approximate region of 1.25-1.30.

As an alternative benchmark, we consider applying the debiased IVW (dIVW) method to the zero sample-overlap settings. As mentioned in a previous reviewer reply, our simulations in the original manuscript showed that dIVW can provide unbiased estimates (i.e. estimates free from both Winner's Curse and weak instrument bias) in settings with non-overlapping samples. Our empirical same-trait analyses reaffirmed this characteristic of dIVW as it achieved minimal bias, yielding causal effect estimates very close to the true effect of 1 (see Fig. 3). However, the dIVW method is highly sensitive to sample overlap and may provide miscalibrated standard errors. The dIVW approach controls weak instrument bias by explicitly correcting for measurement error in the variant-exposure associations. Furthermore, it is designed to not require screening (i.e. selection of variants most strongly associated with exposure) and so, doesn't suffer from selection bias due to Winner's Curse. Therefore, dIVW causal effect estimates obtained with independent samples should theoretically be similar to those

provided by SimSS-3-RAPS in all overlap settings. Applying dIVW to both T2D and BMI sets of summary statistics which had 0% overlap yielded an average estimate of 1.2673. This dIVW estimate lies within the range of estimates provided by SimSS-3-RAPS, providing further evidence that SimSS-3-RAPS is mitigating both Winner's Curse and weak instrument bias.

In a previous BMI-T2D MR study, Corbin et al. (20) obtained estimates corresponding to an OR for T2D per unit increase in BMI (kg/m^2) of 1.19, 1.26 and 1.38 for the IVW, weighted median and MR-Egger analyses, respectively. We note that this was a two-sample MR study which aimed to avoid sample overlap in the exposure and outcome datasets. Our work here suggests that this IVW estimate likely suffers from Winner's Curse and the true BMI-T2D causal effect is potentially larger.

We note that the corresponding Results section has been updated, and a paragraph has been added to the Discussion section regarding the analyses mentioned above.
