# Supplementary material for: Simulated sample splitting approach to address biases due to instrument selection and participant overlap in two-sample Mendelian Randomization studies
Source: PLoS Genet. 2026 May 8;22(5):e1011949. doi: 10.1371/journal.pgen.1011949 (PMC13178975; doi:10.1371/journal.pgen.1011949)

Amanda Forde
School of Mathematical and Statistical Sciences
University of Galway
Galway
Ireland
Email: amanda.forde@universityofgalway.ie
Thursday, 23rd April 2026

Dear Editors,

We are pleased to present a revised version of our manuscript "Simulated sample splitting approach to address biases due to instrument selection and participant overlap in two-sample Mendelian Randomization studies".

We would like to again express our gratitude to the reviewers for their helpful and comprehensive comments. We have considered their new comments in detail and provide a point-by-point response below.

We look forward to hearing back from you soon.

Yours sincerely,
Amanda Forde

<u>Reviewer #1:</u>

Thank you for addressing my comments.

1. **One possible error: on line 217 of the clean version, I don't think the square root sign should extend over sigma.**
   We thank the reviewer for noticing this error. We have now amended line 217 so the square root sign no longer extends over sigma, i.e. $z_j = \frac{\hat{\beta}_{X_j}}{\sqrt{\frac{1}{\pi_1}} \cdot \sigma_{X_j}}$.

<u>Reviewer #3:</u>

I congratulate the authors on their thorough revision, greatly addressing my points. In case the decision is a minor revision, I have a small comment, but it is not crucial to address:

1. **"The fact that there maybe more null-effect variants selected as instruments should not cause bias for the same reason, provided some genuine instruments are selected." – I would thus include a warning that applying this (or any other MR approach blindly) even if there are no genome-wide significant instruments are present is not recommended.**
   We thank the reviewer for this comment. We agree that MR methods should not be applied blindly in settings where are no clear variant-exposure associations. Under a Winner's Curse and weak instrument bias–corrected framework, and in the absence of directional pleiotropy, MR-SimSS would exhibit extremely low power, regardless of the magnitude of the true causal effect. In fact, in the non-overlap case, if all of the true variant-exposure associations are exactly zero, the ratio estimators at a per-variant level, would be the ratio of two zero mean normals and have scaled Cauchy distributions, indicating that Type I error will also be wrong. We've added a note to the manuscript at line 491 to reflect this: "However, this logic only extends so far. For instance, we would not recommend applying MR-SimSS blindly if there are no genome-wide exposure-associated variants. MR-SimSS with a reduced selection threshold is likely to have extremely low power in this setting, even under large exposure-outcome causal effects, and possibly inflated Type I error".

<u>Reviewer #2:</u>

I have two remaining comments:

1. **It's an interesting proposal to use LD pruning to address my second comment. I still think LD clumping after injecting correlated noise is likely a better approach, but understand the computational concern. But I'm worried about the power of LD pruning – in my experience, if one sets a loose LD threshold, LD pruning tends to produce correlated instruments and methods assuming independent instruments tend to underestimate the standard error. Do the authors observe something similar in their BMI-BMI analysis (or simulation analyses)?**

We thank the reviewer for this comment. In order to address this comment, we focussed on the BMI-BMI analysis included in the manuscript's supplementary material which compared the use of variants under LD-clumping and LD-pruning (Supplementary Table 17, Supplementary Fig. 18). Fixing the LD window size at 100kb, we varied the pruning threshold $r^2 \in \{0.001, 0.005, 0.01, 0.05, 0.1, 0.2, 0.5\}$ and repeated the MR analysis in each instance. The results are available in Table A and Figure A below. The reviewer correctly noted that if an MR method is applied to correlated instruments, then the standard error of the MR estimate will likely be underestimated. In order to gain an insight into the potential severity of this issue, we obtained a very rough approximation of the inflation in the variance of the IVW estimate due to correlation as follows.

The IVW estimate is $\hat{\beta}_{\text{IVW}} = \dfrac{\sum_i w_i \frac{\hat{\beta}_{Y_i}}{\hat{\beta}_{X_i}}}{\sum_i w_i}$ where $w_i = \widehat{\text{var}}\left[\frac{\hat{\beta}_{Y_i}}{\hat{\beta}_{X_i}}\right]^{-1} \approx \left(\frac{1}{\beta_{X_i}{}^2}\text{se}(\hat{\beta}_{Y_i})^2\right)^{-1} = \frac{\beta_{X_i}{}^2}{\text{se}(\hat{\beta}_{Y_i})^2}$. Therefore, $\text{var}(\hat{\beta}_{\text{IVW}}) = \frac{1}{(\sum_i w_i)^2}\text{cov}\left[\sum_i w_i \frac{\hat{\beta}_{Y_i}}{\hat{\beta}_{X_i}}, \sum_i w_i \frac{\hat{\beta}_{Y_i}}{\hat{\beta}_{X_i}}\right]$.
Treating $\hat{\beta}_{X_i}$ as fixed according to the 'NOME' assumption (i.e. $\hat{\beta}_{X_i} = \beta_{X_i}$), we thus have the following:

$$\begin{aligned}\text{var}(\hat{\beta}_{\text{IVW}}) &\approx \frac{1}{(\sum_i w_i)^2}\left[\sum_i \frac{w_i^2}{\beta_{X_i}{}^2}\text{se}(\hat{\beta}_{Y_i})^2 + \sum_{i\neq j}\frac{w_i w_j \rho_{ij}}{\beta_{X_i}\beta_{X_j}}\text{se}(\hat{\beta}_{Y_i})\text{se}(\hat{\beta}_{Y_i})\right] \\ &= \frac{1}{(\sum_i w_i)^2}\left[\sum_i \frac{\beta_{X_i}{}^2}{\text{se}(\hat{\beta}_{Y_i})^2} + \sum_{i\neq j}\frac{\beta_{X_i}\beta_{X_j}}{\text{se}(\hat{\beta}_{Y_i})\text{se}(\hat{\beta}_{Y_i})}\rho_{ij}\right] \\ &= \frac{1}{(\sum_i w_i)^2}\left[\sum_i w_i + \sum_{i\neq j}\frac{\beta_{X_i}\beta_{X_j}}{\text{se}(\hat{\beta}_{Y_i})\text{se}(\hat{\beta}_{Y_i})}\rho_{ij}\right]\end{aligned}$$

Assuming all variants have equal minor allele frequencies ($se(\hat{\beta}_{Y_i}) = s$ are constant) and equal effects on the exposure ($\beta_{X_i} = \beta_X$ are also constant), then $\text{var}(\hat{\beta}_{\text{IVW}}) = \text{var}(\hat{\beta}_{\text{IVW, independent}}) \cdot [1 + (N_{\text{sig}} - 1)\bar{\rho}]$.
This shows that given the assumptions, the relative inflation in variance due to correlation can be roughly approximated by $[1 + (N_{\text{sig}} - 1)\bar{\rho}]$ where N_{sig} is the number of genome-wide significant variants (variants selected for MR analysis) and $\bar{\rho}$ is the average correlation between those variants. The confidence intervals for the IVW estimate should then by inflated by a rough factor of $\sqrt{1 + (N_{\text{sig}} - 1)\bar{\rho}}$. As can be seen in Table B, this inflation factor was computed for each LD threshold r^2. Furthermore, the ratio of significant variants remaining after pruning versus after clumping is also included in Table B.
The results in Table B were somewhat surprising to us, and indicate that clumping is substantially more powerful than pruning, resulting in roughly 3 times as many genome-wide significant signals. In addition, manual evaluation of r^2 in the genome-wide significant variants after pruning, indicated several variant pairs with sizeable correlation (for instance, 0.5 or 0.6), and possible deflated estimates of the standard error by a factor of 1.3/1.4. It seems to us that the default PLINK pruning window seems too narrow for MR analyses in UK Biobank. For this reason, while we didn't manually prune the variants in the analyses in the main manuscript, in future work we would

recommend doing so if pruning is used. Note that MR-SimSS simulated per-split associations independently for each variant. The independent nature of the simulations weakens per-variant correlations in the association estimates at a particular splits if variants are in LD. For instance, in individual data, with a default 50% split for instrument selection, 25% for estimation of exposure associations and 25% for outcome associations, the overlap between the samples in split 2 for variant A and variant B would be 25% if the split was performed independently at each variant, in analogy with MR-SimSS. This would reduce the covariance between the estimates of variant A and B by a factor of ¼, and consequently, the correlation by a factor of ½. This implies that, conditional on the chosen variants on split 1, the approximate factor by which the measured standard error should be inflated by on a typical MR-SimSS iteration may be, very roughly, $\sqrt{1+\frac{(N_{\mathrm{sig}}-1)}{2}\bar{\rho}}$ rather than $\sqrt{1+(N_{\mathrm{sig}}-1)\bar{\rho}}$, where N_{sig} is the number of significant variants on the first split. For instance, in table B below, with pruning with an LD threshold of 0.001, the standard error inflation factor is 1.37 for IVW run on the full dataset, but maybe only 1.05 with MR-SimSS, if $N_{\mathrm{sig}} = 7$ and $\bar{\rho} = 0.02775$.

2. **I hope the authors will consider publishing the response letter which in my opinion contains useful information for users and methodologists.**
   Yes, we would be happy to also publish the response letter, alongside the manuscript.

Table A: Summarized results for each method resulting from a same-trait BMI-BMI analysis using different sets of clumped and pruned variants (LD clumping via 'clump data()' function from 'twoSampleMR' R package using default parameters: 'clump kb' = 10,000, 'clump r2' = 0.001 and LD pruning via PLINK 2.0 using command 'indep-pairwise 100kb r^2').

Method	$\hat{\beta}$	SE	95% CI	# IVs
clumping: *r^2 = 0.001 (2,448 clumped variants)*				
**SimSS-2-IVW**	0.9491	0.0276	[0.895, 1.003]	21.37
**SimSS-3-IVW**	0.9307	0.0264	[0.879, 0.982]	21.40
**SimSS-2-RAPS**	0.9628	0.0381	[0.888, 1.038]	21.48
**SimSS-3-RAPS**	0.9649	0.0125	[0.940, 0.989]	21.48
**IVW**	0.8317	0.0239	[0.785, 0.878]	91
**RAPS**	0.8573	0.0193	[0.819, 0.895]	91
pruning: *r^2 = 0.001 (16,396 pruned variants)*				
**SimSS-2-IVW**	0.9832	0.0579	[0.870, 1.097]	7.17
**SimSS-3-IVW**	0.9469	0.0452	[0.858, 1.035]	6.99
**SimSS-2-RAPS**	1.0023	0.0578	[0.889, 1.116]	7.08
**SimSS-3-RAPS**	0.9966	0.0480	[0.903, 1.091]	7.13
**IVW**	0.9382	0.0376	[0.864, 1.012]	33
**RAPS**	0.9611	0.0340	[0.894, 1.028]	33
pruning: *r^2 = 0.005 (22,614 pruned variants)*				
**SimSS-2-IVW**	0.9905	0.0578	[0.877, 1.104]	6.89
**SimSS-3-IVW**	0.9435	0.0443	[0.857, 1.030]	6.92
**SimSS-2-RAPS**	1.0123	0.0509	[0.912, 1.112]	6.88
**SimSS-3-RAPS**	1.0188	0.0578	[0.906, 1.132]	6.90
**IVW**	0.9310	0.0379	[0.857, 1.005]	32
**RAPS**	0.9534	0.0343	[0.886, 1.021]	32
pruning: *r^2 = 0.01 (40,354 pruned variants)*				
**SimSS-2-IVW**	0.9918	0.0614	[0.872, 1.112]	7.00

**SimSS-3-IVW**	0.9424	0.0452	[0.854, 1.031]	6.91
**SimSS-2-RAPS**	1.0068	0.0558	[0.897, 1.116]	6.81
**SimSS-3-RAPS**	1.0119	0.0580	[0.898, 1.126]	6.96
**IVW**	0.9310	0.0379	[0.857, 1.005]	32
**RAPS**	0.9534	0.0343	[0.886, 1.021]	32
pruning: r^2 *= 0.05 (201,373 pruned variants)*				
**SimSS-2-IVW**	0.9834	0.0552	[0.875, 1.092]	7.75
**SimSS-3-IVW**	0.9360	0.0436	[0.851, 1.021]	7.73
**SimSS-2-RAPS**	1.0017	0.0593	[0.885, 1.118]	7.68
**SimSS-3-RAPS**	1.0023	0.0611	[0.882, 1.122]	7.80
**IVW**	0.9023	0.0326	[0.838, 0.966]	43
**RAPS**	0.9248	0.0304	[0.865, 0.984]	43
pruning: r^2 *= 0.1 (316,827 pruned variants)*				
**SimSS-2-IVW**	0.9749	0.0467	[0.883, 1.066]	9.27
**SimSS-3-IVW**	0.9326	0.0373	[0.859, 1.006]	9.19
**SimSS-2-RAPS**	0.9907	0.0526	[0.888, 1.094]	9.30
**SimSS-3-RAPS**	0.9980	0.0474	[0.905, 1.091]	9.19
**IVW**	0.9068	0.0294	[0.849, 0.965]	51
**RAPS**	0.9287	0.0281	[0.874, 0.984]	51
pruning: r^2 *= 0.2 (497,283 pruned variants)*				
**SimSS-2-IVW**	0.9813	0.0384	[0.906, 1.057]	12.31
**SimSS-3-IVW**	0.9357	0.0342	[0.869, 1.003]	12.21
**SimSS-2-RAPS**	1.0009	0.0484	[0.906, 1.096]	12.28
**SimSS-3-RAPS**	1.0022	0.0337	[0.936, 1.068]	12.43
**IVW**	0.8803	0.0262	[0.829, 0.932]	70
**RAPS**	0.9042	0.0240	[0.857, 0.951]	70
pruning: r^2 *= 0.5 (941,669 pruned variants)*				
**SimSS-2-IVW**	0.9793	0.0237	[0.933, 1.026]	32.69
**SimSS-3-IVW**	0.9335	0.0213	[0.892, 0.975]	32.76
**SimSS-2-RAPS**	0.9989	0.0299	[0.940, 1.057]	32.61
**SimSS-3-RAPS**	1.0004	0.0224	[0.956, 1.044]	32.72
**IVW**	0.8777	0.0161	[0.846, 0.909]	171
**RAPS**	0.8999	0.0152	[0.870, 0.930]	171

Figure A: Estimated causal effects for each method resulting from a same-trait BMI-BMI analysis using different sets of clumped and pruned variants (LD clumping via 'clump data()' function from 'twoSampleMR' R package using default parameters: 'clump kb' = 10,000, 'clump r2' = 0.001 and LD pruning via PLINK 2.0 using command 'indep-pairwise 100kb r^2').

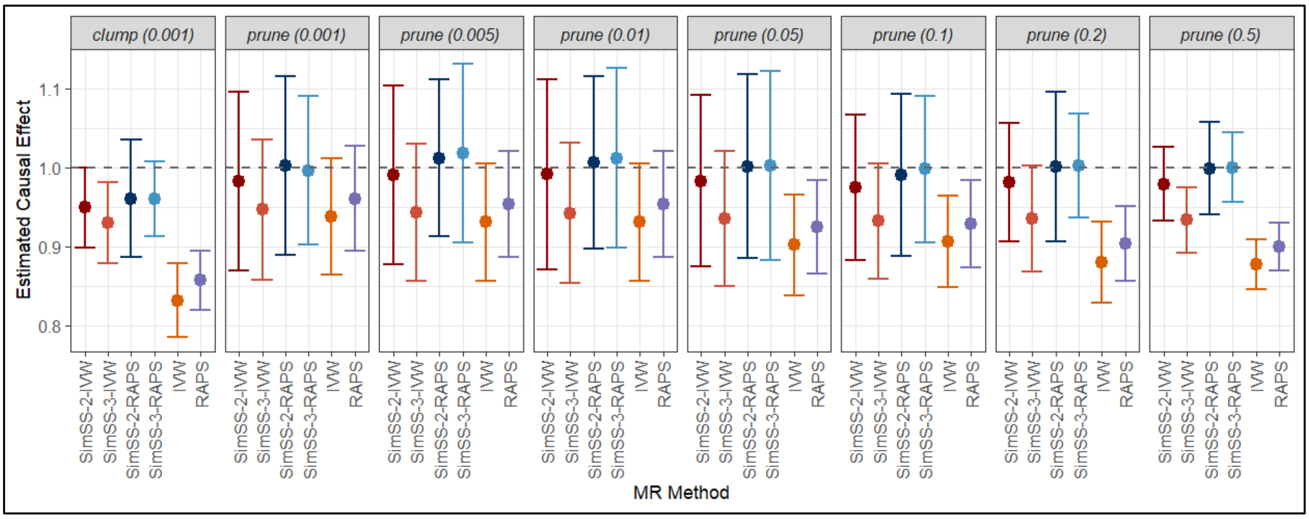

Table B: Average pairwise correlation between genome-wide significant variants in exposure BMI dataset for different sets of clumped and pruned variants (LD clumping via 'clump data()' function from 'twoSampleMR' R package using default parameters: 'clump kb' = 10,000, 'clump r2' = 0.001 and LD pruning via PLINK 2.0 using command 'indep-pairwise 100kb r^2').

	**# IVs (N_{sig})**	$N_{\text{sig}}^{\text{prune}} / N_{\text{sig}}^{\text{clump}}$	**Average pairwise r^2 among IVs** $\left(\sum_{i<j} r_{ij}^2 \Big/ \frac{N_{\text{IV}}(N_{\text{IV}}-1)}{2}\right)$	**Average correlation between IVs, $\bar{\rho}$**	$\sqrt{1+(N_{\text{sig}}-1)\bar{\rho}}$
clump $r^2 = 0.001$	91	-	0.00028	0.00318	1.1341
prune $r^2 = 0.001$	33	0.3626	0.01761	0.02775	1.3740
prune $r^2 = 0.005$	32	0.3516	0.01634	0.02589	1.3426
prune $r^2 = 0.01$	32	0.3516	0.01634	0.02589	1.3426
prune $r^2 = 0.05$	43	0.4725	0.01353	0.02343	1.4086
prune $r^2 = 0.1$	51	0.5604	0.00849	0.01707	1.3614
prune $r^2 = 0.2$	70	0.7692	0.00746	0.01565	1.4422
prune $r^2 = 0.5$	171	1.8791	0.00524	0.01271	1.7778

Figure B: Inflation factor ($\sqrt{1+(N_{\text{sig}}-1)\bar{\rho}}$) and ratio of pruned to clumped significant variants plotted against the various pruning thresholds (r^2) applied.

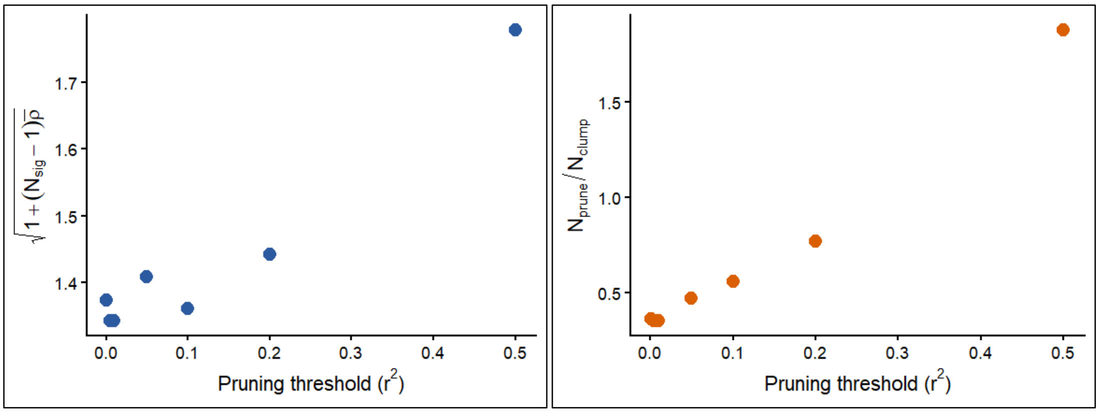

Supplement: Attachment — Submitted filename: MRSimSS-review-2.pdf [file pgen.1011949.s005.pdf]
